# Retroviral DNA Integration: ASLV, HIV, and MLV Show Distinct Target Site Preferences

**DOI:** 10.1371/journal.pbio.0020234

**Published:** 2004-08-17

**Authors:** Rick S Mitchell, Brett F Beitzel, Astrid R. W Schroder, Paul Shinn, Huaming Chen, Charles C Berry, Joseph R Ecker, Frederic D Bushman

**Affiliations:** **1**Department of Microbiology, University of Pennsylvania School of MedicinePhiladelphia, Pennsylvania, United States of America; **2**Gen-Probe, San DiegoCalifornia, United States of America; **3**Genomic Analysis Laboratory, The Salk InstituteLa Jolla, California, United States of America; **4**Department of Family/Preventive Medicine, University of California at San Diego School of MedicineSan Diego, CaliforniaUnited States of America

## Abstract

The completion of the human genome sequence has made possible genome-wide studies of retroviral DNA integration. Here we report an analysis of 3,127 integration site sequences from human cells. We compared retroviral vectors derived from human immunodeficiency virus (HIV), avian sarcoma-leukosis virus (ASLV), and murine leukemia virus (MLV). Effects of gene activity on integration targeting were assessed by transcriptional profiling of infected cells. Integration by HIV vectors, analyzed in two primary cell types and several cell lines, strongly favored active genes. An analysis of the effects of tissue-specific transcription showed that it resulted in tissue-specific integration targeting by HIV, though the effect was quantitatively modest. Chromosomal regions rich in expressed genes were favored for HIV integration, but these regions were found to be interleaved with unfavorable regions at CpG islands. MLV vectors showed a strong bias in favor of integration near transcription start sites, as reported previously. ASLV vectors showed only a weak preference for active genes and no preference for transcription start regions. Thus, each of the three retroviruses studied showed unique integration site preferences, suggesting that virus-specific binding of integration complexes to chromatin features likely guides site selection.

## Abstract 2

Retroviruses have potential for gene therapy only if they do not activate endogenous genes. Of three tested retroviral vectors, ASLV showed no preference for integration into human transcription start regions

## Introduction

Retroviral replication requires reverse transcription of the viral RNA genome and integration of the resulting DNA copy into a chromosome of the host cell. A topic of long standing interest has been the chromosomal and nuclear features dictating the location of integration target sites (reviewed in Coffin et al. 1997; Bushman 2001). Integration site selection has also gained increased interest because of its importance for human gene therapy. Retroviral vectors have been used extensively to deliver therapeutic genes carried in retroviral backbones. However, retroviral integration can take place at many locations in the genome, on occasion resulting in insertional activation of oncogenes (reviewed in Coffin et al. 1997; Bushman 2001). Recently, two patients undergoing gene therapy for X-linked severe combined immunodeficiency developed leukemia-like illnesses associated with integration of a therapeutic retroviral vector in or near the LMO2 proto-oncogene (Check 2002; Hacein-Bey-Abina et al. 2003). Insertional activation of oncogenes by retroviral vectors has also been detected in animal models (Li et al. 2002).

With the availability of the complete human genome sequence, large-scale sequence-based surveys of integration sites have become possible (Schroder et al. 2002; Laufs et al. 2003; Wu et al. 2003). Schroder et al. (2002) investigated targeting of human immunodeficiency virus (HIV) and HIV-based vectors in a human lymphoid cell line (SupT1) and found that genes were favored integration targets. Global analysis of transcription in SupT1 cells showed that active genes were favored for integration, particularly those that were active after infection with the HIV-based vector. Wu et al. (2003) examined targeting of murine leukemia virus (MLV) in human HeLa cells and found that MLV does not strongly favor integration in transcription units, but rather favors integration near sequences encoding mRNA 5′ ends. Here we contrast integration targeting by three retroviruses, avian sarcoma-leukosis virus (ASLV), HIV, and MLV, taking advantage of 1,462 new integration site sequences and matched transcriptional profiling data. We find that ASLV does not favor integration near transcription start sites, nor does it strongly favor active genes. For HIV, we find that active genes are favored for integration in two primary cell types, extending findings from previous studies of transformed cell lines. Cell-type-specific transcription was found to result in cell-type-specific biases in integration site placement. We also reanalyzed the MLV data from Burgess and coworkers (Wu et al. 2003) in parallel, confirming that MLV integration is favored near transcription start sites. Thus it appears that each retrovirus studied to date has a unique pattern of integration site selection within the human genome, suggesting that there may be local recognition of chromosomal features unique to each virus.

## Results

### Integration Site Datasets Used in this Study

**Figure 1:**
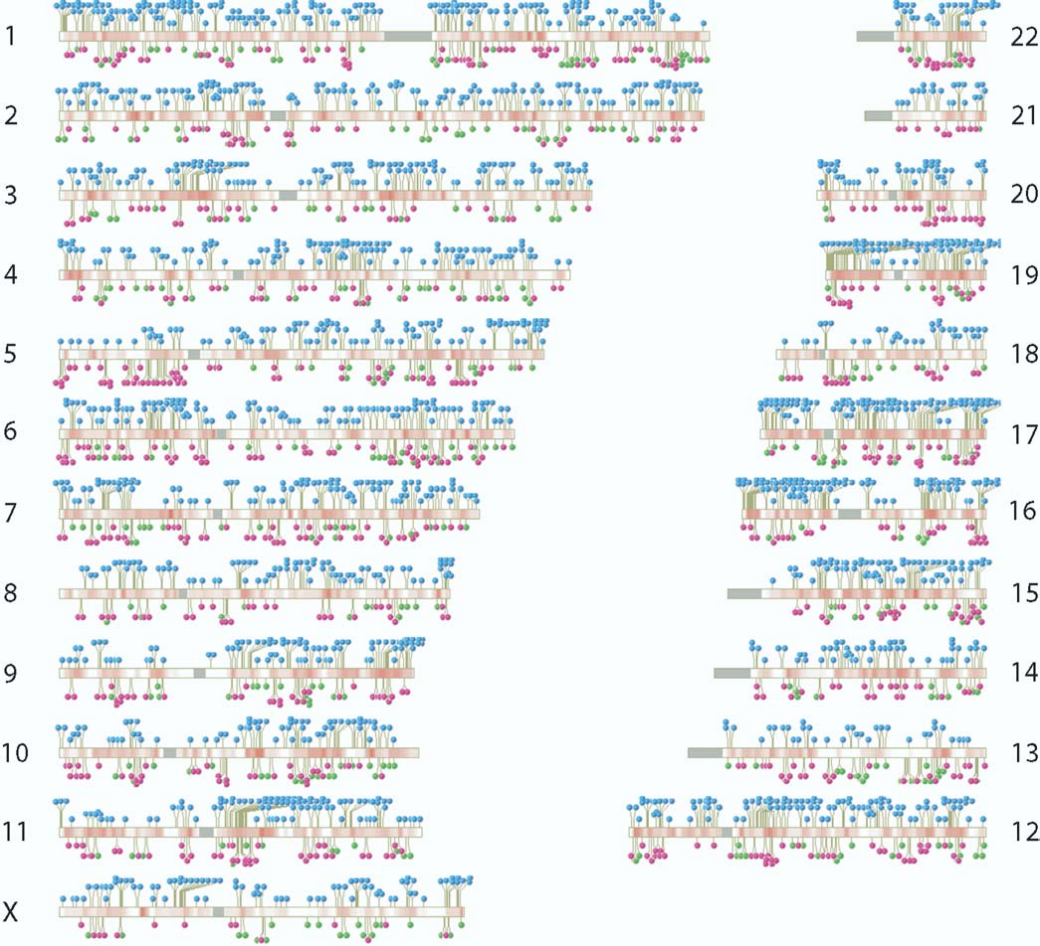
Relationship between Integration Sites and Transcriptional Intensity in the Human Genome The human chromosomes are shown numbered. HIV integration sites from all datasets in Table 1 are shown as blue “lollipops”; MLV integration sites are shown in lavender; and ASLV integration sites are shown in green. Transcriptional activity is shown by the red shading on each of the chromosomes (derived from quantification of nonnormalized EST libraries, see text). Centromeres, which are mostly unsequenced, are shown as grey rectangles.

The origins of the 3,127 integration sites studied are summarized in Table 1. All were generated by acute infection of cells with retroviruses or with viruses generated from retroviral vectors. To isolate integration sites, DNA from infected cells was isolated, cleaved with restriction enzymes, then ligated to DNA linkers. Integration sites were amplified using one primer that bound to the viral DNA end and another that bound the linker, then amplification products were cloned and sequenced (Schroder et al. 2002; Wu et al. 2003). Integration sites were mapped on the draft human genome sequence (Figure 1) and local features at integration sites quantified.

**Table 1:**
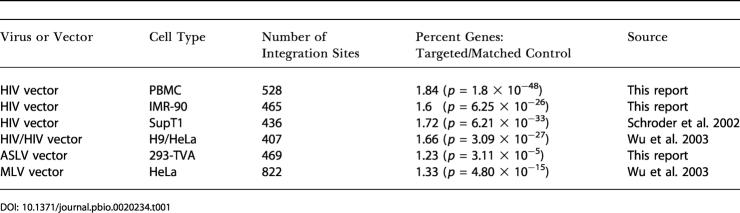
Integration Site Datasets Used in This Study

DOI: 10.1371/journal.pbio.0020234.t001

Three integration site datasets were newly determined in this study. Integration by an ASLV vector was analyzed in 293T-TVA cells, which are human 293T cells engineered to express the subgroup A avian retrovirus receptor. Integration by an HIV-based vector was characterized in two types of primary human cells, peripheral blood mononuclear cells (PBMCs) and IMR90 lung fibroblasts. Several previously described datasets were also subjected to further analysis in parallel—HIV integration sites in three transformed cell lines (SupT1 [Schroder et al. 2002], H9, and HeLa [Wu et al. 2003]) and MLV integration sites in HeLa cells (Wu et al. 2003).

The use of restriction enzymes to cleave cellular DNA during the cloning of integration sites could potentially introduce a bias in favor of isolating integration events closer to restriction sites. Previous work suggested that integration site surveys were not strongly biased. In one study, an experimental control based on integration in vitro indicated that the cloning and analytical methods used did not detectably bias the conclusions (Schroder et al. 2002). In addition, HIV integration sites cloned by different methods showed a similar preference for active genes (Carteau et al. 1998; Schroder et al. 2002; Wu et al. 2003), including sites isolated using several different restriction enzymes to cleave cellular DNA prior to linker ligation, and isolation using inverse PCR instead of ligation-mediated PCR.

In this study we have added a computational method to address possible biased isolation. Each integration site was paired with ten sites in the human genome randomly selected in silico that were constrained to be the same distance from a restriction site of the type used for cloning as the experimentally determined integration site. Statistical tests were then carried out by comparing each experimentally determined integration site to the ten matched random control sites. In this way any bias due to the placement of restriction sites in the human genome was accounted for in the statistical analysis. All the collections of integration sites were analyzed in this manner, including data previously published in (Schroder et al. 2002; Wu et al. 2003). However, direct analysis of the distribution of integration sites without this correction yielded generally similar conclusions, suggesting that restriction site placement did not introduce a strong bias. A detailed description of the statistical analysis is presented in Protocol S1 (p. 2).

### Integration in Transcription Units

For HIV the frequency of integration in transcription units ranged from 75% to 80%, while the frequency for MLV was 61% and for ASLV was 57%. For comparison, about 45% of the human genome is composed of transcription units (using the Acembly gene definition). Analysis using the different catalogs of human genes suggests that somewhat different fractions of the human genome are transcribed, and new information indicates that an unexpectedly large fraction of the human genome may be transcribed into noncoding RNAs (Cawley et al. 2004). However, for comparisons using any catalog of the human genes, the rate of integration in human transcription units determined experimentally was substantially higher than in the matched random control sites (Protocol S1, p. 3–11).

**Figure 2:**
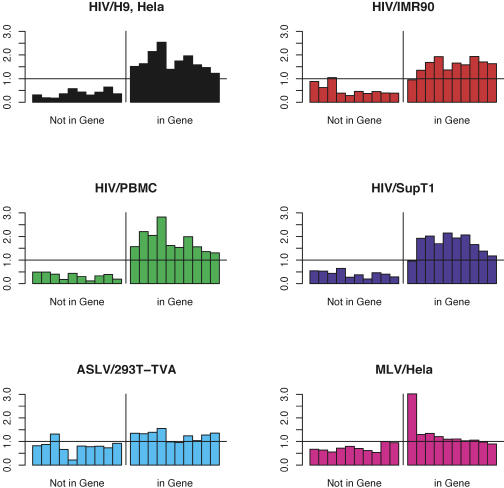
Integration Intensity in Genes and Intergenic Regions Genes or intergenic regions were normalized to a common length and then divided into ten intervals to allow comparison. The number of integration sites in each interval was divided by the number of matched random control sites and the value plotted. A value of one indicates no difference between the experimental sites and the random controls. Viruses and cell types studied are as marked above each graph. The direction of transcription within each gene is from left to right. Note that our normalization method de-emphasizes favored MLV integration events just upstream of gene 5′ ends (outside transcription units), as reported by Wu et al. (2003). We carried out an analysis specially designed to identify this effect and confirmed that the regions just upstream of gene 5′ ends are favored for MLV integration when reanalyzed with the matched random control data (unpublished data).

We next assessed the placement of integration sites within genes and intergenic regions. A previous study revealed that integration by MLV is favored near transcription start sites, but no such bias was seen for HIV (Wu et al. 2003). We investigated this issue for ASLV and reanalyzed all of the available data by comparison to the matched random control dataset (Figure 2). For sites within genes, MLV showed a highly significant bias in favor of integration near transcription start sites (*p* = 1.4 × 10^−14^). No such bias was seen for ASLV (*p >* 0.05). For the HIV datasets, two of the four sets showed modest bias (HIV/PBMC, *p =* 0.0007; HIV/H9 and HeLa combined, *p =* 0.027). For HIV, the observed statistical significance was sensitive to the details of analytical approach used and is of questionable biological importance. Thus, ASLV and HIV do not strongly favor integration at transcription start sites as was seen with MLV.

### Effects of Transcriptional Activity on Integration

Transcriptional profiling analysis was carried out in some of the cell types studied, allowing the influence of transcriptional activity on integration site selection to be assessed. Transcriptional profiling was carried out on infected cells so that the data reflected the known influence of infection on cellular gene activity (Corbeil et al. 2001; Schroder et al. 2002; Mitchell et al. 2003; van 't Wout et al. 2003). Prior to isolating RNA for microarray analysis, the SupT1, PBMC, and IMR90 cells were each infected with the HIV-based vector used for HIV integration site isolation. The 293T-TVA cells were infected with the RCAS ASLV vector prior to RNA isolation. RNA samples were harvested 24–48 h after infection. For analysis of MLV and HIV integration sites in HeLa cells, published transcriptional profiling data from uninfected cells were used (Tian et al. 2002).

**Figure 3:**
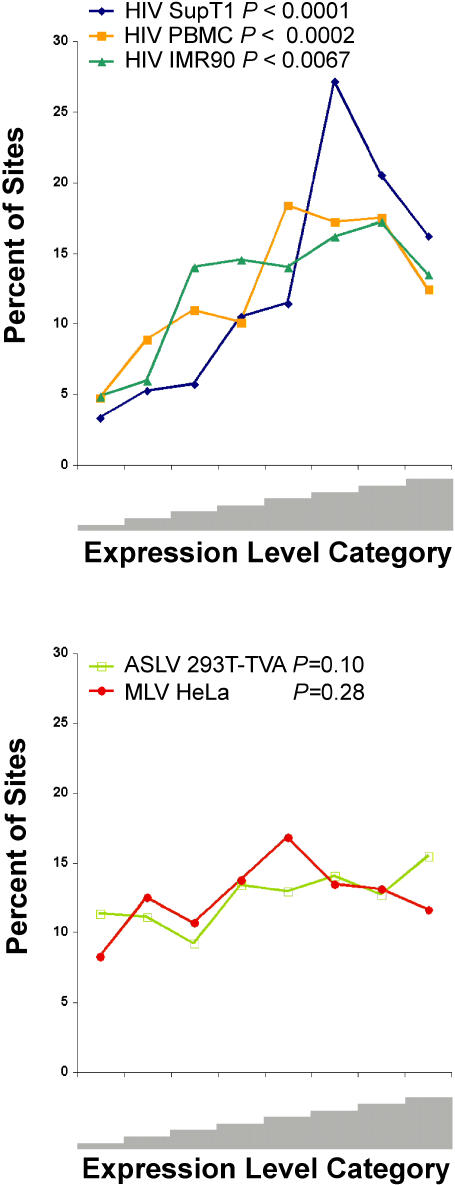
Influence of Gene Activity on Integration Frequency Expression levels were assayed using Affymetrix HU-95Av2 or HU-133A microarrays and scored by the average difference value as defined in the Affymetrix Microarray Suite 4.1 software package. All the genes assayed by the chip were divided into eight “bins” according to their relative level of expression (the leftmost bin in each panel is lowest expression levels and the rightmost the highest). Genes that hosted integration events were then distributed into the same bins, summed, and expressed as a percent of the total. The y-axis indicates the percent of all genes in the indicated bin. *P* values were determined using the Chi-square test for trends by comparison to a null hypothesis of no bias due to expression level. All average difference values were ranked prior to analysis, and the analysis was carried out on the ranked data. This was done to avoid possible complications due to differential normalization or other data processing differences arising during work up of the microarrays.

The median expression level (average difference value) of genes hosting integration events was consistently higher than the median of all genes assayed on the microarrays. The ratios (targeted genes/all genes assayed) for HIV ranged from 1.6 to 3.0, indicating that integration targeting in human primary cells (PBMC and IMR90) favored active genes, as shown previously for transformed cell lines (Schroder et al. 2002; Wu et al. 2003). For MLV and ASLV the ratios were each 1.3, lower than for HIV but still greater than the chip average. The analysis shown in Figure 3 reveals that the association of integration sites with gene activity was statistically significant for all the HIV datasets. For MLV and ASLV, there was a weak tendency for integration to favor active genes, but the trend did not achieve statistical significance.

All three HIV datasets showed reduced integration in the most highly expressed category of genes analyzed, suggesting that although transcription favors integration, very high level transcription may actually be less favorable (Figure 3). A fourth dataset monitoring infection by an HIV-based vector in a T-cell line also showed this trend (M. Lewinski, P. S., J. R. E., and F. D. B., unpublished data). Possibly this finding is related to that of a previous study of integration by ALV in a model gene that also suggested that high level transcription may disfavor integration (Weidhaas et al. 2000).

### Tissue-Specific Transcription Results in Tissue-Specific Biases in HIV Integration Site Selection

**Figure 4:**
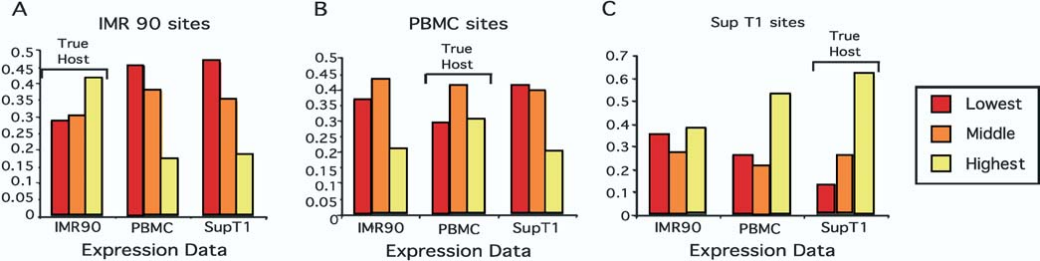
Effects of Tissue-Specific Transcription on Integration Site Selection in Different Cell Types Genes hosting integration events by the HIV vector were analyzed for their expression levels in transcriptional profiling data from IMR90, PBMC, and SupT1 cells. For each gene hosting an integration event, the expression values from the three cell types were then ranked lowest (red), medium (orange), and highest (yellow). The values were summed and displayed separately for each set of integration sites: (A) IMR90 sites, (B) PBMC sites, and (C) SupT1 sites. In each case there was a significant trend for the cell type hosting the integration events to show the highest expression values relative to the other two (*p <* 0.05 for all comparisons).

We next investigated the effects of cell-type-specific transcription on integration site selection. For this analysis we used only the three HIV integration site datasets for which we had transcriptional profiling data from infected cells (i. e., SupT1, PBMC, and IMR90), to allow us to control for the effects of infection on transcription. Pairwise comparisons of the microarray datasets for the three cell types showed that the correlation coefficients ranged only from 0.64 to 0.79, indicating that transcriptional activity indeed differed among cell types. We reasoned that since active transcription favors integration, then the genes targeted by integration should on average be more highly expressed in the cell type that hosted the integration event than in either of the other two. Statistical analysis (Figure 4) showed that transcription of targeted genes was higher on average in the cell type hosting the integration event than in either of the other two tested (all comparisons attain *p <* 0.05 using the Chi-square test for linear trend in proportions). We note, however, that the differences were quantitatively modest, perhaps because much of the cellular program of gene activity is shared among many cell types (Caron et al. 2001; Mungall et al. 2003; Versteeg et al. 2003).

### Integration Site Selection and Transcriptional Domains

**Figure 5:**
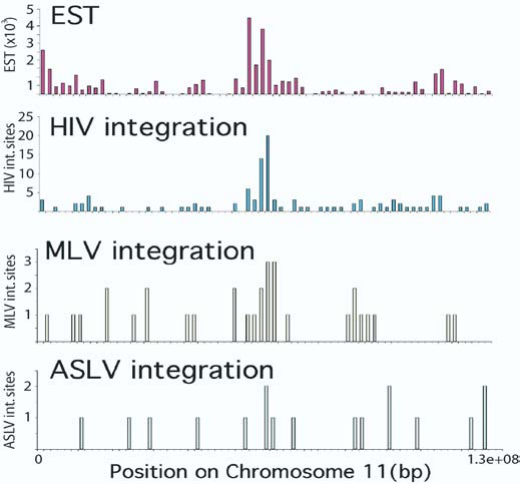
Comparison of Transcriptional Intensity to Integration Intensity on Human Chromosome 11 All data were quantified in 2-Mb intervals. The top line shows summed EST data documenting the “transcriptional intensity” for each chromosomal interval (data from Mungall and al. [2003]). The bottom three lines show the summed frequency of integration site sequences in each interval. The numbers of ESTs (top) or integration sites (bottom three) are shown on the y-axis.

We next analyzed factors influencing the placement of integration sites at the chromosomal level, taking into account both gene density and expression (see Figure 1). Transcriptional activity was quantified by counting the EST sequence copies for each gene present in a collection of nonnormalized EST libraries (Mungall et al. 2003). EST sequences from many tissues were used to build up the map of transcriptional activity, thus focusing the analysis on transcriptional patterns common to many cell types (Caron et al. 2001; Mungall et al. 2003; Versteeg et al. 2003). A detailed comparison of the relationship between EST frequency and integration frequency of HIV, MLV, and ASLV is shown for Chromosome 11 in Figure 5. Integration frequency for HIV closely parallels the transcriptional intensity deduced from EST counts. Fewer sites are available for analyzing MLV and ASLV, but MLV may show a related trend, while it is unclear whether ASLV does so or not. Similar analysis of the other human chromosomes yields similar conclusions (unpublished data).

Statistical analysis was carried out comparing integration frequencies to (1) gene density or (2) transcriptional intensity, as measured by the EST counts. All analyses incorporated a comparison to the matched random control set of integration sites. Each type of vector showed a significant positive correlation with gene density (HIV, *p =* 1.8 × 10^−12^ to 3.2 × 10^−38^, depending on the dataset; MLV, *p =* 2.4 × 10^−22^; ASLV, *p =* 3.2 × 10^−9^) and a stronger association with transcriptional intensity (HIV, *p =* 3.8 × 10^−19^ to 9.7 × 10^−46^; MLV, *p =* 5.1 × 10^−35^; ASLV, *p =* 1.7 × 10^−11^). Overall, ASLV showed the weakest association with gene density and transcriptional intensity.

Thus, the analysis of transcriptional activity in the context of chromosomal location revealed significant effects of transcription on MLV and ASLV integration. This is in contrast to the study based on transcriptional profiling alone, in which the effect was not statistically significant—however, a similar trend was evident and the general conclusions similar (see Figure 3). It appears that adding information on chromosomal position to the gene expression data allowed quantitatively modest effects to reach statistical significance.

### Substructures within Chromosomal Regions Favorable for Integration

Two lines of evidence indicated that the chromosomal regions favorable for integration can be subdivided into favorable and unfavorable segments. In the first study, a computational analysis was carried out to determine the length of the chromosomal segments yielding the best fit between transcriptional intensity and integration intensity. The sizes of the chromosomal regions analyzed were varied systematically from 25 kb to 32,000 kb, and the statistical significance determined for the correlations. This analysis revealed that the segment length yielding the best correlation was comparatively short, around 100–250 kb, the length of one or a few human genes. These conclusions held for HIV, ASLV, and MLV (Protocol S1, p. 16–65).

**Figure 6:**
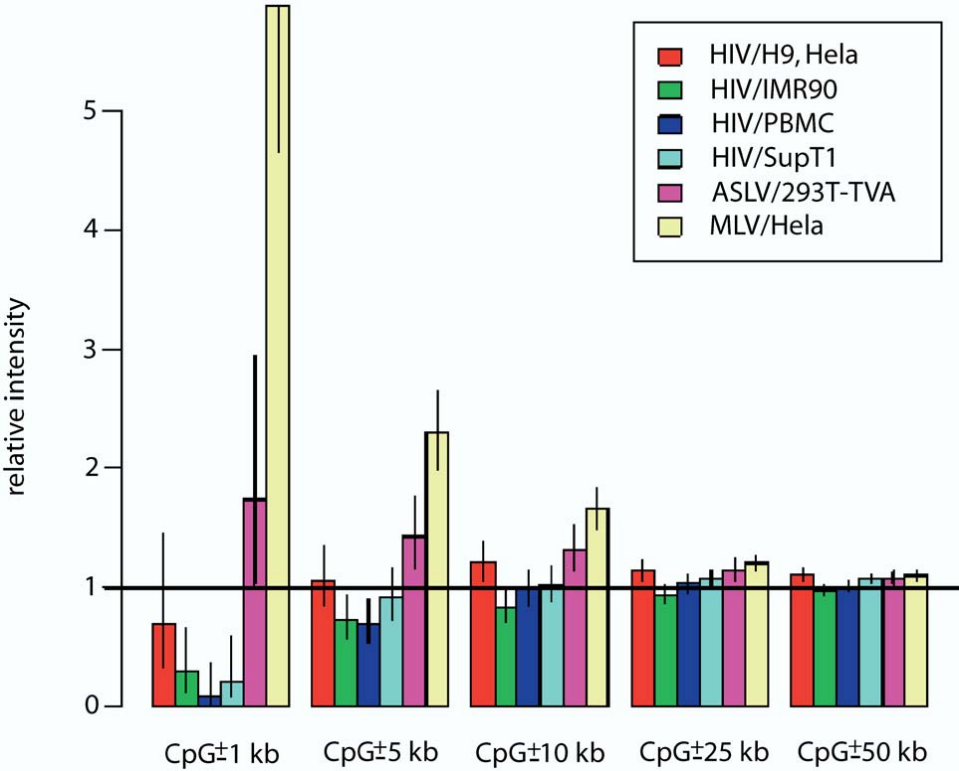
The Effects of Proximity to CpG Islands Differs for HIV, MLV, and ASLV Integration The viral vectors and cell types studied are indicated by color. A value of one indicates no bias, less than one indicates disfavored integration, and more than one indicates favored integration. The x-axis (from plus or minus 1 kb to 50 kb) indicates distance from the edge of a CpG island in either direction along the genome. The statistical analysis specifically removed the favorable effects of being in a gene and being in a region containing expressed genes to highlight the effects of CpG islands alone. When effects of gene density and activity are left in, HIV integration goes from disfavored at short distances (less than 1 kb) to favored at longer distances (more than 10 kb). This is because at longer distances the association with genes is significant—many CpG islands are within 10 kb of a gene, and genes are favored targets for HIV integration. To carry out this analysis, the numbers of experimentally determined and matched control sites were fitted according to whether they were near a CpG island, whether they were in genes, and the level of the expression density variable. Each variable contributes a “multiplier” for the ratio of the number of experimental to control sites. The multiplier for “near CpG island” is shown (see Protocol S2, p. 9–12).

An analysis of integration frequency near CpG islands also indicated substructure within regions favorable for integration. CpG islands are chromosomal regions enriched in the rare CpG dinucleotide. These regions commonly correspond to gene regulatory regions containing clustered transcription factor binding sites—consequently, CpG islands are more frequent in gene-rich regions. Previously Wu et al. (2003) reported that CpG islands were positively associated with MLV integration sites but that for HIV integration sites there was no influence. An analysis of the effects of proximity to CpG islands on integration frequency incorporating the matched random control dataset is shown in Figure 6. The relative integration frequency near CpG islands was found to be much higher than expected by chance for MLV, as reported previously, and slightly higher than expected by chance for ASLV. For HIV, the region surrounding CpG islands was actually disfavored, and this was statistically significant in three out of four datasets. Thus for HIV, broadly favorable gene-dense chromosomal regions actually contain a mixture of favorable clusters of active genes and unfavorable CpG islands. For MLV, in contrast, CpG islands are quite favorable.

High gene density in the human chromosomes is known to correlate with several other features, including high levels of gene expression, high densities of CpG islands, the occurrence of Giemsa-light chromosomal bands, and high G/C content (Caron et al. 2001; Lander et al. 2001; Venter et al. 2001; Mungall et al. 2003; Versteeg et al. 2003). The effects of chromosomal banding pattern and G/C content were analyzed statistically and found to favor integration, as expected from the correlation with other favorable features (Protocol S2).

### A Quantitative Model for Integration Intensity

A statistical model was constructed to examine the relative contributions to integration intensity of (1) gene density, (2) gene activity, (3) proximity to CpG islands, (4) G/C content, and (5) location within genes (Protocol S2, p. 13–15). Inclusion of each of these parameters improved the fit of the model to the observed experimental datasets, but the quantitative contribution of each parameter varied among the different retrovirus types. The effects of being in a gene or a region with many expressed genes were most important for HIV and ASLV. For MLV, the distance from the transcription start site was the most important parameter. ASLV differed significantly from each of the other datasets (*p <* 0.0001), and the model based on the above parameters predicted the placement of ASLV integration sites least well. Thus ASLV is least responsive to the effects of the parameters so far known to affect integration site selection in human cells.

### Integration Frequency in the Individual Human Chromosomes

**Figure 7:**
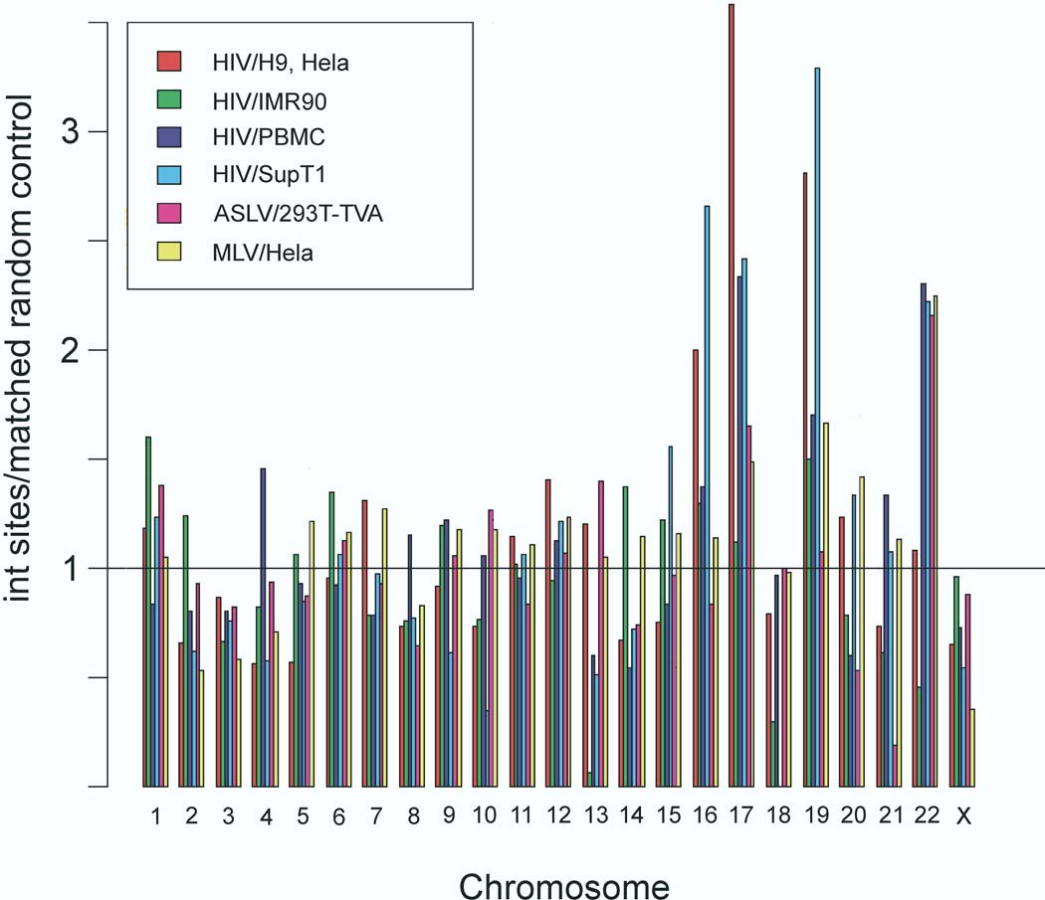
Frequency of Integration in Human Chromosomes Human chromosome numbers are indicated at the bottom of the figure. The number of integration events detected in each chromosome was divided by the number expected from the matched random control. The line at one indicates the bar height expected if the observed number of integration events matched the expected number. Higher bars indicate favored integration, lower bars, disfavored integration. Most of the cell types studied were from human females; too little data were available for the Y chromosome for meaningful analysis.

Figure 7 presents the frequency of integration in each of the different human chromosomes for HIV, MLV, and ASLV. For HIV, each of the datasets is shown separately. A statistical analysis was carried out comparing the observed frequency of integration in each chromosome to that expected from the matched random control (Protocol S1, p. 3). As can be seen from the figure, the frequencies were quite different among the different chromosomes. For example, the gene-rich Chromosome 19 showed more integration than expected by chance, while the gene-poor Chromosome 18 showed less integration. The differences in integration frequencies among chromosomes are in part a function of gene density. However, for unknown reasons, the datasets also differed significantly from each other (*p* < 2.22 × 10^−16^). Evidently there are factors—so far unknown—affecting integration targeting that operate at the level of whole chromosomes(a conclusion also reached by Laufs et al. [2003]).

## Discussion

We report that ASLV, MLV, and HIV have quite different preferences for integration sites in the human chromosomes. HIV strongly favors active genes in primary cells as well as in transformed cell lines. MLV favors integration near transcription start regions and favors active genes only weakly. ASLV shows the weakest bias toward integration in active genes and no favoring of integration near transcription start sites. We expect that these same patterns will be seen for MLV and ASLV integration in different human cell types, because all four HIV datasets yielded similar results, though more data on additional cell and tissue types will be helpful to further evaluate the generality.

One of the earliest models for chromosomal influences on integration targeting proposed that condensed chromatin in inactive regions disfavored integration, thereby concentrating integration in more open active chromatin (Panet and Cedar 1977; Vijaya et al. 1986; Rohdewohld et al. 1987). Integration by HIV, ASLV, and MLV all showed at least a weak bias in favor of integration in active genes, consistent with the idea that open chromatin at active genes favors integration. Also consistent with this idea, heterochromatic regions at human centromeres and telomeres were found to disfavor integration.

However, it seems unlikely that relative accessibility is the only feature directing integration site selection, because HIV, ASLV, and MLV each show such distinctive target sequence preferences. Studies of the Ty retrotransposons of yeast, close relatives of retroviruses, have revealed that interactions with bound chromosomal proteins can tether the Ty integration machinery to chromosomes and thereby direct integration to nearby sites (Boeke and Devine 1998; Bushman 2003; Sandmeyer 2003; Zhu et al. 2003). Such a model may explain integration targeting by retroviruses as well (Bushman 2003). HIV integration complexes might bind to factors enriched at active genes, while MLV complexes could bind to factors bound near transcription start sites. In support of this idea, in vitro studies have established that retroviral integrase enzymes fused to sequence-specific DNA-binding domains can direct integration preferentially to local regions when tethered at specific DNA sites (Bushman 1994; Goulaouic and Chow 1996; Katz et al. 1996).

The analysis of chromosomal regions favored for integration also suggested a role for locally bound proteins. Chromosomal regions enriched in active genes were generally favorable, but further analysis revealed interleaved favorable and unfavorable regions. Statistical tests indicated that favorable regions were typically short (100–250 kb), and for HIV these were interspersed with unfavorable regions near CpG islands. CpG islands are thought to be regulatory regions that bind distinctive sets of transcription factors. Thus, a simple model to explain targeting is that a distinctive set of sequence-specific DNA-binding proteins bound at or near CpG islands disfavor HIV integration, while proteins bound in active transcription units are favorable. For MLV, the proteins bound at CpG islands instead favor integration.

For ASLV, it is possible that the viral integration machinery does not interact with factors bound in or near genes, explaining the more random distribution of integration sites in the genome. Such a pattern might have evolved to minimize disruption to the host cell chromosomes due to integration. Another possibility, however, is that ASLV does have stricter target site preferences during normal integration in chicken cells, but the targeting system does not function properly in the human cells studied here. According to this idea, putative chicken chromosomal proteins normally bind ASLV integration complexes and direct integration, but the homologous human proteins may be too different to interact properly. It should be possible to investigate this possibility by characterizing ASLV integration in chicken cells, now that the draft chicken genome sequence is completed (Ren et al. 2003).

One consequence of the above findings is that integration will differ from tissue to tissue as a consequence of cell-type-specific transcription. To assess effects of tissue-specific transcription, we analyzed HIV integration in three different cell types (SupT1, PBMC, and IMR90). Transcriptional profiling data showed that transcription was significantly different among the three. This allowed an analysis of integration targeting, which showed that highly expressed genes particular to each tissue were favored for integration in that tissue. However, the magnitude of the tissue-specific biases on integration were modest, probably because most of the cellular transcriptional program appears to be common among cell types (Caron et al. 2001; Mungall et al. 2003; Versteeg et al. 2003).

Additional mechanisms could also contribute to targeting. For example, we and others have detected statistically significant biases in integration frequency in whole chromosomes that do not appear to be fully explained by gene density or gene activity (Schroder et al. 2002; Laufs et al. 2003; data reported here). Perhaps the intranuclear position of chromosomes may have an influence, since this has been proposed to be relatively fixed for cells of specific types but may differ among cell types (Boyle et al. 2001; Chubb and Bickmore 2003).

Our data indicate that ASLV has integration site preferences that may make it attractive as a vector for human gene therapy. MLV-based vectors have the unfavorable preference for integration near transcription start sites (Wu et al. 2003). The adverse events arising during X-linked severe combined immunodeficiency gene therapy involved integration of an MLV vector near the transcription start region of the LMO2 proto-oncogene. HIV-based vectors strongly favor integration in active genes, which is also likely to be disruptive to the host cell genome (Schroder et al. 2002; Wu et al. 2003). ASLV, in contrast, shows only weak favoring of integration in active genes, and no favoring of integration near transcription start sites. A quantitative model based on gene density, expression, and proximity to transcription start regions fit the ASLV data least well, indicating that ASLV has the weakest bias toward integration in these unfavorable locations. ASLV vectors are known to infect a variety of human cell types (e. g., Valsesia-Wittmann et al. 1994; Federspiel and Hughes 1997; Hatziioannou and Goff 2001; Katz et al. 2002) and can transduce nondividing cells (Hatziioannou and Goff 2001; Katz et al. 2002), adding to their possible utility. More generally, this study, together with previous work (Schroder et al. 2002; Wu et al. 2003), indicates that the selection of different retroviral integration systems can modulate the selection of integration target sites, and this may potentially be exploited for safer gene therapy.

## Materials and Methods

### 

#### Oligonucleotides used in this study

Each oligonucleotide is described by its name, sequence (written 5′ to 3′), and use, in that order. *Hinc*II adaptor, GTAATACGACTCACTATAGGGCACGCGTGGTCGACGGCCCGGGCTGC, adapter for use with DNA cleaved by 6-cutter restriction enzymes, top strand; mNheIAvrIISpeII adaptor, P-CTAGGCAGCCCG-NH_2_, adapter for 6-cutter restriction enzymes, bottom strand; ASB-9, GACTCACTATAGGGCACGCGT, adapter primer for PCR for I/SupT1, PBMC, and IMR-90; SB-76, GAGGGATCTCTAGTTACCAGAGTCACA, HIV primer for PCR for I/SupT1; ASB-19, GAGATTTTCCACACTGACTAAAAGGGTC, HIV primer for PCR for I/PBMC and IMR-90; ASB-16, GTCGACGGCCCGGGCTGCCTA, adapter primer for nested PCR for I/SupT1, PBMC, and IMR-90; ASB-1, AGCCAGAGAGCTCCCAGGCTCAGATC, HIV primer for nested PCR for I/SupT1; ASB-20, CTGAGGGATCTCTAGTTACCAGAGTCA, HIV primer for nested PCR forPBMC and IMR-90; MseI linker +, GTAATACGACTCACTATAGGGCTCCGCTTAAGGGAC, MseI linker, top strand; MseI linker −, P-TAGTCCCTTAAGCGGAG-NH_2,_ MseI linker, bottom strand; MseI linker primer, GTAATACGACTCACTATAGGGC, MseI linker primer for first round of PCR; MseI linker nested primer, AGGGCTCCGCTTAAGGGAC, MseI linker nested primer for second round of PCR; BB389, GATGGCCGGACCGTTGATTC, inner ASLV LTR primer for second round of “nested” PCR; BB390, CGATACAATAAACGCCATTTGACCATTC, outer ASLV LTR primer for first round of PCR.

#### Preparation of the ASLV- and HIV-based vectors

To produce HIV vector particles, 293T cells were cotransfected with three plasmids: one encoding the HIV vector segment (p156RRLsinPPTCMVGFPWPRE) (Follenzi et al. 2000), the second, the packaging construct (pCMVdeltaR9) (Naldini et al. 1996), and the third, the gene for *VSV-G* (pMD.G) (Naldini et al. 1996). Forty-eight hours after transfection, supernatants were collected, centrifuged to pellet cellular debris, then filtered through 0.45-μm filters. Viral particles were further purified by centrifugation at 23,000g and resuspended in 1/17 volume of fresh medium. ASLV particles were generated by transfecting the DF-1 chicken embryonic fibroblast cell line (ATCC CRL-12203) with the plasmid RCASBP(A)GFP (from Steve Hughes, National Cancer Institute, Frederick, Maryland). Supernatant was removed from the cells 4 d post transfection (when cells were nearly 100% GFP positive) and filtered though a 0.45-μm syringe filter.

#### Infections.

PBMCs were separated from human blood using a ficoll gradient (Amersham Biosciences, Little Chalfont, United Kingdom). 1 × 10^7^ PHA, IL-2 prestimulated PBMCs, or IMR-90 cells (passage #36) at 30%–50% confluency (1–2 × 10^6^ cells) were infected with the HIV-based vector at an moi of 10 (60 ng p24 per 5 × 10 ^5^ cells). The vector was added to the cells with DEAE-dextran at a final concentration of 5 μg/ml. Forty-eight hours after infection, the cells were pelleted. For RNA isolation, cells were resuspended in 250 μl of PBS and 750 μl of TRIzol and frozen in liquid nitrogen. To determine the extent of infection, cells were analyzed by flow cytometry. For ASLV, supernatant containing RCASBP(A)GFP particles was added to 293T-TVA cells (293T 0.8 cells; a gift from John Young, Salk Institute) at 30%–50% confluency. Forty-eight hours post infection, green fluorescence was seen in approximately 30% of the cells, as determined by examination of the cultures with a fluorescence microscope. DNA was harvested at this point (DNeasy, Qiagen, Valencia, California, United States). RNA from infected cells was also isolated at 48 h post infection (TRIzol) and stored at −80 °C until used for transcriptional profiling analysis. RNA was isolated from infected cell cultures, and samples from each were used for hybridization on one Affymetrix (Santa Clara, California, United States) microarray.

#### Integration site determination.

HIV integration sites were cloned by ligation-mediated PCR essentially as described in Schroder et al. (2002). ASLV integration site determination using ligation-mediated PCR was carried out essentially as described in Wu et al. (2003). Oligonucleotides used are summarized above. All novel integration site sequences are deposited at the National Center for Biotechnology Information (NCBI) (accession numbers CL528318–CL529767). Integration sites from earlier studies were reanalyzed on the November 2002 freeze of the human genome sequence (using the University of California at Santa Cruz browser), and a few were excluded because they did not find matches of sufficiently high quality on the new draft sequence, accounting for slightly different numbers than in previous reports. This study used primarily the Acembly and Ensemble human gene catalogs; similar results were generally obtained when the Unigene, RefGene, or GeneScan catalogs of the human genes were used (Protocol S1, p. 3–10).

#### Transcriptional profiling analysis

Transcriptional profiling was carried out using Affymetrix microarrays as described in Schroder et al. (2002). Gene expression levels (average difference values) were analyzed using Affymetrix Microarray Suite 4.1 software. All novel transcriptional profiling datasets reported here are deposited at NCBI (GEO dataset numbers GSE1407, GSE1408, GSE1409, and GSE1410). For the analysis in Figure 3, a complication was introduced by the fact that the HU95A chips used have multiple probe sets for some genes but not others. In our analysis all probe sets were accepted and analyzed in these cases.

#### Statistical analysis

A detailed description of the statistical methods used is presented in Protocols S1 and S2.

## Supporting Information

Protocol S1 Association of Genomic Features with Integration—Part 1 (322 KB PDF).

Protocol S2 Association of Genomic Features with Integration—Part 2 (128 KB PDF).

### Accession Numbers

The GenBank (http://www.ncbi.nlm.nih.gov/) accession numbers for the novel integration site sequences discussed in this paper are CL528318–CL529767. The GenBank GEO dataset numbers for the novel transcriptional profiling datasets reported here are GSE1407, GSE1408, GSE1409, and GSE1410.

## Abbreviations

ASLV: avian sarcoma-leukosis virus
HIV: human immunodeficiency virus
MLV: murine leukemia virus
PBMC: peripheral blood mononuclear cell
