# Supplementary material for: Retroviral DNA Integration: ASLV, HIV, and MLV Show Distinct Target Site Preferences
Source: PLoS Biol. 2004 Aug 17;2(8):e234. doi: 10.1371/journal.pbio.0020234 (PMC509299; doi:10.1371/journal.pbio.0020234)
Supplement: Protocol S1 — (322 KB PDF). [file pbio.0020234.sd001.pdf]

# Association of Genomic Features with Integration

Charles C. Berry

April 19, 2004

## Contents

**1 Introduction** **2**

**2 Preference for Genes** **3**
2.1 Acembly Genes . . . . . . . . . . 3
2.2 refGenes . . . . . . . . . . 6
2.3 genScan Genes . . . . . . . . . . 7
2.4 uniGenes . . . . . . . . . . 9

**3 CpG Island Neighborhoods** **11**
3.1 1 kilobase neighborhoods . . . . . . . . . . 12
3.2 5 kilobase neighborhoods . . . . . . . . . . 12
3.3 10 kilobase neighborhoods . . . . . . . . . . 13
3.4 25 kilobase neighborhoods . . . . . . . . . . 14
3.5 50 kilobase neighborhoods . . . . . . . . . . 15

**4 Gene Density, Expression 'Density', and CpG Island Density** **16**
4.1 25 kiloBase Window . . . . . . . . . . 17
4.2 50 kiloBase Window . . . . . . . . . . 22
4.3 100 kiloBase Window . . . . . . . . . . 27
4.4 250 kiloBase Window . . . . . . . . . . 32
4.5 500 kiloBase Window . . . . . . . . . . 37
4.6 1 megaBase Window . . . . . . . . . . 42
4.7 2 megaBase Window . . . . . . . . . . 47
4.8 4 megaBase Window . . . . . . . . . . 52
4.9 4 megaBase Window . . . . . . . . . . 57
4.10 16 megaBase Window . . . . . . . . . . 61
4.11 32 megaBase Window . . . . . . . . . . 65

**5 Juxtaposition with Gene Start and End Positions** **69**
5.1 Acembly Annotations . . . . . . . . . . 69
5.2 RefSeq Annotations . . . . . . . . . . 74
5.3 genScan Annotations . . . . . . . . . . 78
5.4 uniGene Annotations . . . . . . . . . . 82

**6 GC content** 85

**7 Cytobands** 86

# 1 Introduction

In this document, I examine the association of integration siting with various genomic features.

The data consist of both actual integration sites and sets of control sites, each set chosen to match the spacing (in bases) from the nearest restriction site (according to the direction in which the sequence was read) to an integration site. The numbers of insertion and matching sites for several data sets are shown below:

```
                  type
Origin.of.data.set insertion match
     HIV/H9, Hela   407       4070
     HIV/IMR90      465       4650
     HIV/PBMC       528       5280
     HIV/SupT1      436       4360
     ASLV/293T-TVA  469       4690
     MLV/Hela       822       8220
```

The advantage of choosing 'control' sites that match the spacing from the nearest restriction site is that biases due to location and density of restriction sites are eliminated by applying the classical multinomial logit model (reviewed in [2]). This model allows regression procedures to be applied to the study of integration intensity as a function of genomic features. The `clogit` function of the R `survival` library) implements estimation and fitting for such models along with the usual likelihood ratio and Wald tests.

The distribution of relative frequency of insertions across the chromosomes is given in this barplot:

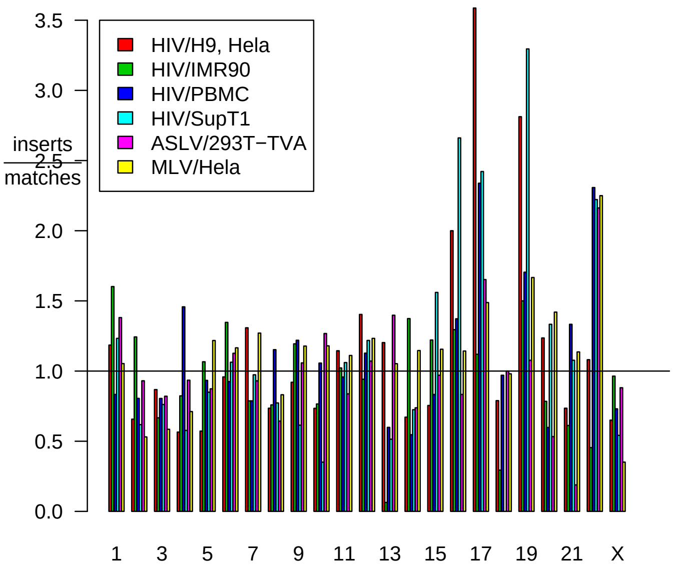


It seems evident that there are some chromosomes that are particularly favored for integration. This is reinforced by a test of statistical significance. The test performed used the likelihood ratio statistic for the multinomial logit model (reviewed in [2]) as implemented by the `clogit` function of the R `survival` library). The null hypothesis tested is the ratio of true integration events to matched control sites is constant across all crhomosomes. This test attains a p-value of $< 2.22e - 16$.

# 2 Preference for Genes

## 2.1 Acembly Genes

Here we examine the preference that integration events have for genes. In the following plot we show the relative frequency of integrations in genes according to the 'Acembly' anotation. The bars grouped over the label "In Gene" give the relative frequency of integration events (compared to control sites) between bases located within Acembly gene annotations, while the label "Not in Gene" give the relative frequency of integration events (compared to control sites) between bases not located within Acembly gene annotations.

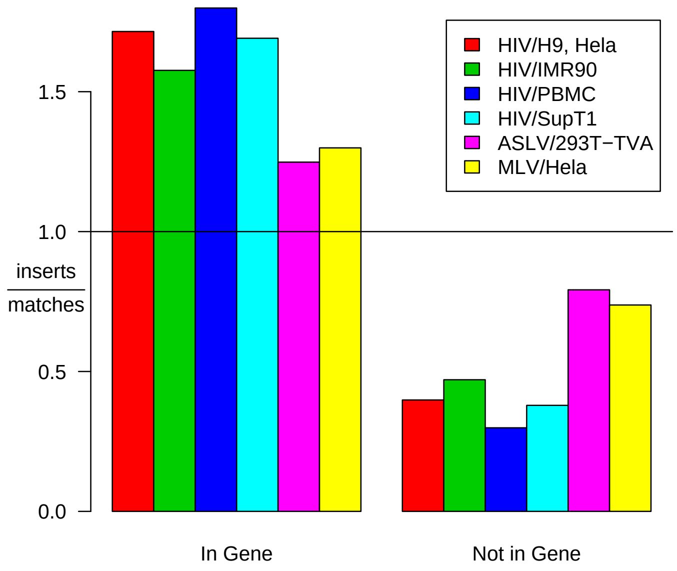


It seems evident that there is a strong tendency for insertions to occur in genes. A formal test of significance bears this out wiht a p-value of $< 2.22e-16$. Also, it appears that the tendency of genes in cells of different origin to attract insertions varies, and a test for this hypothesis attains $< 2.22e-16$. Here is the table of coefficients of the log ratio of intensities for true insertion sites versus control insertion sites along with their standard errors, z statistics, and p-values according to origin of the cells:

| | coef | se | z | p |
|---|---|---|---|---|
| HIV/H9, Hela | 1.450 | 0.1250 | 11.70 | 1.77e-31 |
| HIV/IMR90 | 1.210 | 0.1120 | 10.80 | 3.80e-27 |
| HIV/PBMC | 1.810 | 0.1230 | 14.60 | 1.45e-48 |
| HIV/SupT1 | 1.490 | 0.1240 | 12.10 | 1.65e-33 |
| ASLV/293T-TVA | 0.457 | 0.0980 | 4.66 | 3.12e-06 |
| MLV/Hela | 0.568 | 0.0749 | 7.58 | 3.45e-14 |

As is evident, there are some differences in the coefficients. The largest coefficient is seen in the HIV/PBMC data set, while the smallest is seen in the ASLV/293T-TVA data set.

In the following plot we show the relative frequency of insertions in exons according to the 'Acembly' anotation The bars grouped over the label "In Exon" give the relative frequency of integration events (compared to control sites) between bases located in exons according to the Acembly annotation, while the

label “Not in Exon” give the relative frequency of integration events (compared to control sites) between bases not located in exons according to the Acembly gene annotation.

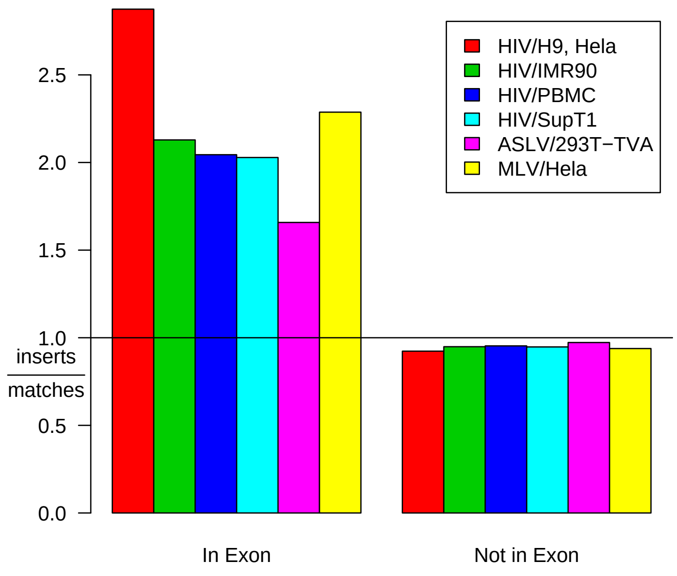


Here is the table of coefficients of the log ratio of intensities for true insertion sites versus control insertion sites along with their standard errors, z statistics, and p-values according to origin of the cells:

```
               coef    se     z        p
HIV/H9, Hela  0.579 0.181 3.210 1.33e-03
HIV/IMR90     0.350 0.178 1.970 4.92e-02
HIV/PBMC      0.124 0.172 0.723 4.70e-01
HIV/SupT1     0.206 0.179 1.150 2.49e-01
ASLV/293T-TVA 0.312 0.205 1.520 1.28e-01
MLV/Hela      0.650 0.130 4.980 6.38e-07
```

The model on which these coefficients are based include terms for whether the site is in a gene or not. Thus, the effect shown in net of that due to being in a gene. Note that in the barplot above the ’Not in Exon’ bars include the both introns and intergenic regions, so the impression given by the table may differ from that for the barplot.

## 2.2 refGenes

Here we examine the preference that insertions have for genes. In the following plot we show the relative frequency of insertions in genes according to the 'refGene' anotation.

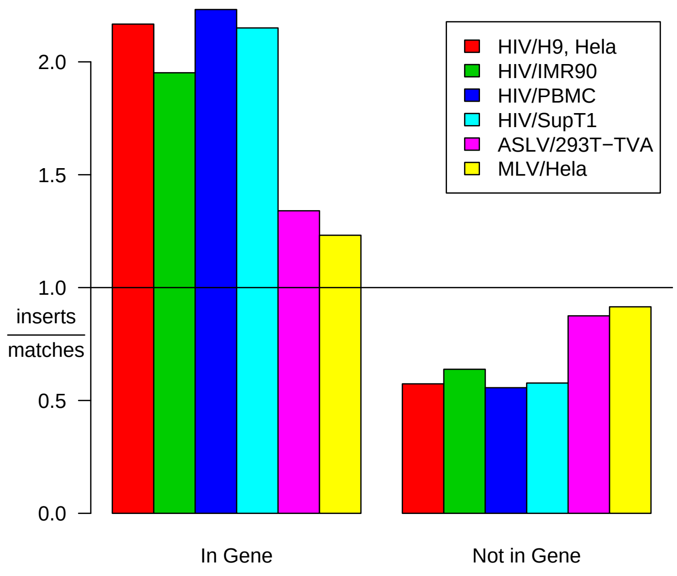


It seems evident that there is a strong tendency for insertions to occur in genes. A formal test of significance bears this out wiht a p-value of $< 2.22e-16$. Also, it appears that the tendency of genes in cells of different origin to attract insertions varies, and a test for this hypothesis attains $< 2.22e-16$. Here is the table of coefficients of the log ratio of intensities for true insertion sites versus control insertion sites along with their standard errors, z statistics, and p-values according to origin of the cells:

| | coef | se | z | p |
|---|---|---|---|---|
| HIV/H9, Hela | 1.330 | 0.1070 | 12.40 | 3.77e-35 |
| HIV/IMR90 | 1.120 | 0.0991 | 11.30 | 1.02e-29 |
| HIV/PBMC | 1.390 | 0.0947 | 14.70 | 7.54e-49 |
| HIV/SupT1 | 1.320 | 0.1030 | 12.80 | 2.64e-37 |
| ASLV/293T-TVA | 0.430 | 0.1020 | 4.21 | 2.55e-05 |
| MLV/Hela | 0.297 | 0.0782 | 3.81 | 1.42e-04 |

As is evident, there are some differences in the coefficients. The largest coefficient is seen in the HIV/PBMC data set, while the smallest is seen in the MLV/Hela data set.

In the following plot we show the relative frequency of insertions in exons according to the 'refGene' anotation.

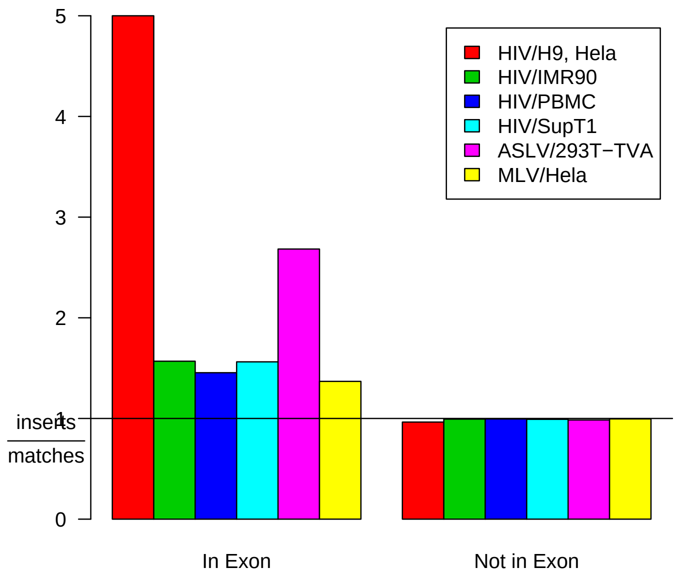


Here is the table of coefficients of the log ratio of intensities for true insertion sites versus control insertion sites along with their standard errors, z statistics, and p-values according to origin of the cells:

| | coef | se | z | p |
|---|---|---|---|---|
| HIV/H9, Hela | 0.853 | 0.299 | 2.850 | 0.00435 |
| HIV/IMR90 | -0.239 | 0.387 | -0.618 | 0.53700 |
| HIV/PBMC | -0.503 | 0.384 | -1.310 | 0.19100 |
| HIV/SupT1 | -0.346 | 0.348 | -0.992 | 0.32100 |
| ASLV/293T-TVA | 0.729 | 0.347 | 2.100 | 0.03590 |
| MLV/Hela | 0.102 | 0.304 | 0.337 | 0.73600 |

The model on which these coefficients are based include terms for whether the site is in a gene or not. Thus, the effect shown in net of that due to being in a gene.

## 2.3 genScan Genes

Here we examine the preference that insertions have for genes. In the following plot we show the relative frequency of insertions in genes according to the 'genScan' anotation.

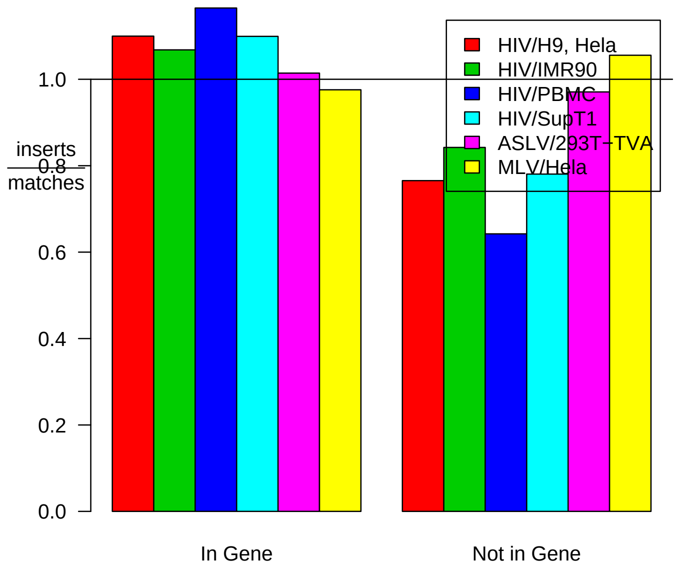


It seems evident that there is a strong tendency for insertions to occur in genes. A formal test of significance bears this out wiht a p-value of $1.0137e-06$. Also, it appears that the tendency of genes in cells of different origin to attract insertions varies, and a test for this hypothesis attains $9.0875e-06$. Here is the table of coefficients of the log ratio of intensities for true insertion sites versus control insertion sites along with their standard errors, z statistics, and p-values according to origin of the cells:

| | coef | se | z | p |
|---|---|---|---|---|
| HIV/H9, Hela | 0.3600 | 0.1230 | 2.940 | 3.29e-03 |
| HIV/IMR90 | 0.2370 | 0.1110 | 2.130 | 3.29e-02 |
| HIV/PBMC | 0.5950 | 0.1120 | 5.300 | 1.17e-07 |
| HIV/SupT1 | 0.3440 | 0.1170 | 2.950 | 3.20e-03 |
| ASLV/293T-TVA | 0.0439 | 0.1040 | 0.423 | 6.73e-01 |
| MLV/Hela | -0.0789 | 0.0785 | -1.000 | 3.15e-01 |

As is evident, there are some differences in the coefficients. The largest coefficient is seen in the HIV/PBMC data set, while the smallest is seen in the MLV/Hela data set.

In the following plot we show the relative frequency of insertions in exons according to the 'genScan' anotation.

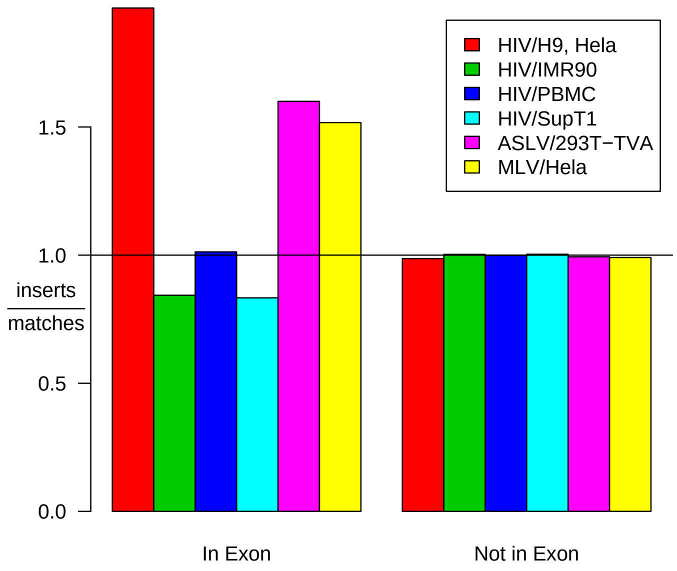


Here is the table of coefficients of the log ratio of intensities for true insertion sites versus control insertion sites along with their standard errors, z statistics, and p-values according to origin of the cells:

| | coef | se | z | p |
|---|---|---|---|---|
| HIV/H9, Hela | 0.598 | 0.336 | 1.780 | 0.0748 |
| HIV/IMR90 | -0.241 | 0.397 | -0.607 | 0.5440 |
| HIV/PBMC | -0.131 | 0.374 | -0.351 | 0.7250 |
| HIV/SupT1 | -0.293 | 0.397 | -0.740 | 0.4590 |
| ASLV/293T-TVA | 0.463 | 0.384 | 1.210 | 0.2280 |
| MLV/Hela | 0.458 | 0.234 | 1.960 | 0.0504 |

The model on which these coefficients are based include terms for whether the site is in a gene or not. Thus, the effect shown in net of that due to being in a gene.

## 2.4 uniGenes

Here we examine the preference that insertions have for genes. In the following plot we show the relative frequency of insertions in genes according to the 'uniGene' anotation.

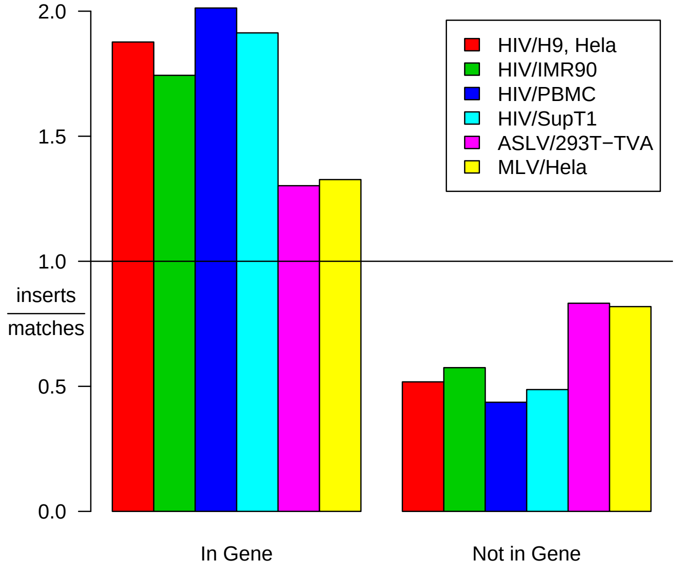


It seems evident that there is a strong tendency for insertions to occur in genes. A formal test of significance bears this out wiht a p-value of $< 2.22e-16$. Also, it appears that the tendency of genes in cells of different origin to attract insertions varies, and a test for this hypothesis attains $< 2.22e-16$. Here is the table of coefficients of the log ratio of intensities for true insertion sites versus control insertion sites along with their standard errors, z statistics, and p-values according to origin of the cells:

| | coef | se | z | p |
|---|---|---|---|---|
| HIV/H9, Hela | 1.290 | 0.1110 | 11.60 | 2.67e-31 |
| HIV/IMR90 | 1.110 | 0.1010 | 11.00 | 5.74e-28 |
| HIV/PBMC | 1.540 | 0.1020 | 15.10 | 1.56e-51 |
| HIV/SupT1 | 1.370 | 0.1080 | 12.60 | 2.02e-36 |
| ASLV/293T-TVA | 0.452 | 0.0981 | 4.61 | 3.99e-06 |
| MLV/Hela | 0.484 | 0.0738 | 6.57 | 5.14e-11 |

As is evident, there are some differences in the coefficients. The largest coefficient is seen in the HIV/PBMC data set, while the smallest is seen in the ASLV/293T-TVA data set.

In the following plot we show the relative frequency of insertions in exons according to the 'uniGene' anotation.

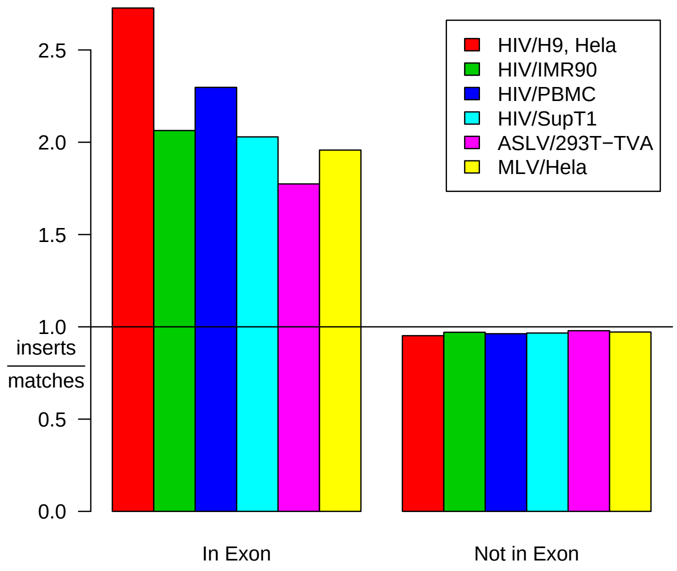


Here is the table of coefficients of the log ratio of intensities for true insertion sites versus control insertion sites along with their standard errors, z statistics, and p-values according to origin of the cells:

| | coef | se | z | p |
|---|---|---|---|---|
| HIV/H9, Hela | 0.4190 | 0.217 | 1.930 | 0.0534 |
| HIV/IMR90 | 0.1880 | 0.225 | 0.833 | 0.4050 |
| HIV/PBMC | 0.1240 | 0.200 | 0.617 | 0.5370 |
| HIV/SupT1 | 0.0867 | 0.219 | 0.395 | 0.6930 |
| ASLV/293T-TVA | 0.3390 | 0.243 | 1.390 | 0.1640 |
| MLV/Hela | 0.4360 | 0.173 | 2.520 | 0.0116 |

The model on which these coefficients are based include terms for whether the site is in a gene or not. Thus, the effect shown in net of that due to being in a gene.

# 3 CpG Island Neighborhoods

Here we study the effect of being in the neighborhood of CpG Islands. Following Wu et al [4], who found that the neighborhoods within ±1kb of CpG islands are enriched for MLV insertions, we study such neighborhoods.

## 3.1 1 kilobase neighborhoods

The following plot shows the effect of being in or within ±1kb of a CpG island:

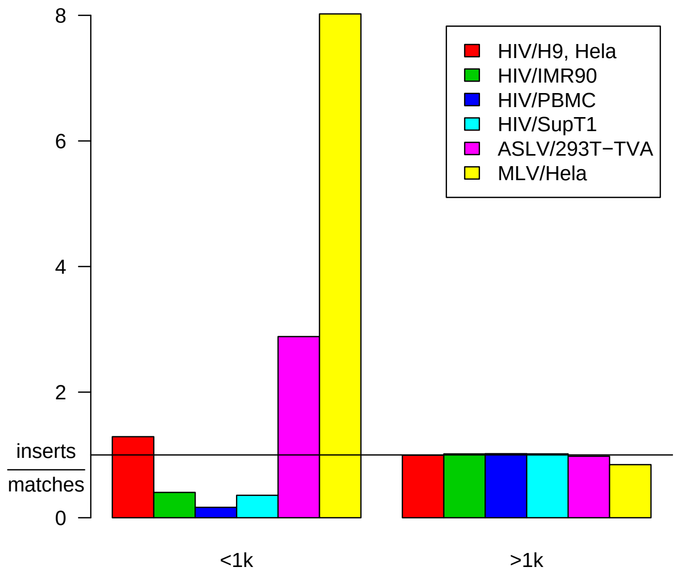


A formal test of significance comparing the difference attains a p-value of $< 2.22e-16$. A test for differences in between cell lines attains $< 2.22e-16$. Here is the table of coefficients of the log ratio of intensities for true insertion sites versus control insertion sites along with their standard errors, z statistics, and p-values according to origin of the cells:

| | coef | se | z | p |
|---|---|---|---|---|
| HIV/H9, Hela | 0.259 | 0.379 | 0.683 | 4.94e-01 |
| HIV/IMR90 | -0.929 | 0.459 | -2.020 | 4.32e-02 |
| HIV/PBMC | -1.820 | 0.715 | -2.550 | 1.07e-02 |
| HIV/SupT1 | -1.050 | 0.511 | -2.050 | 4.07e-02 |
| ASLV/293T-TVA | 1.100 | 0.301 | 3.650 | 2.66e-04 |
| MLV/Hela | 2.260 | 0.122 | 18.500 | 2.45e-76 |

The largest coefficient is seen in the MLV/Hela data set, while the smallest is seen in the HIV/PBMC data set.

## 3.2 5 kilobase neighborhoods

The following plot shows the effect of being in or within ±5kb of a CpG island:

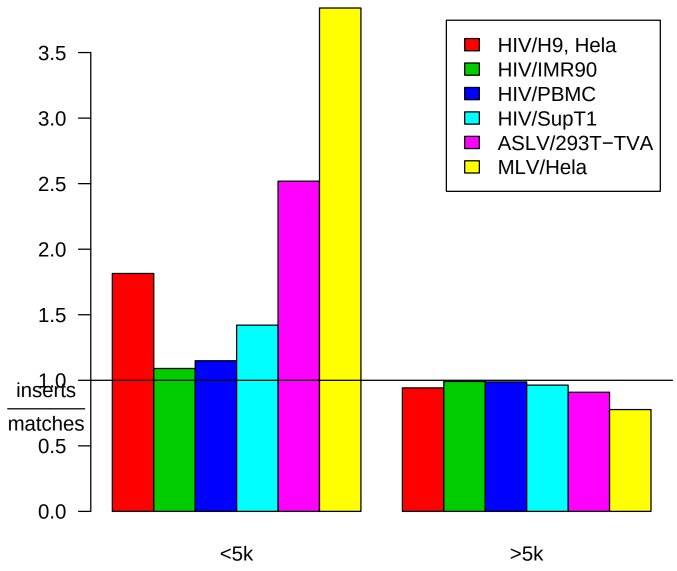


A formal test of significance comparing the difference attains a p-value of $< 2.22e - 16$. A test for differences in between cell lines attains $< 2.22e - 16$. Here is the table of coefficients of the log ratio of intensities for true insertion sites versus control insertion sites along with their standard errors, z statistics, and p-values according to origin of the cells:

| | coef | se | z | p |
|---|---|---|---|---|
| HIV/H9, Hela | 0.6530 | 0.1650 | 3.970 | 7.19e-05 |
| HIV/IMR90 | 0.0955 | 0.1640 | 0.583 | 5.60e-01 |
| HIV/PBMC | 0.1520 | 0.1620 | 0.941 | 3.47e-01 |
| HIV/SupT1 | 0.3880 | 0.1600 | 2.420 | 1.54e-02 |
| ASLV/293T-TVA | 1.0200 | 0.1470 | 6.960 | 3.44e-12 |
| MLV/Hela | 1.6400 | 0.0911 | 18.000 | 1.98e-72 |

The largest coefficient is seen in the MLV/Hela data set, while the smallest is seen in the HIV/IMR90 data set.

## 3.3 10 kilobase neighborhoods

The following plot shows the effect of being in or within ±10kb of a CpG island:

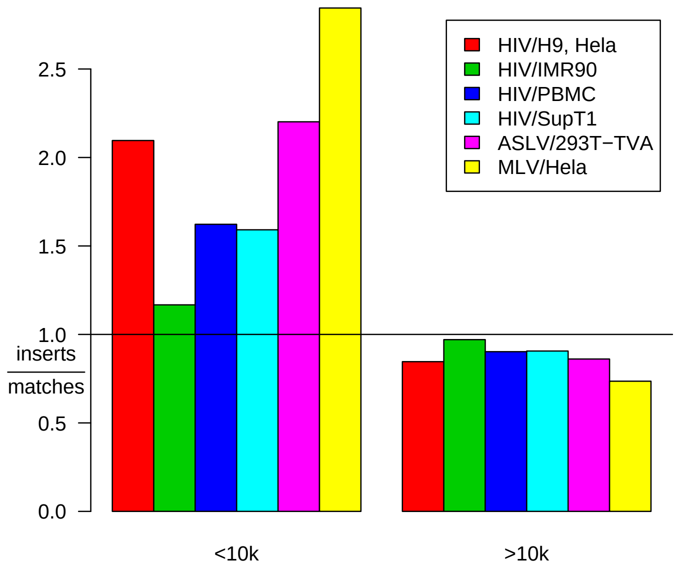


A formal test of significance comparing the difference attains a p-value of $< 2.22e - 16$. A test for differences in between cell lines attains $4.1006e - 16$. Here is the table of coefficients of the log ratio of intensities for true insertion sites versus control insertion sites along with their standard errors, z statistics, and p-values according to origin of the cells:

| | coef | se | z | p |
|---|---|---|---|---|
| HIV/H9, Hela | 0.902 | 0.1230 | 7.34 | 2.17e-13 |
| HIV/IMR90 | 0.186 | 0.1300 | 1.43 | 1.52e-01 |
| HIV/PBMC | 0.592 | 0.1130 | 5.23 | 1.73e-07 |
| HIV/SupT1 | 0.561 | 0.1240 | 4.53 | 5.95e-06 |
| ASLV/293T-TVA | 0.936 | 0.1200 | 7.80 | 6.34e-15 |
| MLV/Hela | 1.380 | 0.0815 | 16.90 | 4.78e-64 |

The largest coefficient is seen in the MLV/Hela data set, while the smallest is seen in the HIV/IMR90 data set.

### 3.4 25 kilobase neighborhoods

The following plot shows the effect of being in or within ±25kb of a CpG island:

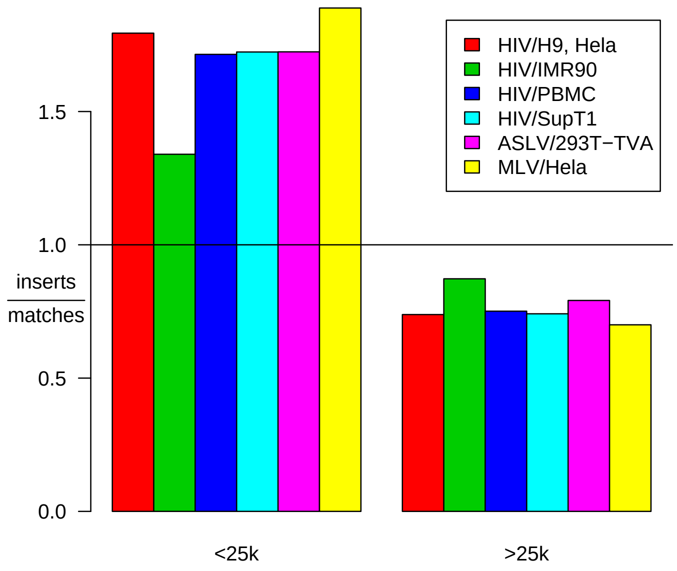


A formal test of significance comparing the difference attains a p-value of $< 2.22e - 16$. A test for differences in between cell lines attains 0.00058116. Here is the table of coefficients of the log ratio of intensities for true insertion sites versus control insertion sites along with their standard errors, z statistics, and p-values according to origin of the cells:

| | coef | se | z | p |
|---|---|---|---|---|
| HIV/H9, Hela | 0.896 | 0.1070 | 8.38 | 5.33e-17 |
| HIV/IMR90 | 0.432 | 0.1020 | 4.22 | 2.42e-05 |
| HIV/PBMC | 0.832 | 0.0937 | 8.88 | 6.76e-19 |
| HIV/SupT1 | 0.830 | 0.1010 | 8.19 | 2.52e-16 |
| ASLV/293T-TVA | 0.784 | 0.1020 | 7.72 | 1.15e-14 |
| MLV/Hela | 1.010 | 0.0753 | 13.40 | 3.93e-41 |

The largest coefficient is seen in the MLV/Hela data set, while the smallest is seen in the HIV/IMR90 data set.

### 3.5 50 kilobase neighborhoods

The following plot shows the effect of being in or within ±50kb of a CpG island:

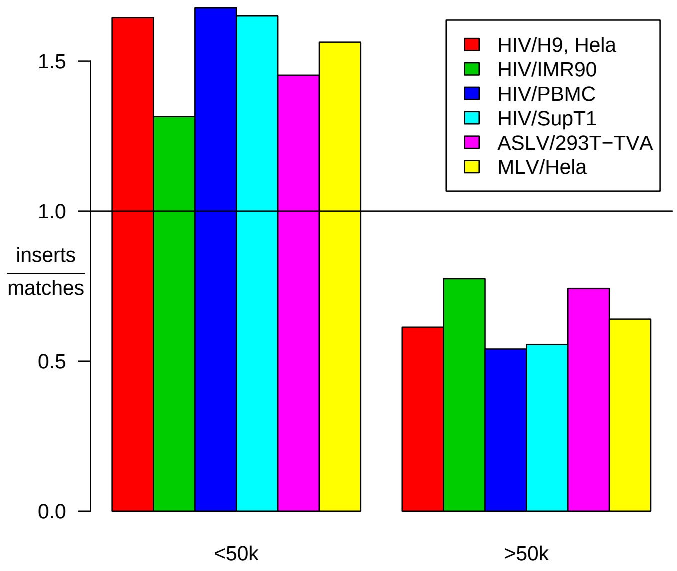


A formal test of significance comparing the difference attains a p-value of $< 2.22e - 16$. A test for differences in between cell lines attains $2.5186e - 05$. Here is the table of coefficients of the log ratio of intensities for true insertion sites versus control insertion sites along with their standard errors, z statistics, and p-values according to origin of the cells:

| | coef | se | z | p |
|---|---|---|---|---|
| HIV/H9, Hela | 0.983 | 0.1070 | 9.19 | 4.09e-20 |
| HIV/IMR90 | 0.530 | 0.0980 | 5.41 | 6.22e-08 |
| HIV/PBMC | 1.140 | 0.0979 | 11.70 | 2.11e-31 |
| HIV/SupT1 | 1.080 | 0.1060 | 10.20 | 2.98e-24 |
| ASLV/293T-TVA | 0.675 | 0.0977 | 6.91 | 4.81e-12 |
| MLV/Hela | 0.912 | 0.0760 | 12.00 | 3.14e-33 |

The largest coefficient is seen in the HIV/PBMC data set, while the smallest is seen in the HIV/IMR90 data set.

# 4 Gene Density, Expression 'Density', and CpG Island Density

In this section the association with gene density is examined. The 'genes' that are counted are the Ensembl genes. In addition, we study various functions of

the EST counts for the Ensembl genes using data described in Versteeg et al [3] and CpG Island density. Based on preliminary observations, it was decided to determine the density of ESTs found in a region in the following ways:

**count.exprs** Count only one EST per gene and divide by number of bases

**exprs** Count up to 200 ESTs per gene and divide by number of bases

**big.exprs** Counting only the ESTs in excess of two hundred per gene and divide by number of bases

The bolded terms are used as abbreviations in what follows. The abbreviation **dens** is used to indicate gene density as number of genes per base.

## 4.1 25 kiloBase Window

In the barplot that follows we examine the association of insertion sites with gene density in a 25 kilobase window surrounding each locus. More such plots will follow and the method of their construction is always to try to divide the data according to the deciles of density. However, it often happens that there is a very skewed distribution of density and often even the $90^{th}$ percentile is zero. In that case, the barplots simply show the sites for which the density is zero and those for which it is non-zero. If there are fewer than ten groups of bars, then the groupings contain ten percent of the sites each except for the leftmost grouping which will contain all of the remaining sites.

Also note that the title of the plot contains clues as to its content; the prefix indicates the type of variable studied while the suffix indicates the window width in the number of bases.

Following the plots is a table of fitted coefficients based on splitting the density data at the median. (If the median is zero, then non-zero values are compared to zero values.)

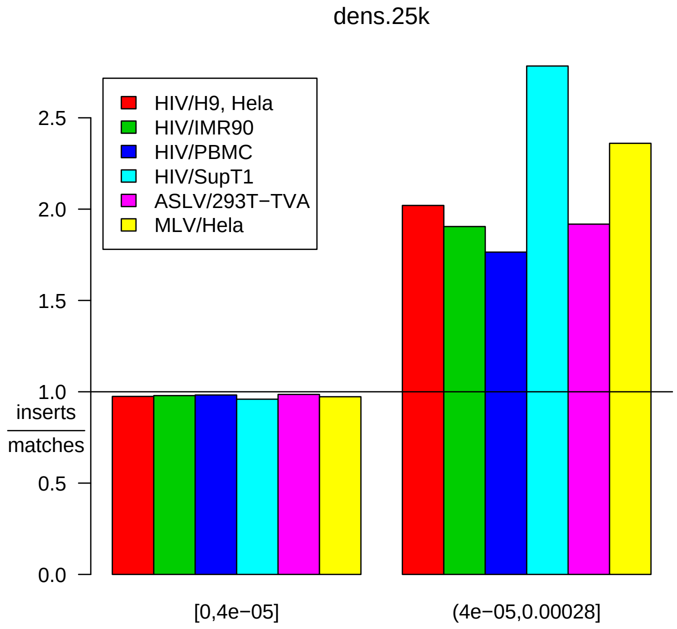


| | coef | se | z | p |
|---|---|---|---|---|
| HIV/H9, Hela | 1.120 | 0.1120 | 10.00 | 1.27e-23 |
| HIV/IMR90 | 0.811 | 0.1050 | 7.72 | 1.18e-14 |
| HIV/PBMC | 1.120 | 0.0961 | 11.70 | 1.14e-31 |
| HIV/SupT1 | 0.973 | 0.1060 | 9.16 | 5.06e-20 |
| ASLV/293T-TVA | 0.485 | 0.1180 | 4.10 | 4.05e-05 |
| MLV/Hela | 0.695 | 0.0830 | 8.37 | 5.70e-17 |

In the barplot that follows we examine the association of insertion sites with expression density in a 25 kilobase window surrounding each locus. First, we count just one EST per gene.

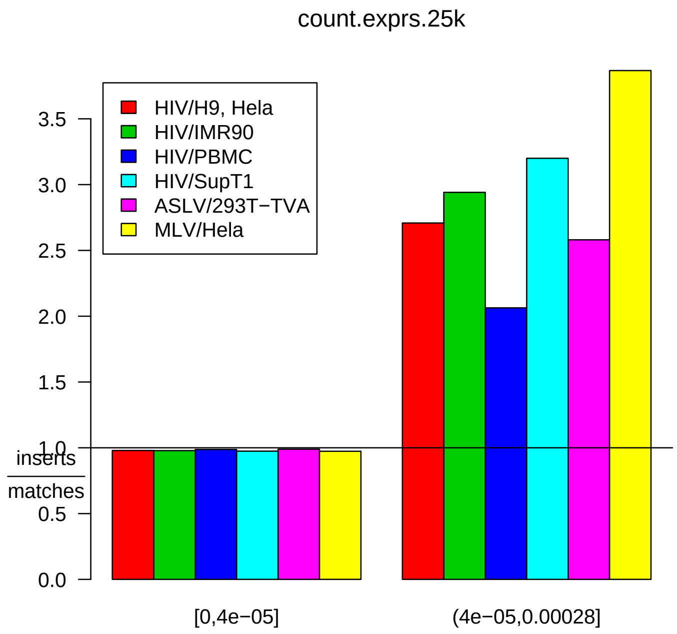


| | coef | se | z | p |
|---|---|---|---|---|
| HIV/H9, Hela | 1.360 | 0.1180 | 11.60 | 4.62e-31 |
| HIV/IMR90 | 1.080 | 0.1090 | 9.92 | 3.39e-23 |
| HIV/PBMC | 1.400 | 0.1000 | 14.00 | 2.85e-44 |
| HIV/SupT1 | 1.180 | 0.1120 | 10.50 | 9.76e-26 |
| ASLV/293T-TVA | 0.602 | 0.1300 | 4.64 | 3.55e-06 |
| MLV/Hela | 0.915 | 0.0885 | 10.30 | 4.41e-25 |

Now we count up to 200 ESTs per gene:

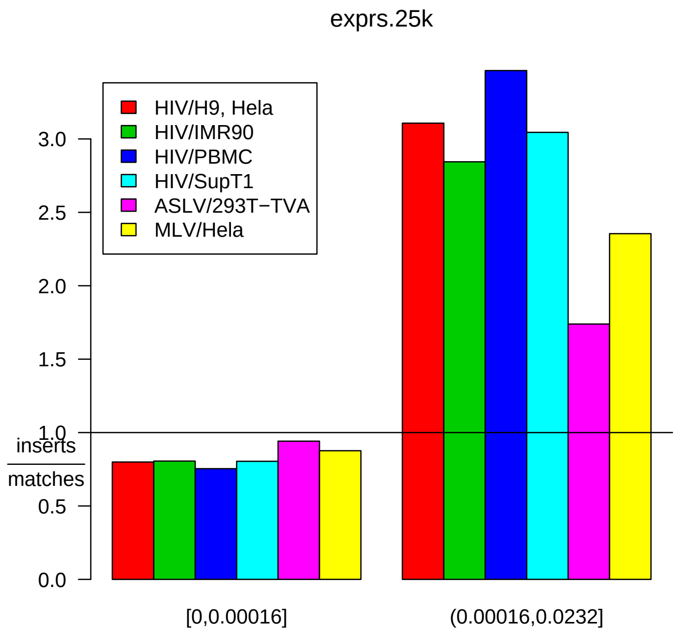


| | coef | se | z | p |
|---|---|---|---|---|
| HIV/H9, Hela | 1.360 | 0.1180 | 11.60 | 4.62e-31 |
| HIV/IMR90 | 1.080 | 0.1090 | 9.92 | 3.39e-23 |
| HIV/PBMC | 1.400 | 0.1000 | 14.00 | 2.85e-44 |
| HIV/SupT1 | 1.180 | 0.1120 | 10.50 | 9.76e-26 |
| ASLV/293T-TVA | 0.602 | 0.1300 | 4.64 | 3.55e-06 |
| MLV/Hela | 0.915 | 0.0885 | 10.30 | 4.41e-25 |

And here counting starts only after 200 ESTs per gene

big.exprs.25k

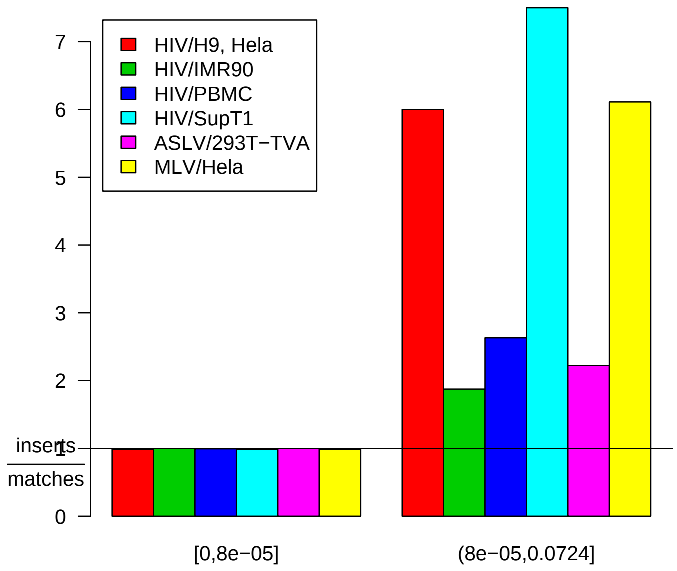


| | coef | se | z | p |
|---|---|---|---|---|
| HIV/H9, Hela | 1.700 | 0.508 | 3.340 | 8.30e-04 |
| HIV/IMR90 | 0.629 | 0.629 | 0.999 | 3.18e-01 |
| HIV/PBMC | 0.887 | 0.505 | 1.760 | 7.92e-02 |
| HIV/SupT1 | 2.010 | 0.540 | 3.730 | 1.91e-04 |
| ASLV/293T-TVA | 1.200 | 0.667 | 1.810 | 7.09e-02 |
| MLV/Hela | 1.810 | 0.383 | 4.730 | 2.25e-06 |

Here the effect of density of CpG islands is studied:

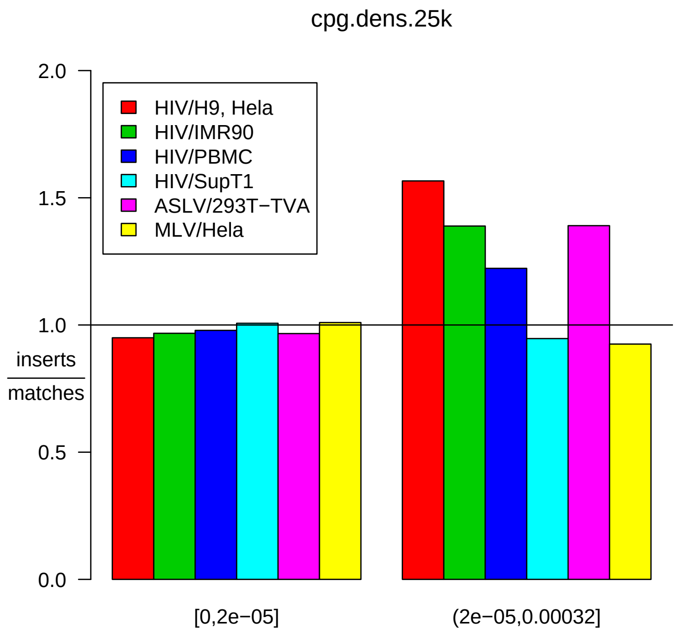


| | coef | se | z | p |
|---|---|---|---|---|
| HIV/H9, Hela | 0.2570 | 0.1130 | 2.290 | 0.0223 |
| HIV/IMR90 | 0.2220 | 0.1080 | 2.070 | 0.0387 |
| HIV/PBMC | 0.1760 | 0.1000 | 1.750 | 0.0795 |
| HIV/SupT1 | -0.1010 | 0.1140 | -0.887 | 0.3750 |
| ASLV/293T-TVA | 0.1270 | 0.1080 | 1.180 | 0.2390 |
| MLV/Hela | 0.0791 | 0.0795 | 0.995 | 0.3200 |

## 4.2 50 kiloBase Window

First, we see gene density:

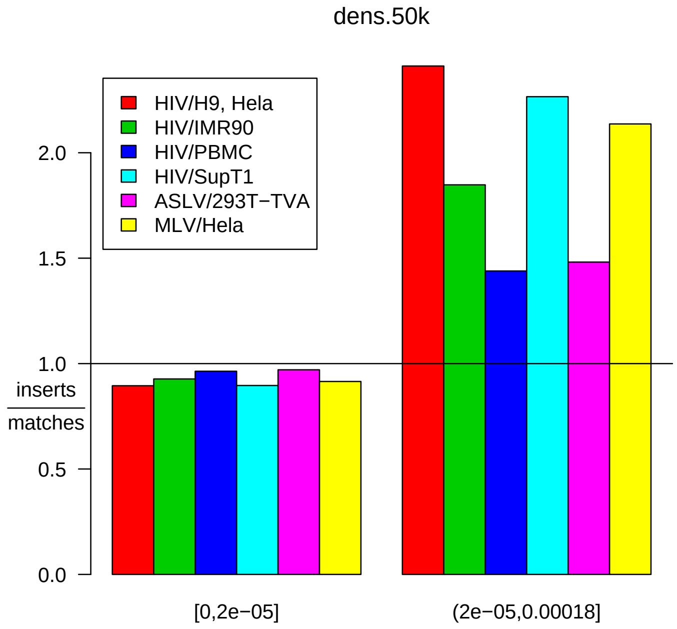


| | coef | se | z | p |
|---|---|---|---|---|
| HIV/H9, Hela | 1.260 | 0.1070 | 11.80 | 7.01e-32 |
| HIV/IMR90 | 0.786 | 0.0983 | 7.99 | 1.34e-15 |
| HIV/PBMC | 1.230 | 0.0939 | 13.10 | 5.10e-39 |
| HIV/SupT1 | 1.030 | 0.1020 | 10.10 | 6.29e-24 |
| ASLV/293T-TVA | 0.436 | 0.1010 | 4.30 | 1.69e-05 |
| MLV/Hela | 0.725 | 0.0742 | 9.76 | 1.60e-22 |

Here are the results for EST density. First, we count just one EST per gene.

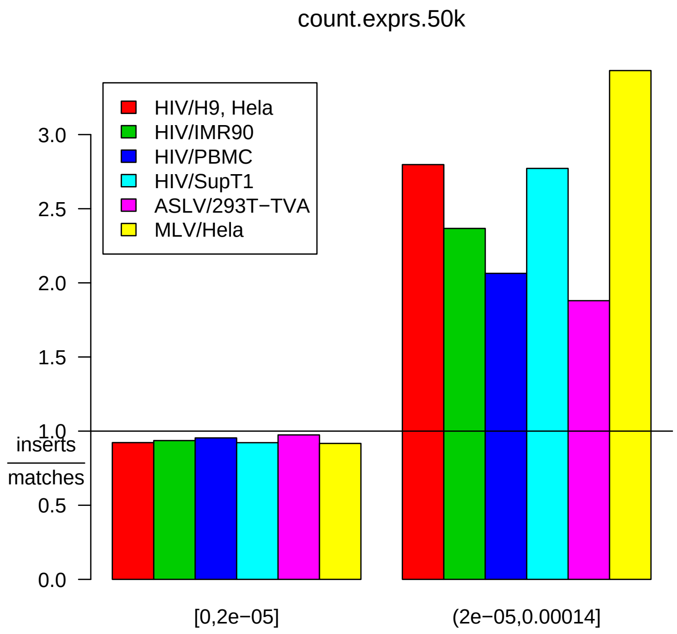


| | coef | se | z | p |
|---|---|---|---|---|
| HIV/H9, Hela | 1.410 | 0.1070 | 13.10 | 1.89e-39 |
| HIV/IMR90 | 1.020 | 0.1000 | 10.20 | 1.28e-24 |
| HIV/PBMC | 1.450 | 0.0947 | 15.30 | 1.31e-52 |
| HIV/SupT1 | 1.260 | 0.1020 | 12.30 | 1.02e-34 |
| ASLV/293T-TVA | 0.429 | 0.1100 | 3.89 | 1.01e-04 |
| MLV/Hela | 0.862 | 0.0766 | 11.30 | 2.13e-29 |

Now we count up to 200 ESTs per gene:

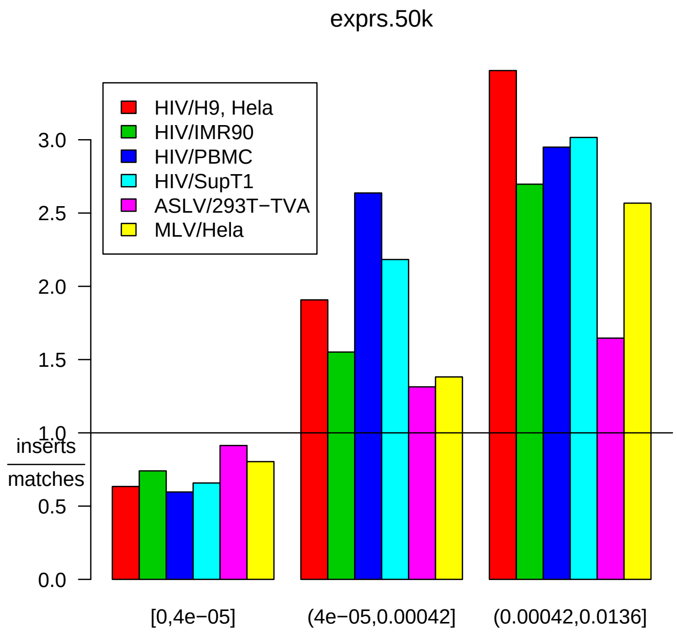


| | coef | se | z | p |
|---|---|---|---|---|
| HIV/H9, Hela | 1.410 | 0.1070 | 13.10 | 1.89e-39 |
| HIV/IMR90 | 1.020 | 0.1000 | 10.20 | 1.28e-24 |
| HIV/PBMC | 1.450 | 0.0947 | 15.30 | 1.31e-52 |
| HIV/SupT1 | 1.260 | 0.1020 | 12.30 | 1.02e-34 |
| ASLV/293T-TVA | 0.429 | 0.1100 | 3.89 | 1.01e-04 |
| MLV/Hela | 0.862 | 0.0766 | 11.30 | 2.13e-29 |

And here counting starts only after 200 ESTs per gene

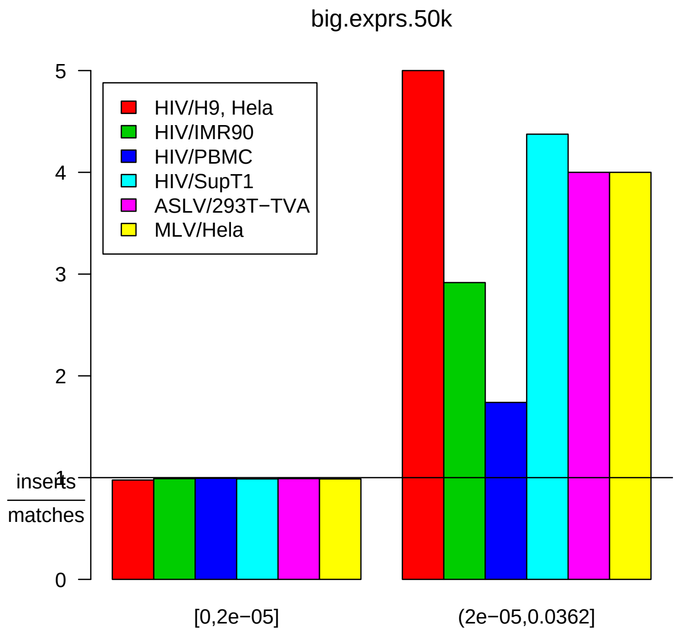


| | coef | se | z | p |
|---|---|---|---|---|
| HIV/H9, Hela | 1.630 | 0.357 | 4.56 | 5.13e-06 |
| HIV/IMR90 | 1.080 | 0.433 | 2.50 | 1.25e-02 |
| HIV/PBMC | 0.561 | 0.386 | 1.45 | 1.46e-01 |
| HIV/SupT1 | 1.530 | 0.466 | 3.27 | 1.06e-03 |
| ASLV/293T-TVA | 1.410 | 0.490 | 2.88 | 3.98e-03 |
| MLV/Hela | 1.380 | 0.319 | 4.32 | 1.53e-05 |

Here the effect of density of CpG islands is studied:

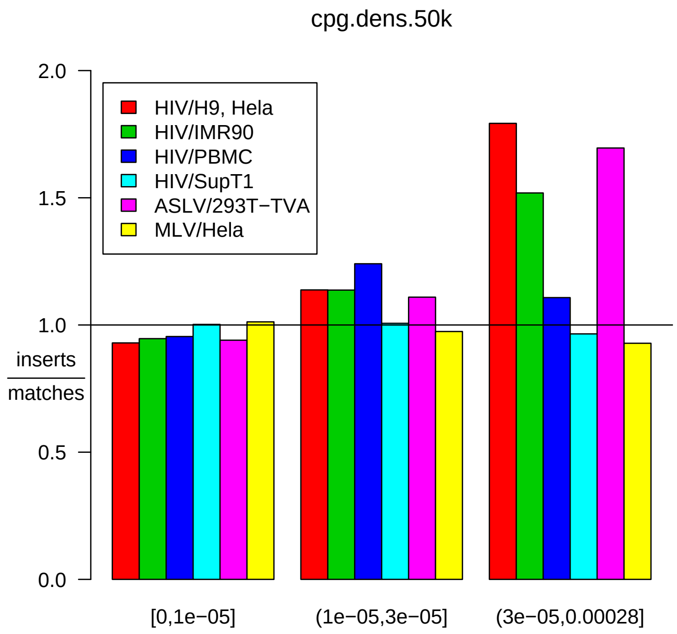


| | coef | se | z | p |
|---|---|---|---|---|
| HIV/H9, Hela | 0.2850 | 0.1040 | 2.730 | 0.00626 |
| HIV/IMR90 | 0.2210 | 0.0983 | 2.250 | 0.02460 |
| HIV/PBMC | 0.2780 | 0.0917 | 3.030 | 0.00241 |
| HIV/SupT1 | -0.0496 | 0.1020 | -0.488 | 0.62500 |
| ASLV/293T-TVA | 0.1410 | 0.0981 | 1.440 | 0.15000 |
| MLV/Hela | 0.0574 | 0.0737 | 0.779 | 0.43600 |

### 4.3 100 kiloBase Window

First, we see gene density:

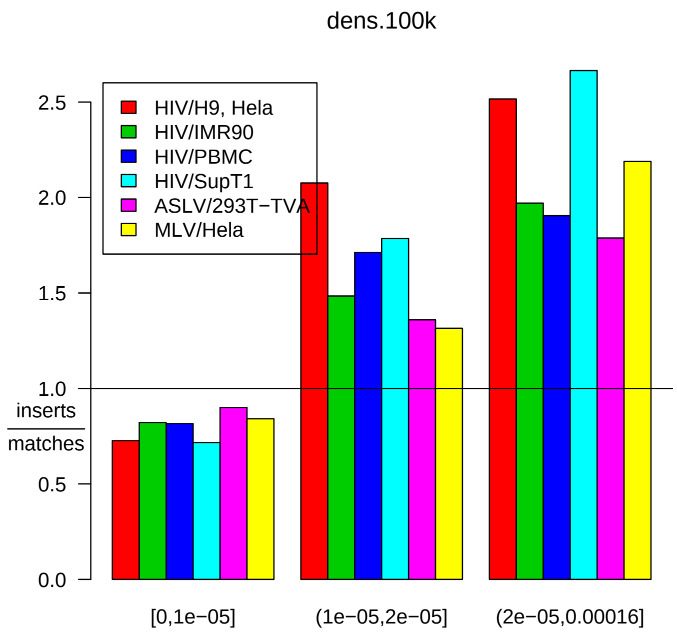


| | coef | se | z | p |
|---|---|---|---|---|
| HIV/H9, Hela | 1.400 | 0.1220 | 11.50 | 8.89e-31 |
| HIV/IMR90 | 0.909 | 0.1040 | 8.73 | 2.59e-18 |
| HIV/PBMC | 1.380 | 0.1080 | 12.70 | 5.76e-37 |
| HIV/SupT1 | 1.300 | 0.1170 | 11.10 | 1.14e-28 |
| ASLV/293T-TVA | 0.531 | 0.0977 | 5.44 | 5.42e-08 |
| MLV/Hela | 0.760 | 0.0765 | 9.94 | 2.83e-23 |

Here are the results for EST density. First, we count just one EST per gene.

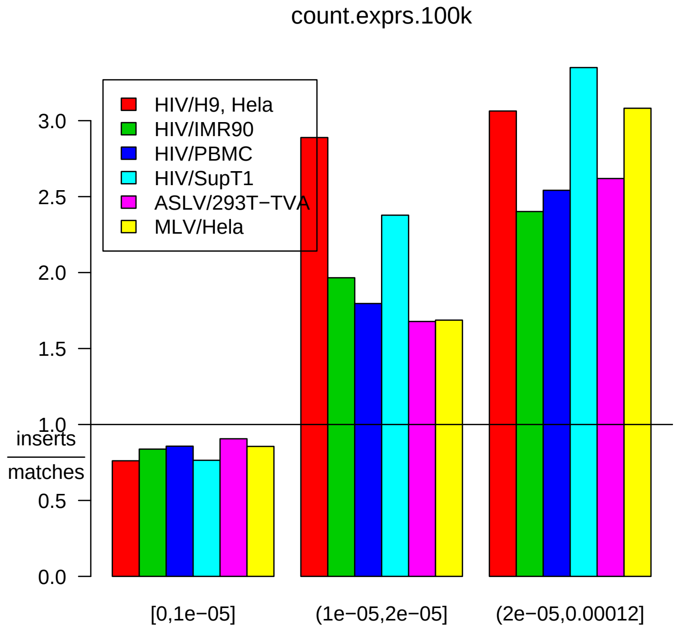


| | coef | se | z | p |
|---|---|---|---|---|
| HIV/H9, Hela | 1.580 | 0.1150 | 13.70 | 9.36e-43 |
| HIV/IMR90 | 1.120 | 0.1010 | 11.00 | 3.38e-28 |
| HIV/PBMC | 1.530 | 0.1020 | 15.00 | 5.07e-51 |
| HIV/SupT1 | 1.500 | 0.1100 | 13.60 | 3.45e-42 |
| ASLV/293T-TVA | 0.555 | 0.0977 | 5.68 | 1.37e-08 |
| MLV/Hela | 0.906 | 0.0744 | 12.20 | 4.66e-34 |

Now we count up to 200 ESTs per gene:

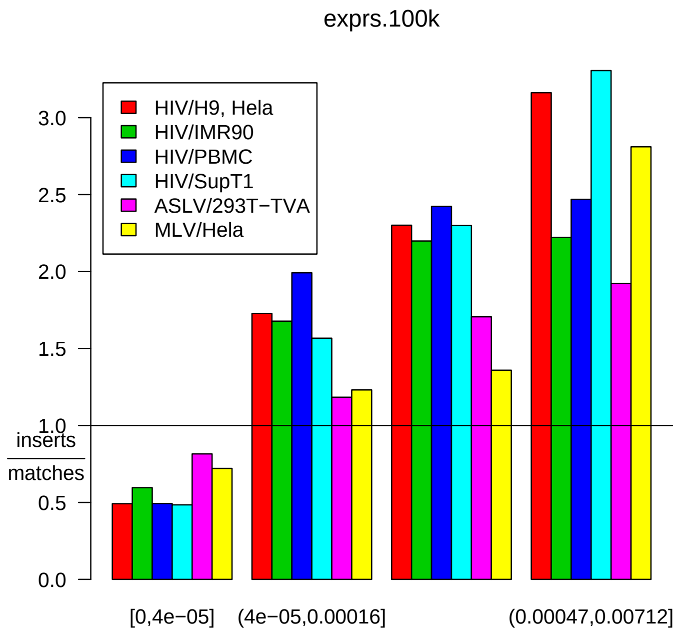


| | coef | se | z | p |
|---|---|---|---|---|
| HIV/H9, Hela | 1.580 | 0.1150 | 13.70 | 9.36e-43 |
| HIV/IMR90 | 1.120 | 0.1010 | 11.00 | 3.38e-28 |
| HIV/PBMC | 1.530 | 0.1020 | 15.00 | 5.07e-51 |
| HIV/SupT1 | 1.500 | 0.1100 | 13.60 | 3.45e-42 |
| ASLV/293T-TVA | 0.555 | 0.0977 | 5.68 | 1.37e-08 |
| MLV/Hela | 0.906 | 0.0744 | 12.20 | 4.66e-34 |

And here counting starts only after 200 ESTs per gene

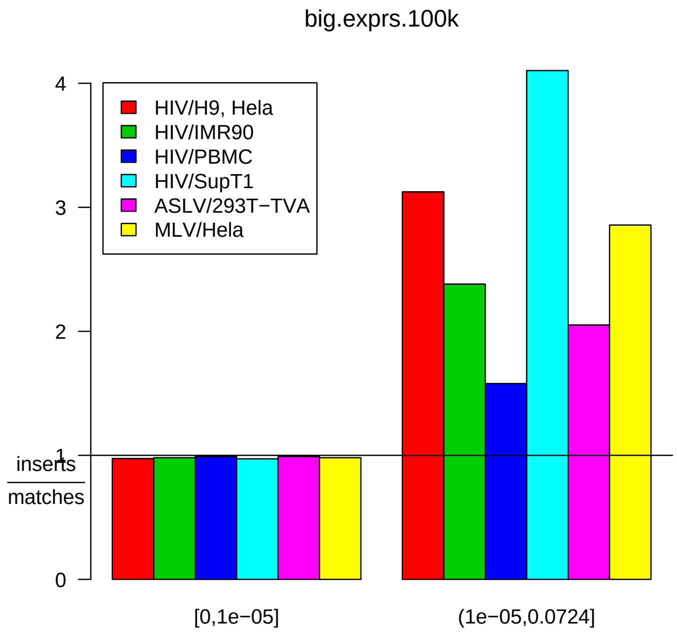


| | coef | se | z | p |
|---|---|---|---|---|
| HIV/H9, Hela | 1.240 | 0.295 | 4.20 | 2.71e-05 |
| HIV/IMR90 | 0.894 | 0.293 | 3.05 | 2.31e-03 |
| HIV/PBMC | 0.451 | 0.313 | 1.44 | 1.50e-01 |
| HIV/SupT1 | 1.450 | 0.304 | 4.78 | 1.77e-06 |
| ASLV/293T-TVA | 0.727 | 0.391 | 1.86 | 6.31e-02 |
| MLV/Hela | 1.060 | 0.234 | 4.51 | 6.43e-06 |

Here the effect of density of CpG islands is studied:

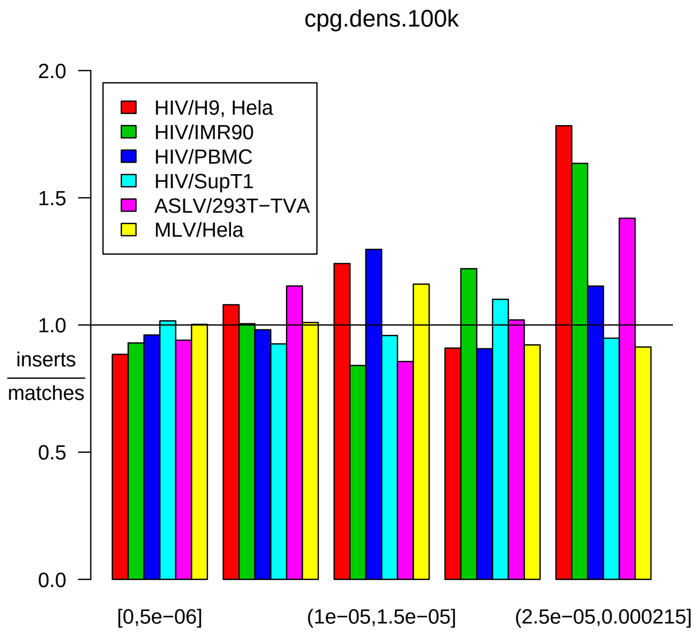


| | coef | se | z | p |
|---|---|---|---|---|
| HIV/H9, Hela | 0.3260 | 0.1060 | 3.0800 | 0.00205 |
| HIV/IMR90 | 0.2040 | 0.1000 | 2.0400 | 0.04160 |
| HIV/PBMC | 0.1100 | 0.0946 | 1.1700 | 0.24400 |
| HIV/SupT1 | -0.0430 | 0.1040 | -0.4140 | 0.67900 |
| ASLV/293T-TVA | 0.1700 | 0.1000 | 1.7000 | 0.08890 |
| MLV/Hela | -0.0067 | 0.0753 | -0.0889 | 0.92900 |

### 4.4 250 kiloBase Window

First, we see gene density:

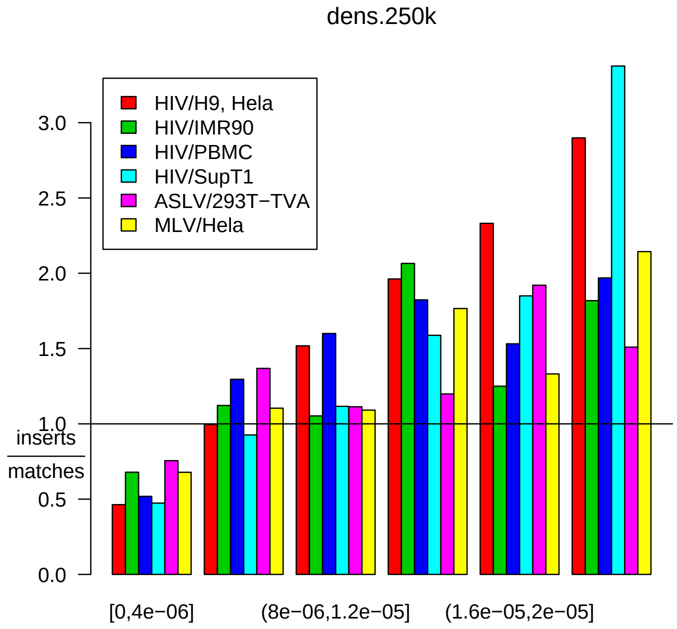


| | coef | se | z | p |
|---|---|---|---|---|
| HIV/H9, Hela | 1.310 | 0.1170 | 11.20 | 3.29e-29 |
| HIV/IMR90 | 0.710 | 0.1010 | 7.05 | 1.79e-12 |
| HIV/PBMC | 1.120 | 0.1000 | 11.10 | 8.05e-29 |
| HIV/SupT1 | 1.230 | 0.1140 | 10.80 | 3.18e-27 |
| ASLV/293T-TVA | 0.576 | 0.0973 | 5.92 | 3.24e-09 |
| MLV/Hela | 0.736 | 0.0757 | 9.72 | 2.43e-22 |

Here are the results for EST density. First, we count just one EST per gene.

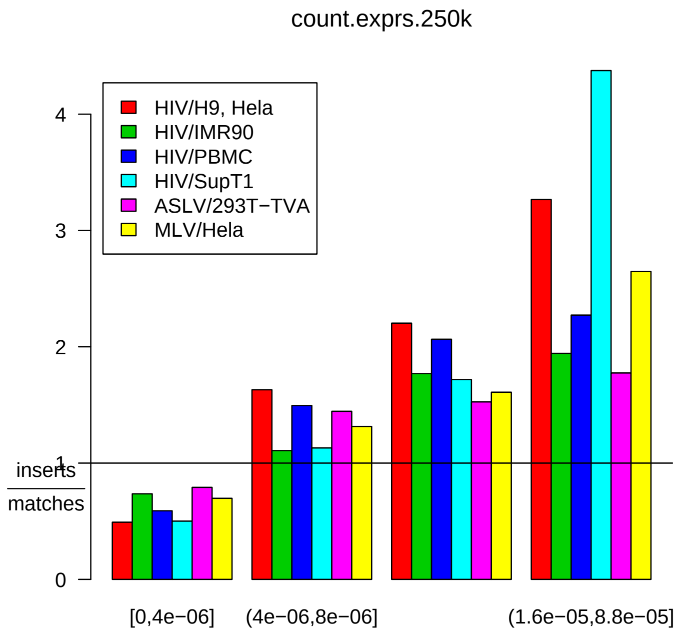


| | coef | se | z | p |
|---|---|---|---|---|
| HIV/H9, Hela | 1.510 | 0.1110 | 13.50 | 1.33e-41 |
| HIV/IMR90 | 0.739 | 0.0977 | 7.56 | 4.11e-14 |
| HIV/PBMC | 1.160 | 0.0942 | 12.30 | 5.63e-35 |
| HIV/SupT1 | 1.420 | 0.1070 | 13.20 | 7.62e-40 |
| ASLV/293T-TVA | 0.654 | 0.0982 | 6.66 | 2.72e-11 |
| MLV/Hela | 0.888 | 0.0742 | 12.00 | 6.10e-33 |

Now we count up to 200 ESTs per gene:

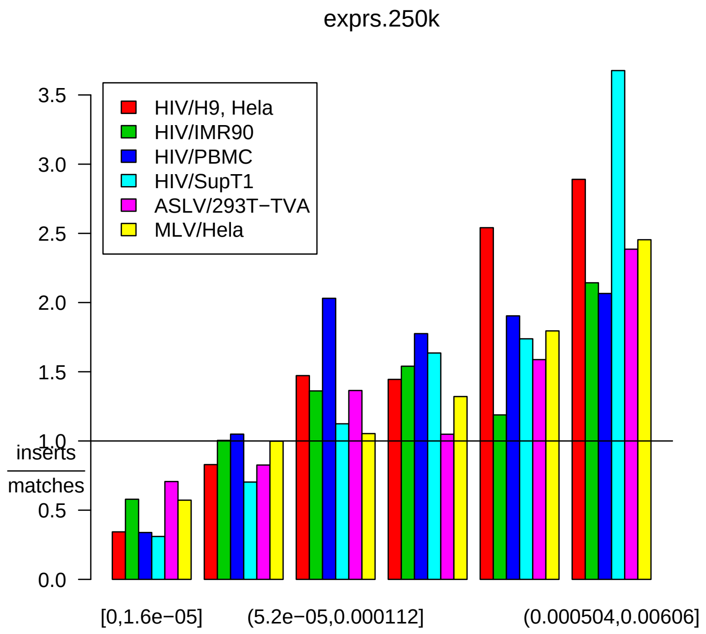


| | coef | se | z | p |
|---|---|---|---|---|
| HIV/H9, Hela | 1.680 | 0.1320 | 12.80 | 2.66e-37 |
| HIV/IMR90 | 0.918 | 0.1060 | 8.68 | 3.83e-18 |
| HIV/PBMC | 1.660 | 0.1170 | 14.20 | 9.72e-46 |
| HIV/SupT1 | 1.760 | 0.1340 | 13.10 | 4.57e-39 |
| ASLV/293T-TVA | 0.661 | 0.0983 | 6.73 | 1.73e-11 |
| MLV/Hela | 0.976 | 0.0790 | 12.30 | 5.10e-35 |

And here counting starts only after 200 ESTs per gene

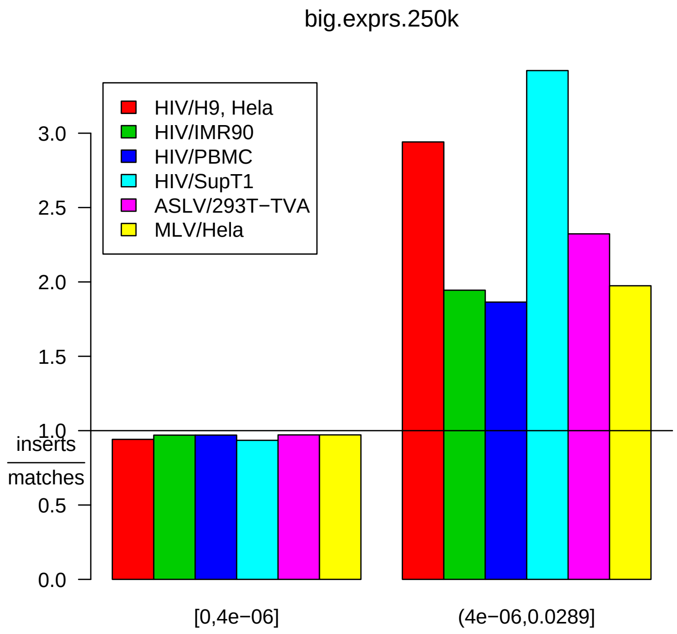


| | coef | se | z | p |
|---|---|---|---|---|
| HIV/H9, Hela | 1.150 | 0.197 | 5.82 | 6.00e-09 |
| HIV/IMR90 | 0.695 | 0.212 | 3.27 | 1.07e-03 |
| HIV/PBMC | 0.644 | 0.195 | 3.31 | 9.39e-04 |
| HIV/SupT1 | 1.310 | 0.195 | 6.74 | 1.61e-11 |
| ASLV/293T-TVA | 0.861 | 0.236 | 3.64 | 2.73e-04 |
| MLV/Hela | 0.704 | 0.166 | 4.25 | 2.14e-05 |

Here the effect of density of CpG islands is studied:

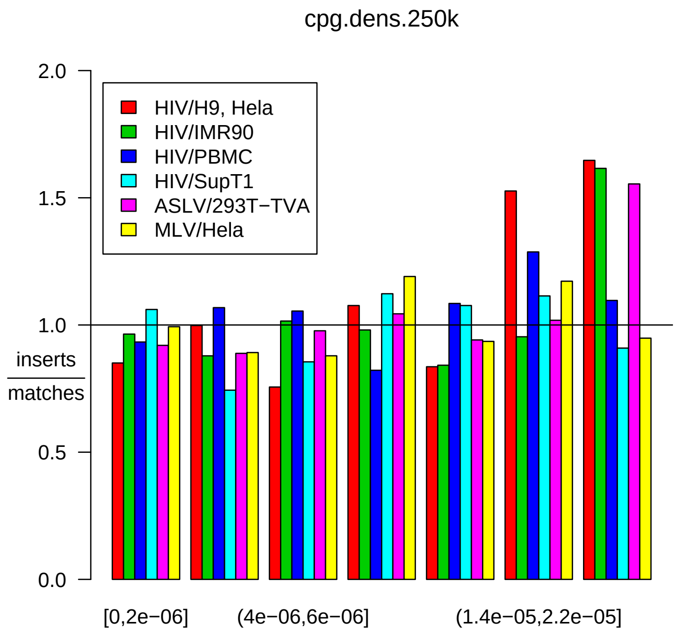


| | coef | se | z | p |
|---|---|---|---|---|
| HIV/H9, Hela | 0.2410 | 0.1040 | 2.320 | 0.0201 |
| HIV/IMR90 | 0.1170 | 0.0970 | 1.210 | 0.2270 |
| HIV/PBMC | 0.0733 | 0.0916 | 0.800 | 0.4240 |
| HIV/SupT1 | 0.0284 | 0.1000 | 0.283 | 0.7770 |
| ASLV/293T-TVA | 0.1870 | 0.0971 | 1.920 | 0.0543 |
| MLV/Hela | 0.0619 | 0.0733 | 0.845 | 0.3980 |

## 4.5 500 kiloBase Window

First, we see gene density:

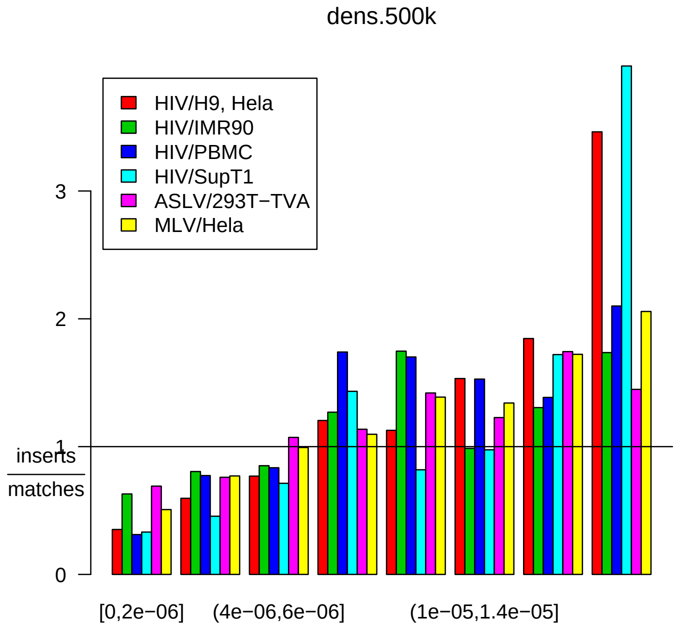


| | coef | se | z | p |
|---|---|---|---|---|
| HIV/H9, Hela | 1.310 | 0.1140 | 11.50 | 1.14e-30 |
| HIV/IMR90 | 0.659 | 0.0994 | 6.63 | 3.42e-11 |
| HIV/PBMC | 1.190 | 0.0993 | 11.90 | 7.31e-33 |
| HIV/SupT1 | 1.390 | 0.1150 | 12.10 | 1.01e-33 |
| ASLV/293T-TVA | 0.563 | 0.0971 | 5.80 | 6.54e-09 |
| MLV/Hela | 0.827 | 0.0752 | 11.00 | 3.79e-28 |

Here are the results for EST density. First, we count just one EST per gene.

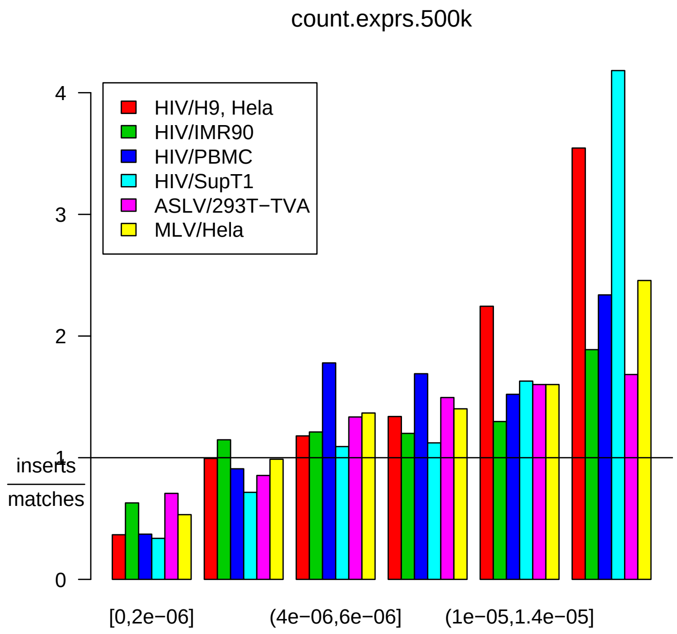


| | coef | se | z | p |
|---|---|---|---|---|
| HIV/H9, Hela | 1.360 | 0.1130 | 12.10 | 1.46e-33 |
| HIV/IMR90 | 0.617 | 0.0985 | 6.26 | 3.79e-10 |
| HIV/PBMC | 1.320 | 0.0996 | 13.30 | 3.81e-40 |
| HIV/SupT1 | 1.480 | 0.1140 | 13.00 | 1.43e-38 |
| ASLV/293T-TVA | 0.702 | 0.0970 | 7.24 | 4.35e-13 |
| MLV/Hela | 0.953 | 0.0752 | 12.70 | 7.90e-37 |

Now we count up to 200 ESTs per gene:

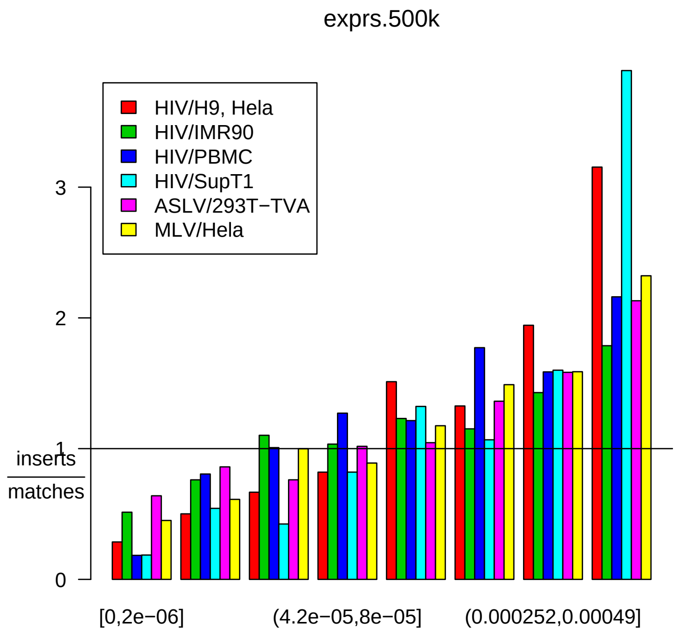


| | coef | se | z | p |
|---|---|---|---|---|
| HIV/H9, Hela | 1.490 | 0.1250 | 11.90 | 1.33e-32 |
| HIV/IMR90 | 0.675 | 0.1020 | 6.59 | 4.48e-11 |
| HIV/PBMC | 1.270 | 0.1070 | 11.90 | 1.64e-32 |
| HIV/SupT1 | 1.750 | 0.1390 | 12.60 | 1.57e-36 |
| ASLV/293T-TVA | 0.677 | 0.0989 | 6.84 | 7.72e-12 |
| MLV/Hela | 0.926 | 0.0789 | 11.70 | 8.32e-32 |

And here counting starts only after 200 ESTs per gene

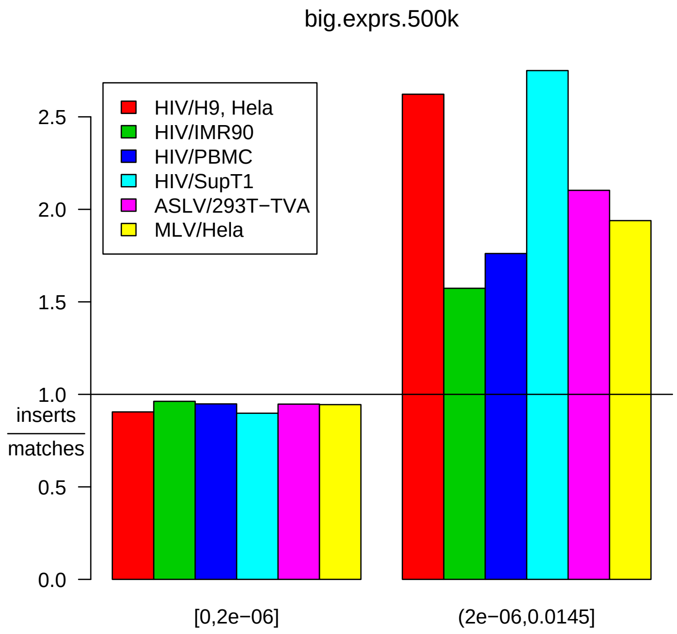


| | coef | se | z | p |
|---|---|---|---|---|
| HIV/H9, Hela | 1.080 | 0.157 | 6.89 | 5.45e-12 |
| HIV/IMR90 | 0.512 | 0.167 | 3.07 | 2.16e-03 |
| HIV/PBMC | 0.617 | 0.150 | 4.13 | 3.68e-05 |
| HIV/SupT1 | 1.120 | 0.151 | 7.42 | 1.14e-13 |
| ASLV/293T-TVA | 0.798 | 0.173 | 4.61 | 4.01e-06 |
| MLV/Hela | 0.716 | 0.123 | 5.84 | 5.21e-09 |

Here the effect of density of CpG islands is studied:

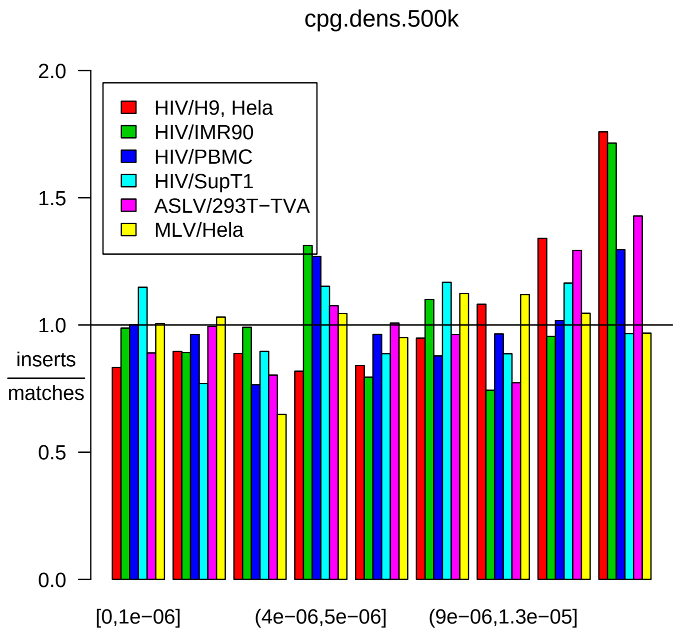


| | coef | se | z | p |
|---|---|---|---|---|
| HIV/H9, Hela | 0.2990 | 0.1040 | 2.880 | 0.00399 |
| HIV/IMR90 | 0.0104 | 0.0975 | 0.106 | 0.91500 |
| HIV/PBMC | 0.0228 | 0.0914 | 0.249 | 0.80300 |
| HIV/SupT1 | 0.0184 | 0.1010 | 0.183 | 0.85500 |
| ASLV/293T-TVA | 0.1440 | 0.0969 | 1.490 | 0.13600 |
| MLV/Hela | 0.0741 | 0.0735 | 1.010 | 0.31300 |

## 4.6 1 megaBase Window

First, we see gene density:

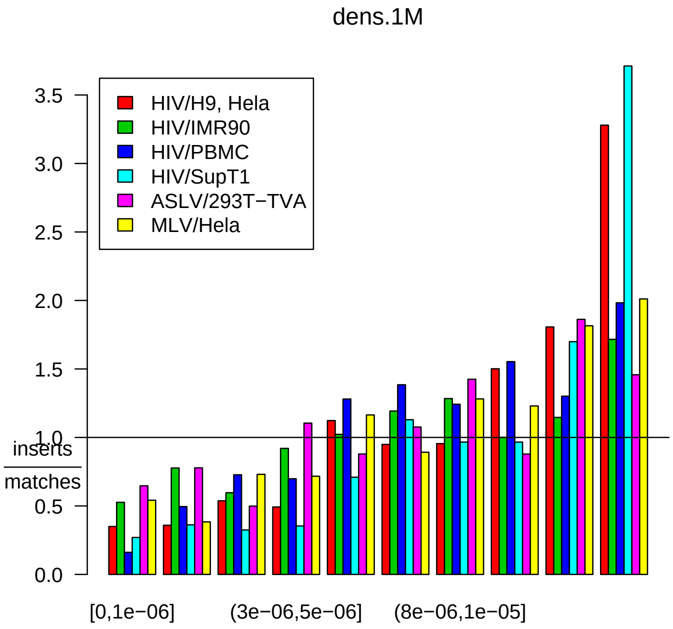


| | coef | se | z | p |
|---|---|---|---|---|
| HIV/H9, Hela | 1.160 | 0.1150 | 10.00 | 9.29e-24 |
| HIV/IMR90 | 0.536 | 0.0998 | 5.37 | 7.96e-08 |
| HIV/PBMC | 0.942 | 0.0980 | 9.62 | 6.57e-22 |
| HIV/SupT1 | 1.510 | 0.1250 | 12.10 | 6.38e-34 |
| ASLV/293T-TVA | 0.484 | 0.0971 | 4.99 | 6.09e-07 |
| MLV/Hela | 0.749 | 0.0761 | 9.84 | 7.30e-23 |

Here are the results for EST density. First, we count just one EST per gene.

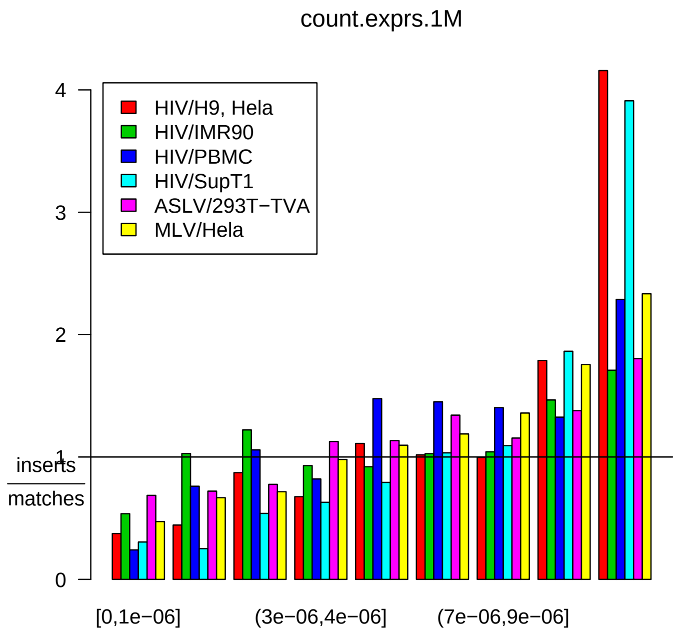


| | coef | se | z | p |
|---|---|---|---|---|
| HIV/H9, Hela | 1.230 | 0.1140 | 10.80 | 3.56e-27 |
| HIV/IMR90 | 0.449 | 0.0986 | 4.55 | 5.34e-06 |
| HIV/PBMC | 1.040 | 0.0974 | 10.60 | 2.01e-26 |
| HIV/SupT1 | 1.510 | 0.1210 | 12.40 | 1.58e-35 |
| ASLV/293T-TVA | 0.559 | 0.0975 | 5.74 | 9.70e-09 |
| MLV/Hela | 0.893 | 0.0764 | 11.70 | 1.50e-31 |

Now we count up to 200 ESTs per gene:

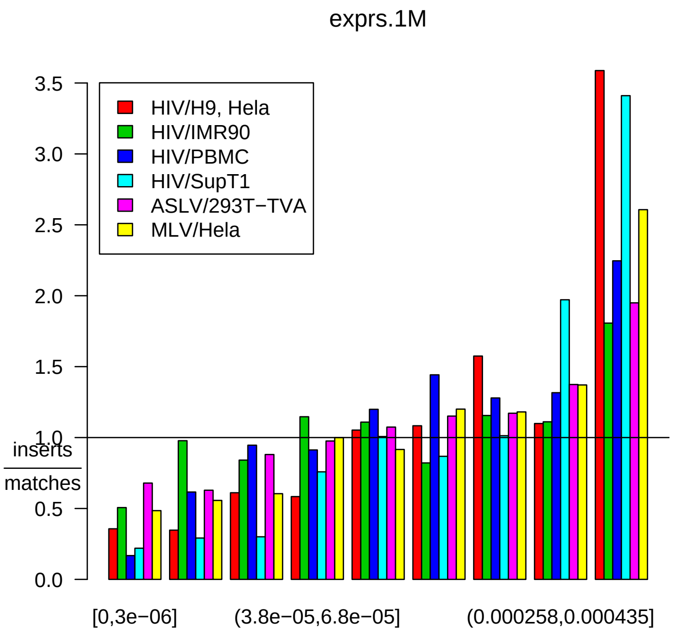


| | coef | se | z | p |
|---|---|---|---|---|
| HIV/H9, Hela | 1.330 | 0.1220 | 10.90 | 8.99e-28 |
| HIV/IMR90 | 0.427 | 0.1000 | 4.27 | 1.95e-05 |
| HIV/PBMC | 0.988 | 0.1000 | 9.84 | 7.58e-23 |
| HIV/SupT1 | 1.530 | 0.1300 | 11.80 | 4.19e-32 |
| ASLV/293T-TVA | 0.542 | 0.0982 | 5.52 | 3.44e-08 |
| MLV/Hela | 0.839 | 0.0782 | 10.70 | 7.20e-27 |

And here counting starts only after 200 ESTs per gene

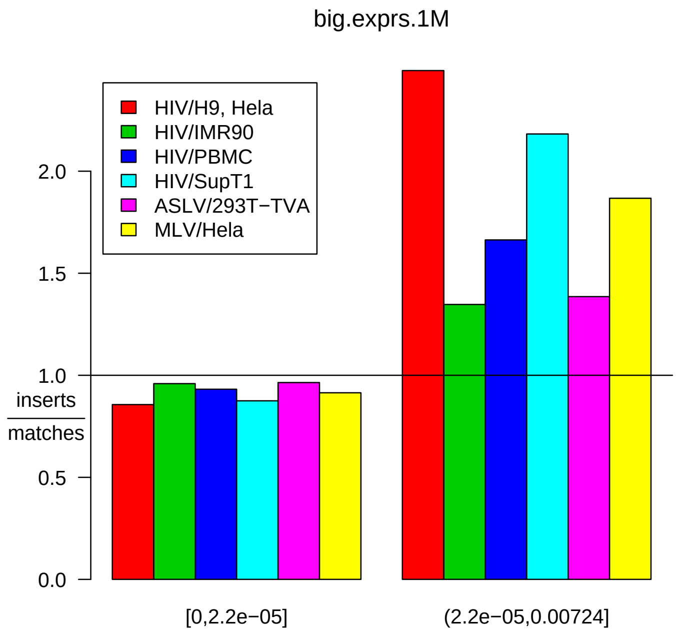


| | coef | se | z | p |
|---|---|---|---|---|
| HIV/H9, Hela | 1.060 | 0.1280 | 8.33 | 7.78e-17 |
| HIV/IMR90 | 0.305 | 0.1370 | 2.22 | 2.65e-02 |
| HIV/PBMC | 0.627 | 0.1210 | 5.20 | 1.99e-07 |
| HIV/SupT1 | 0.932 | 0.1230 | 7.58 | 3.48e-14 |
| ASLV/293T-TVA | 0.424 | 0.1460 | 2.91 | 3.64e-03 |
| MLV/Hela | 0.695 | 0.0972 | 7.16 | 8.35e-13 |

Here the effect of density of CpG islands is studied:

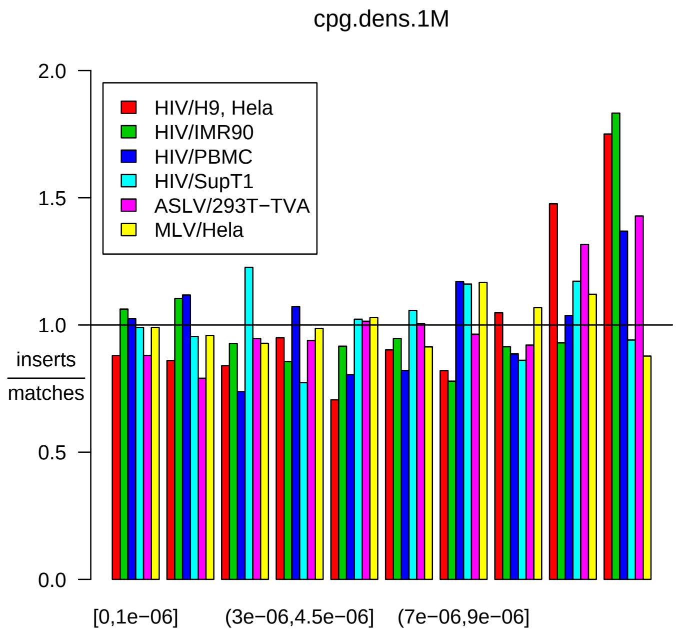


| | coef | se | z | p |
|---|---|---|---|---|
| HIV/H9, Hela | 0.3040 | 0.1040 | 2.910 | 0.00358 |
| HIV/IMR90 | 0.0502 | 0.0975 | 0.515 | 0.60700 |
| HIV/PBMC | 0.0695 | 0.0912 | 0.763 | 0.44600 |
| HIV/SupT1 | 0.0568 | 0.1000 | 0.566 | 0.57100 |
| ASLV/293T-TVA | 0.1900 | 0.0972 | 1.950 | 0.05080 |
| MLV/Hela | 0.0403 | 0.0731 | 0.551 | 0.58100 |

## 4.7 2 megaBase Window

First, we see gene density:

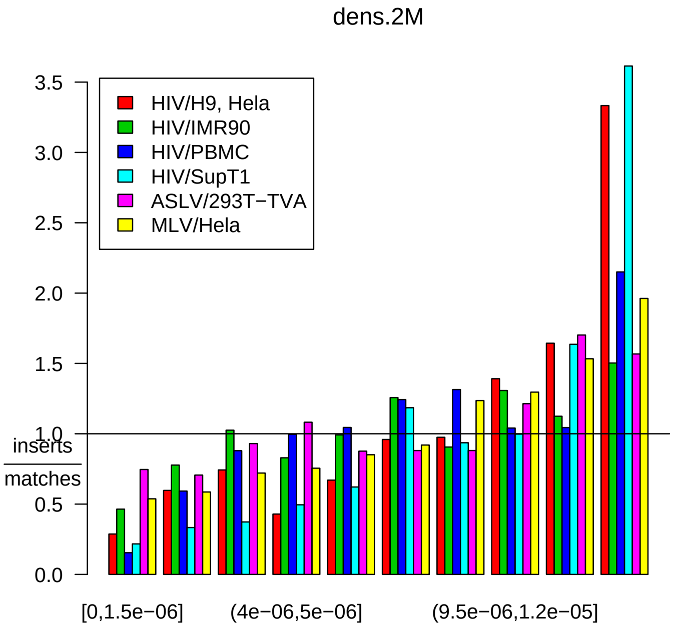


| | coef | se | z | p |
|---|---|---|---|---|
| HIV/H9, Hela | 1.110 | 0.1120 | 9.84 | 7.77e-23 |
| HIV/IMR90 | 0.409 | 0.0993 | 4.12 | 3.85e-05 |
| HIV/PBMC | 0.649 | 0.0936 | 6.93 | 4.30e-12 |
| HIV/SupT1 | 1.420 | 0.1200 | 11.80 | 2.86e-32 |
| ASLV/293T-TVA | 0.350 | 0.0969 | 3.61 | 3.04e-04 |
| MLV/Hela | 0.719 | 0.0756 | 9.51 | 1.85e-21 |

Here are the results for EST density. First, we count just one EST per gene.

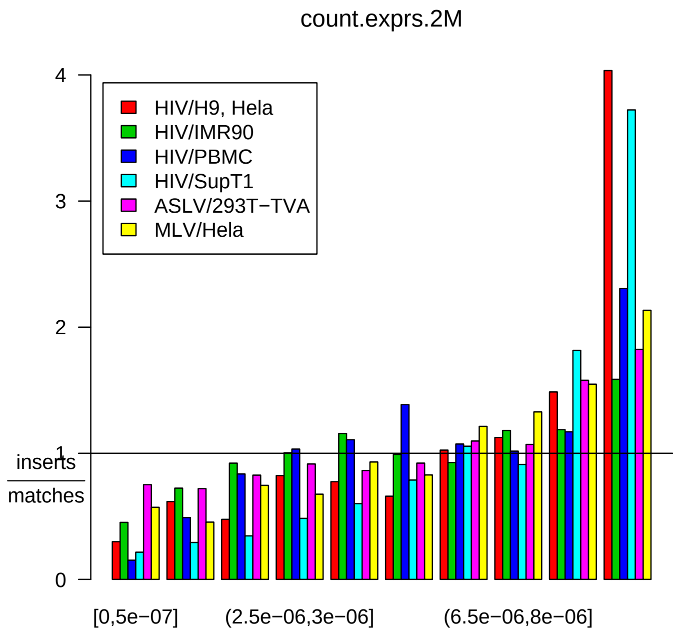


| | coef | se | z | p |
|---|---|---|---|---|
| HIV/H9, Hela | 1.040 | 0.1130 | 9.17 | 4.95e-20 |
| HIV/IMR90 | 0.353 | 0.0988 | 3.57 | 3.57e-04 |
| HIV/PBMC | 0.706 | 0.0948 | 7.45 | 9.65e-14 |
| HIV/SupT1 | 1.500 | 0.1260 | 11.90 | 1.18e-32 |
| ASLV/293T-TVA | 0.453 | 0.0973 | 4.65 | 3.29e-06 |
| MLV/Hela | 0.721 | 0.0759 | 9.49 | 2.30e-21 |

Now we count up to 200 ESTs per gene:

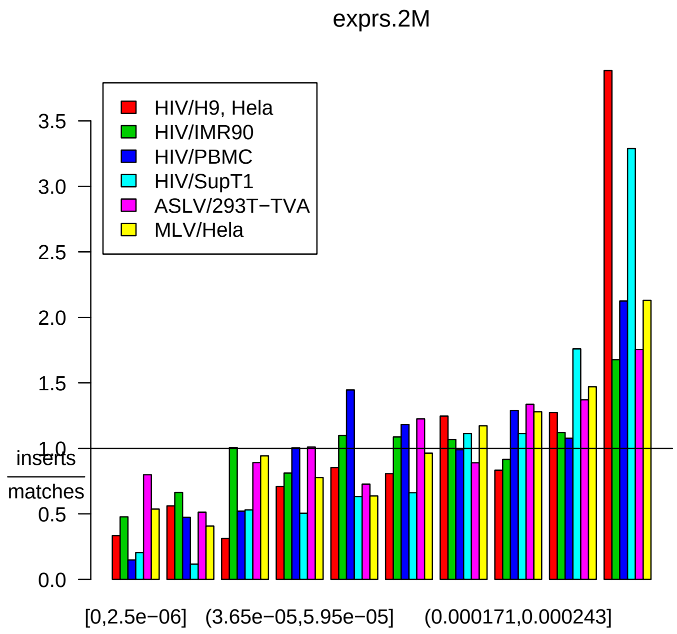


| | coef | se | z | p |
|---|---|---|---|---|
| HIV/H9, Hela | 1.030 | 0.1140 | 9.01 | 2.01e-19 |
| HIV/IMR90 | 0.373 | 0.0994 | 3.76 | 1.73e-04 |
| HIV/PBMC | 0.624 | 0.0944 | 6.62 | 3.71e-11 |
| HIV/SupT1 | 1.370 | 0.1260 | 11.00 | 6.29e-28 |
| ASLV/293T-TVA | 0.491 | 0.0983 | 4.99 | 5.89e-07 |
| MLV/Hela | 0.759 | 0.0774 | 9.80 | 1.16e-22 |

And here counting starts only after 200 ESTs per gene

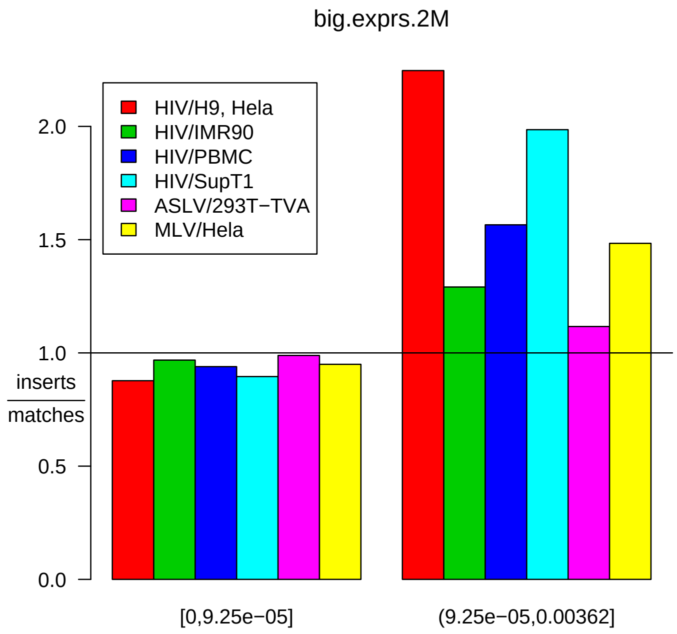


| | coef | se | z | p |
|---|---|---|---|---|
| HIV/H9, Hela | 0.836 | 0.1130 | 7.40 | 1.31e-13 |
| HIV/IMR90 | 0.214 | 0.1150 | 1.87 | 6.18e-02 |
| HIV/PBMC | 0.473 | 0.1030 | 4.60 | 4.25e-06 |
| HIV/SupT1 | 0.775 | 0.1070 | 7.22 | 5.03e-13 |
| ASLV/293T-TVA | 0.269 | 0.1190 | 2.26 | 2.38e-02 |
| MLV/Hela | 0.533 | 0.0824 | 6.47 | 9.93e-11 |

Here the effect of density of CpG islands is studied:

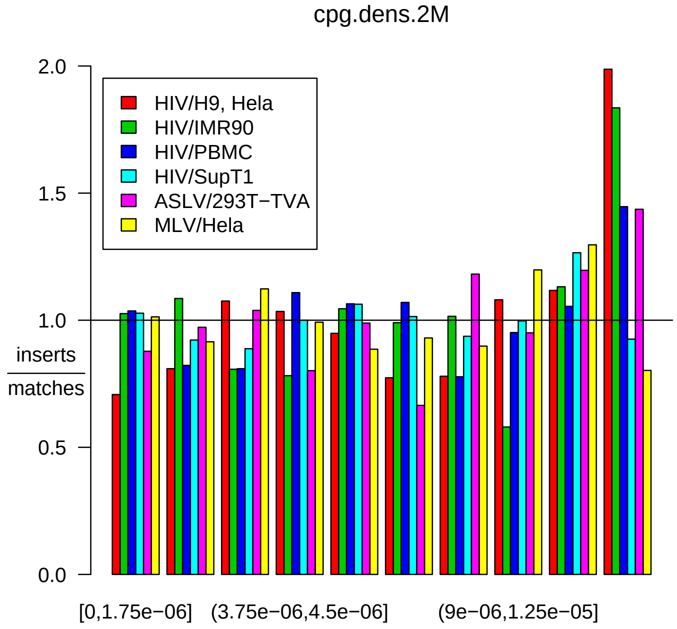


| | coef | se | z | p |
|---|---|---|---|---|
| HIV/H9, Hela | 0.2060 | 0.1040 | 1.980 | 0.0474 |
| HIV/IMR90 | 0.0999 | 0.0977 | 1.020 | 0.3070 |
| HIV/PBMC | 0.0690 | 0.0913 | 0.755 | 0.4500 |
| HIV/SupT1 | 0.0393 | 0.1000 | 0.392 | 0.6950 |
| ASLV/293T-TVA | 0.1430 | 0.0972 | 1.470 | 0.1420 |
| MLV/Hela | 0.0283 | 0.0733 | 0.386 | 0.6990 |

## 4.8 4 megaBase Window

First, we see gene density:

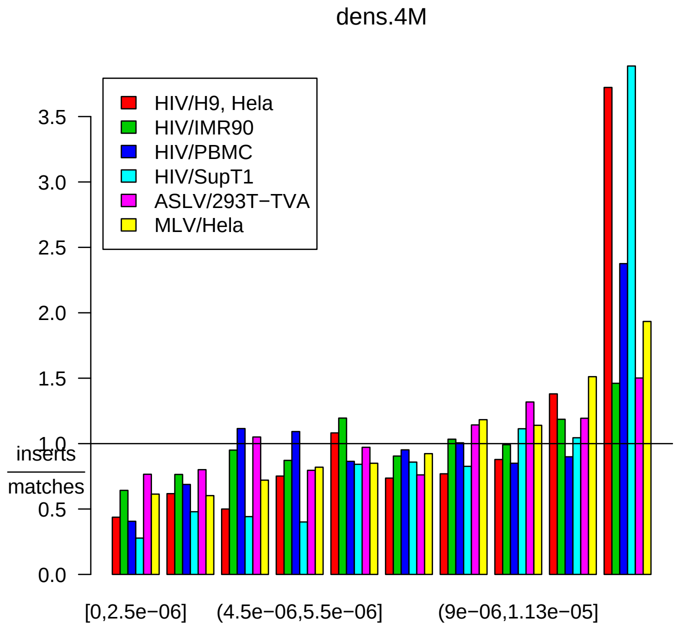


| | coef | se | z | p |
|---|---|---|---|---|
| HIV/H9, Hela | 0.775 | 0.1100 | 7.05 | 1.82e-12 |
| HIV/IMR90 | 0.249 | 0.0984 | 2.53 | 1.14e-02 |
| HIV/PBMC | 0.369 | 0.0922 | 4.00 | 6.36e-05 |
| HIV/SupT1 | 1.170 | 0.1160 | 10.10 | 5.50e-24 |
| ASLV/293T-TVA | 0.287 | 0.0969 | 2.96 | 3.08e-03 |
| MLV/Hela | 0.620 | 0.0755 | 8.22 | 2.06e-16 |

Here are the results for EST density. First, we count just one EST per gene.

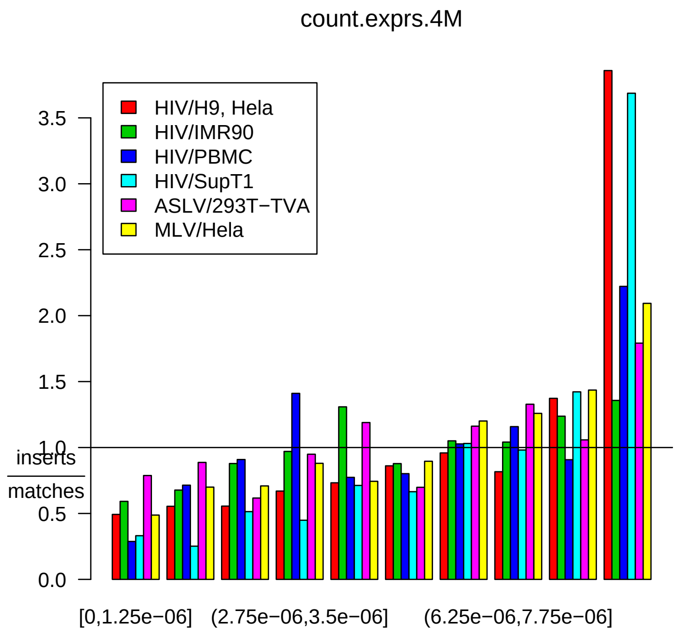


| | coef | se | z | p |
|---|---|---|---|---|
| HIV/H9, Hela | 0.928 | 0.1110 | 8.35 | 6.81e-17 |
| HIV/IMR90 | 0.252 | 0.0982 | 2.57 | 1.03e-02 |
| HIV/PBMC | 0.397 | 0.0925 | 4.29 | 1.83e-05 |
| HIV/SupT1 | 1.280 | 0.1190 | 10.80 | 5.19e-27 |
| ASLV/293T-TVA | 0.290 | 0.0972 | 2.99 | 2.83e-03 |
| MLV/Hela | 0.669 | 0.0754 | 8.88 | 6.90e-19 |

Now we count up to 200 ESTs per gene:

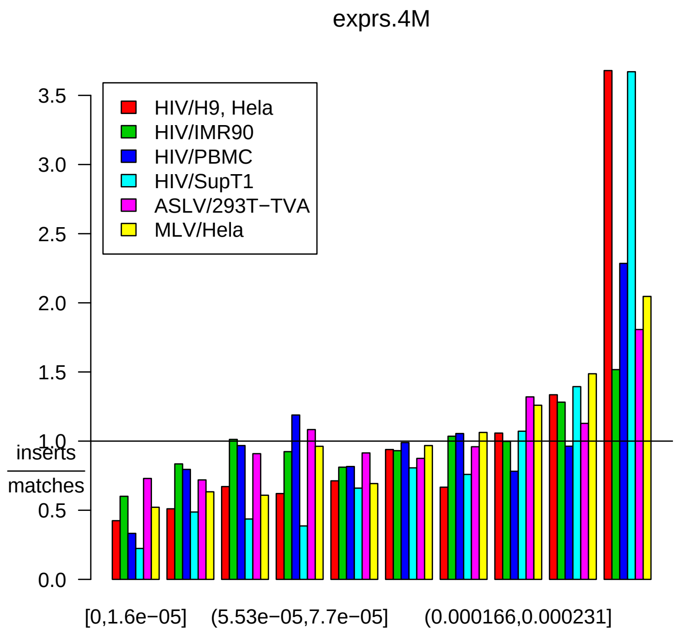


| | coef | se | z | p |
|---|---|---|---|---|
| HIV/H9, Hela | 0.918 | 0.1120 | 8.19 | 2.53e-16 |
| HIV/IMR90 | 0.334 | 0.0999 | 3.34 | 8.24e-04 |
| HIV/PBMC | 0.377 | 0.0925 | 4.07 | 4.71e-05 |
| HIV/SupT1 | 1.260 | 0.1210 | 10.40 | 2.08e-25 |
| ASLV/293T-TVA | 0.306 | 0.0973 | 3.14 | 1.67e-03 |
| MLV/Hela | 0.681 | 0.0766 | 8.89 | 5.93e-19 |

And here counting starts only after 200 ESTs per gene

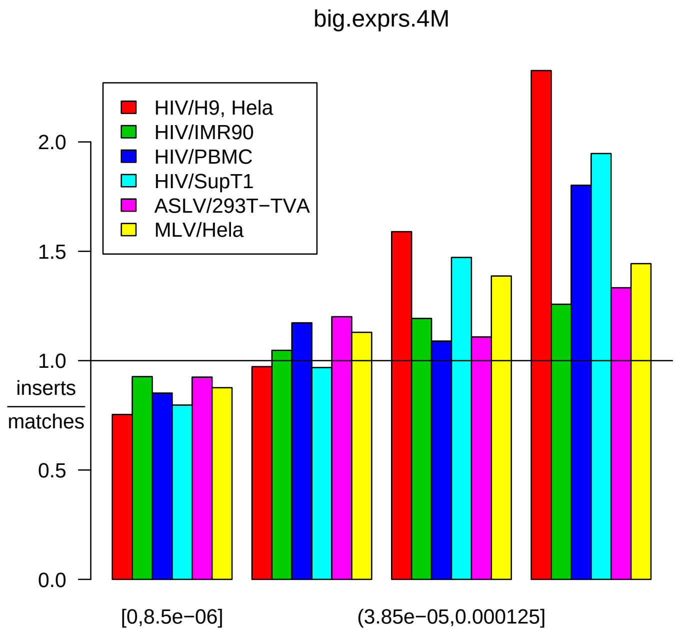


| | coef | se | z | p |
|---|---|---|---|---|
| HIV/H9, Hela | 0.726 | 0.1050 | 6.95 | 3.65e-12 |
| HIV/IMR90 | 0.262 | 0.0999 | 2.62 | 8.70e-03 |
| HIV/PBMC | 0.402 | 0.0924 | 4.35 | 1.37e-05 |
| HIV/SupT1 | 0.602 | 0.1010 | 5.98 | 2.20e-09 |
| ASLV/293T-TVA | 0.293 | 0.1020 | 2.89 | 3.89e-03 |
| MLV/Hela | 0.471 | 0.0744 | 6.33 | 2.47e-10 |

Here the effect of density of CpG islands is studied:

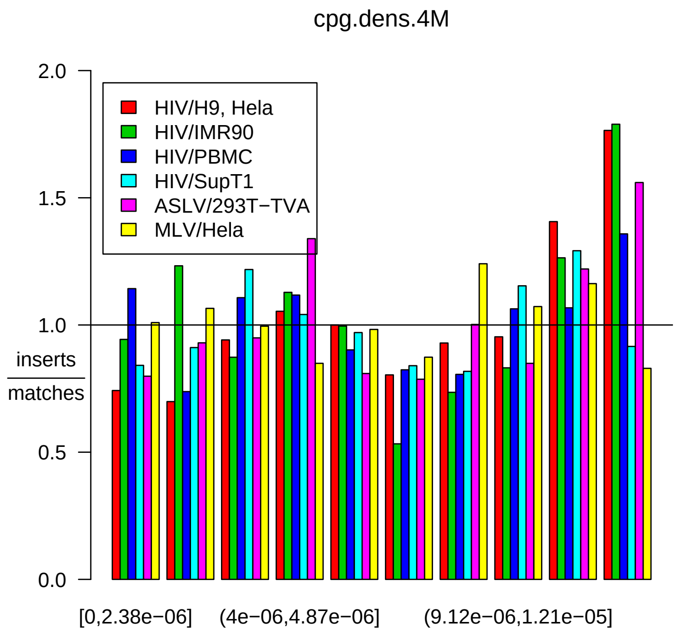


| | coef | se | z | p |
|---|---|---|---|---|
| HIV/H9, Hela | 0.24100 | 0.1040 | 2.3100 | 0.0206 |
| HIV/IMR90 | -0.07320 | 0.0974 | -0.7520 | 0.4520 |
| HIV/PBMC | 0.00377 | 0.0911 | 0.0414 | 0.9670 |
| HIV/SupT1 | -0.00275 | 0.1000 | -0.0274 | 0.9780 |
| ASLV/293T-TVA | 0.10200 | 0.0968 | 1.0600 | 0.2910 |
| MLV/Hela | 0.03270 | 0.0732 | 0.4460 | 0.6560 |

## 4.9 4 megaBase Window

First, we see gene density:

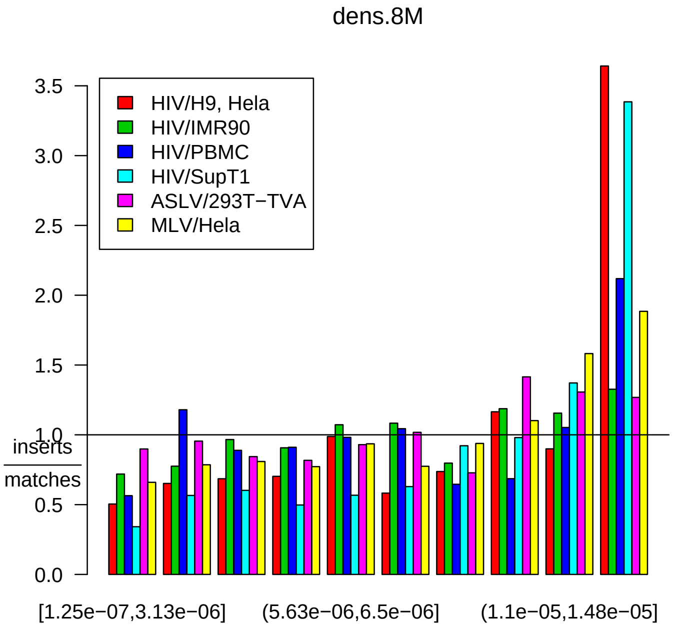


| | coef | se | z | p |
|---|---|---|---|---|
| HIV/H9, Hela | 0.649 | 0.1080 | 5.98 | 2.22e-09 |
| HIV/IMR90 | 0.232 | 0.0987 | 2.35 | 1.86e-02 |
| HIV/PBMC | 0.178 | 0.0915 | 1.94 | 5.23e-02 |
| HIV/SupT1 | 1.050 | 0.1150 | 9.11 | 7.97e-20 |
| ASLV/293T-TVA | 0.244 | 0.0971 | 2.51 | 1.20e-02 |
| MLV/Hela | 0.439 | 0.0746 | 5.89 | 3.87e-09 |

Here are the results for EST density. First, we count just one EST per gene.

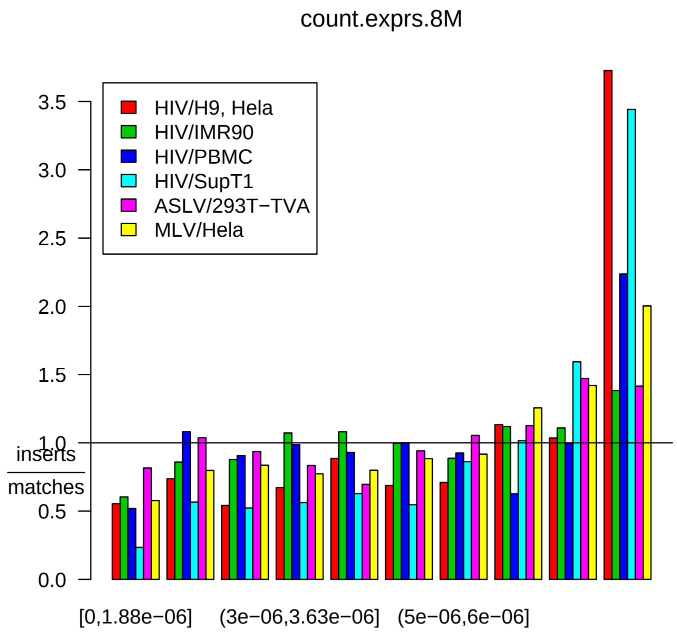


| | coef | se | z | p |
|---|---|---|---|---|
| HIV/H9, Hela | 0.724 | 0.1090 | 6.62 | 3.63e-11 |
| HIV/IMR90 | 0.217 | 0.0983 | 2.21 | 2.74e-02 |
| HIV/PBMC | 0.246 | 0.0919 | 2.68 | 7.47e-03 |
| HIV/SupT1 | 1.110 | 0.1160 | 9.55 | 1.32e-21 |
| ASLV/293T-TVA | 0.318 | 0.0970 | 3.28 | 1.04e-03 |
| MLV/Hela | 0.531 | 0.0751 | 7.06 | 1.61e-12 |

Now we count up to 200 ESTs per gene:

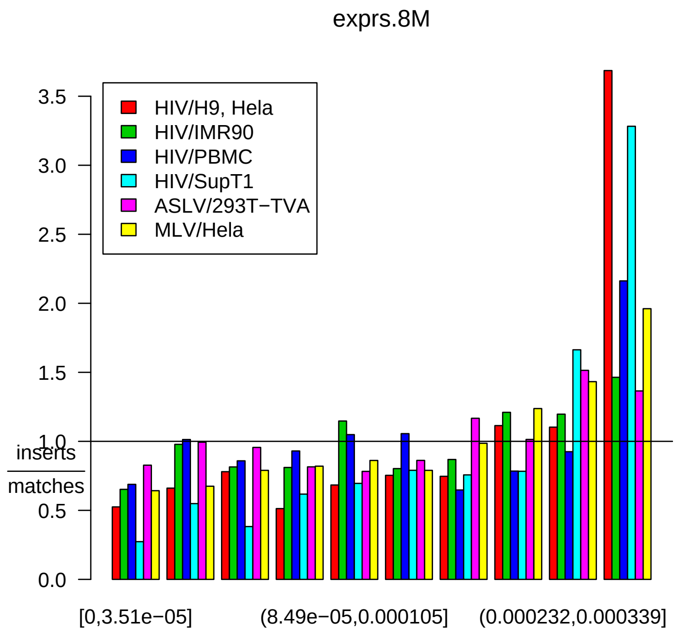


| | coef | se | z | p |
|---|---|---|---|---|
| HIV/H9, Hela | 0.794 | 0.1110 | 7.14 | 9.18e-13 |
| HIV/IMR90 | 0.233 | 0.0984 | 2.37 | 1.76e-02 |
| HIV/PBMC | 0.184 | 0.0916 | 2.01 | 4.40e-02 |
| HIV/SupT1 | 1.070 | 0.1160 | 9.28 | 1.63e-20 |
| ASLV/293T-TVA | 0.281 | 0.0973 | 2.89 | 3.85e-03 |
| MLV/Hela | 0.512 | 0.0751 | 6.82 | 9.00e-12 |

And here counting starts only after 200 ESTs per gene

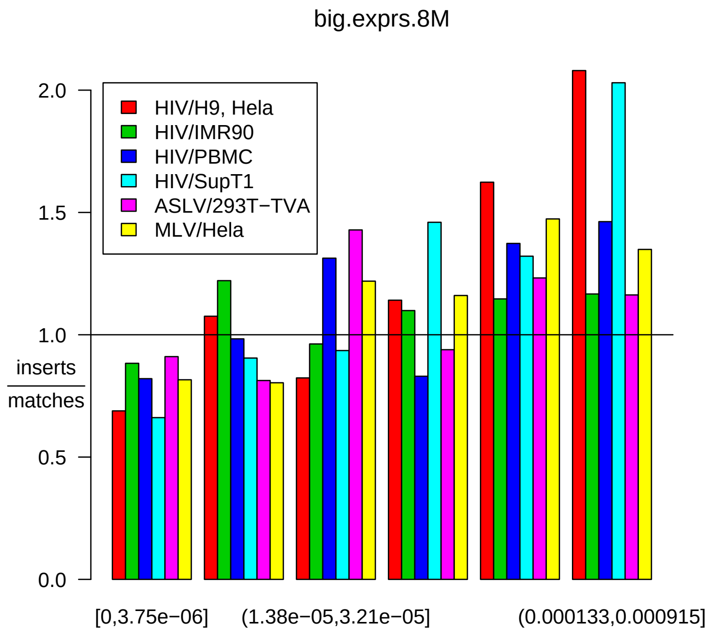


| | coef | se | z | p |
|---|---|---|---|---|
| HIV/H9, Hela | 0.664 | 0.1090 | 6.12 | 9.32e-10 |
| HIV/IMR90 | 0.235 | 0.0979 | 2.40 | 1.62e-02 |
| HIV/PBMC | 0.365 | 0.0927 | 3.94 | 8.06e-05 |
| HIV/SupT1 | 0.703 | 0.1060 | 6.62 | 3.71e-11 |
| ASLV/293T-TVA | 0.193 | 0.0973 | 1.98 | 4.75e-02 |
| MLV/Hela | 0.383 | 0.0743 | 5.16 | 2.44e-07 |

## 4.10 16 megaBase Window

First, we see gene density:

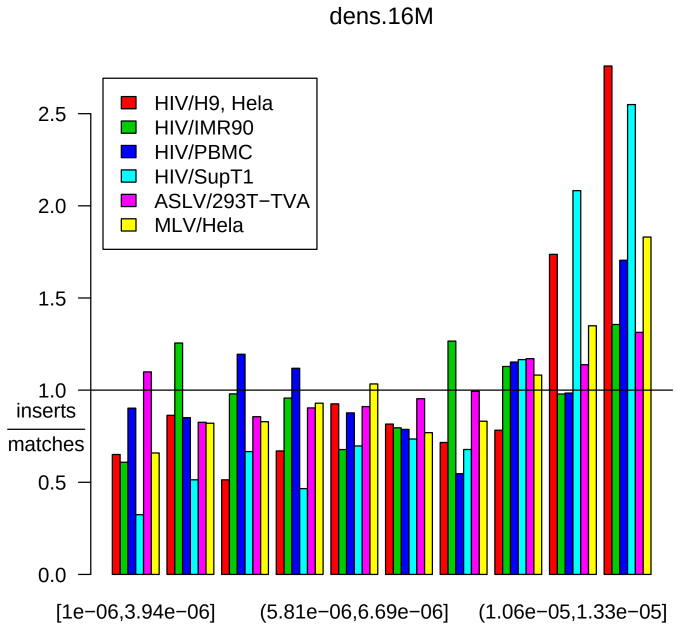


| | coef | se | z | p |
|---|---|---|---|---|
| HIV/H9, Hela | 0.5970 | 0.1070 | 5.590 | 2.30e-08 |
| HIV/IMR90 | 0.2210 | 0.0983 | 2.240 | 2.48e-02 |
| HIV/PBMC | 0.0394 | 0.0913 | 0.431 | 6.66e-01 |
| HIV/SupT1 | 1.0200 | 0.1130 | 9.010 | 2.12e-19 |
| ASLV/293T-TVA | 0.1800 | 0.0974 | 1.850 | 6.46e-02 |
| MLV/Hela | 0.3100 | 0.0741 | 4.180 | 2.90e-05 |

Here are the results for EST density. First, we count just one EST per gene.

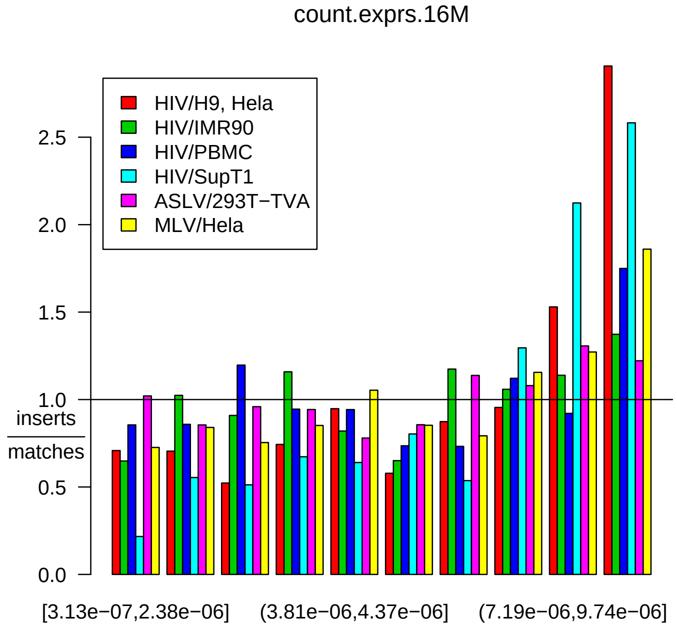


| | coef | se | z | p |
|---|---|---|---|---|
| HIV/H9, Hela | 0.603 | 0.1070 | 5.620 | 1.95e-08 |
| HIV/IMR90 | 0.174 | 0.0981 | 1.770 | 7.62e-02 |
| HIV/PBMC | 0.083 | 0.0916 | 0.906 | 3.65e-01 |
| HIV/SupT1 | 1.080 | 0.1150 | 9.420 | 4.52e-21 |
| ASLV/293T-TVA | 0.189 | 0.0971 | 1.950 | 5.15e-02 |
| MLV/Hela | 0.338 | 0.0744 | 4.550 | 5.44e-06 |

Now we count up to 200 ESTs per gene:

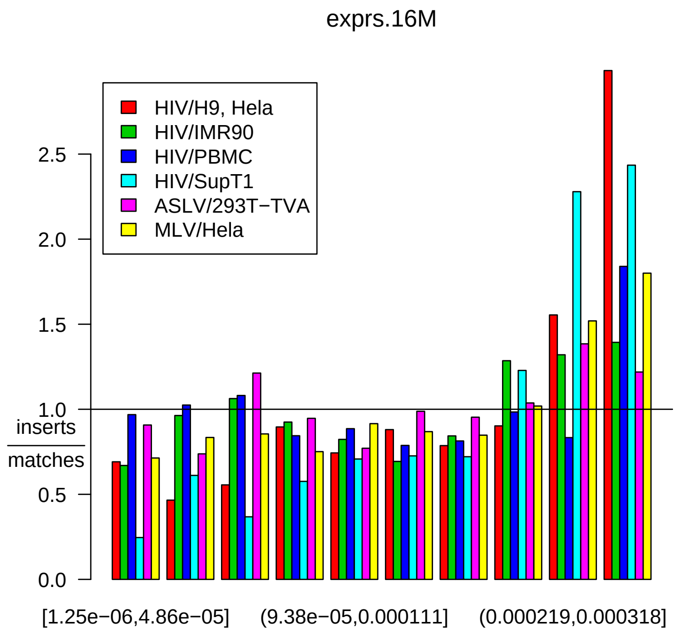


| | coef | se | z | p |
|---|---|---|---|---|
| HIV/H9, Hela | 0.7040 | 0.1090 | 6.460 | 1.07e-10 |
| HIV/IMR90 | 0.2240 | 0.0985 | 2.270 | 2.29e-02 |
| HIV/PBMC | 0.0778 | 0.0916 | 0.849 | 3.96e-01 |
| HIV/SupT1 | 1.1000 | 0.1160 | 9.520 | 1.76e-21 |
| ASLV/293T-TVA | 0.1860 | 0.0973 | 1.910 | 5.58e-02 |
| MLV/Hela | 0.3900 | 0.0745 | 5.240 | 1.63e-07 |

And here counting starts only after 200 ESTs per gene

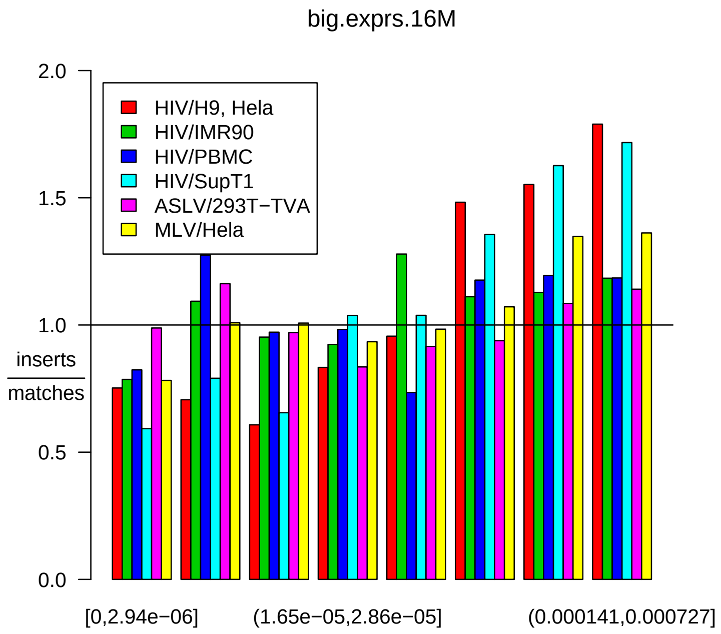


| | coef | se | z | p |
|---|---|---|---|---|
| HIV/H9, Hela | 0.6180 | 0.1080 | 5.750 | 8.93e-09 |
| HIV/IMR90 | 0.2540 | 0.0981 | 2.590 | 9.71e-03 |
| HIV/PBMC | 0.1130 | 0.0912 | 1.240 | 2.15e-01 |
| HIV/SupT1 | 0.7510 | 0.1070 | 7.040 | 1.92e-12 |
| ASLV/293T-TVA | -0.0421 | 0.0972 | -0.433 | 6.65e-01 |
| MLV/Hela | 0.2690 | 0.0742 | 3.620 | 2.96e-04 |

## 4.11 32 megaBase Window

First, we see gene density:

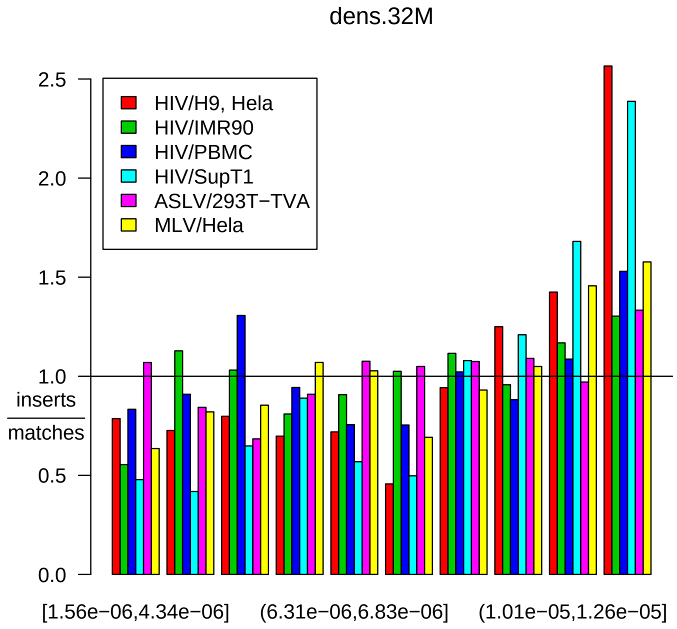


| | coef | se | z | p |
|---|---|---|---|---|
| HIV/H9, Hela | 0.5440 | 0.1070 | 5.08 | 3.76e-07 |
| HIV/IMR90 | 0.2370 | 0.0986 | 2.41 | 1.61e-02 |
| HIV/PBMC | 0.0978 | 0.0914 | 1.07 | 2.85e-01 |
| HIV/SupT1 | 0.8460 | 0.1100 | 7.71 | 1.29e-14 |
| ASLV/293T-TVA | 0.1740 | 0.0968 | 1.80 | 7.20e-02 |
| MLV/Hela | 0.2500 | 0.0738 | 3.38 | 7.15e-04 |

Here are the results for EST density. First, we count just one EST per gene.

count.exprs.32M

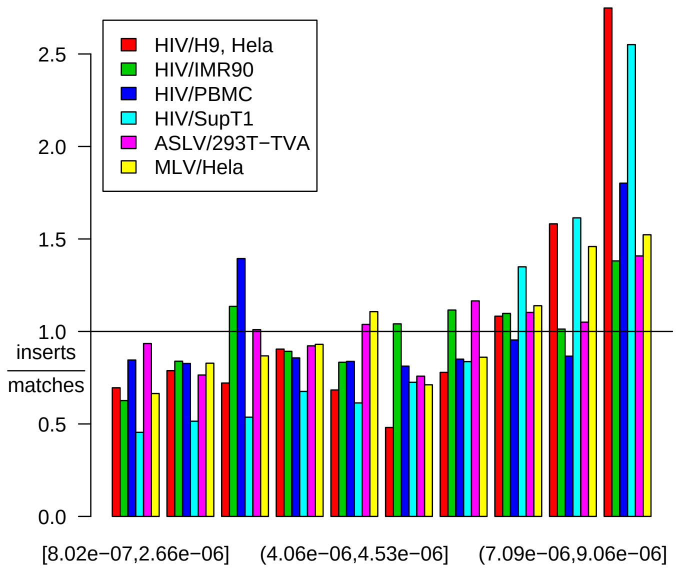


| | coef | se | z | p |
|---|---|---|---|---|
| HIV/H9, Hela | 0.5300 | 0.1060 | 4.980 | 6.33e-07 |
| HIV/IMR90 | 0.2690 | 0.0981 | 2.740 | 6.14e-03 |
| HIV/PBMC | 0.0901 | 0.0913 | 0.987 | 3.24e-01 |
| HIV/SupT1 | 0.9470 | 0.1120 | 8.480 | 2.25e-17 |
| ASLV/293T-TVA | 0.1440 | 0.0971 | 1.480 | 1.39e-01 |
| MLV/Hela | 0.2500 | 0.0736 | 3.400 | 6.75e-04 |

Now we count up to 200 ESTs per gene:

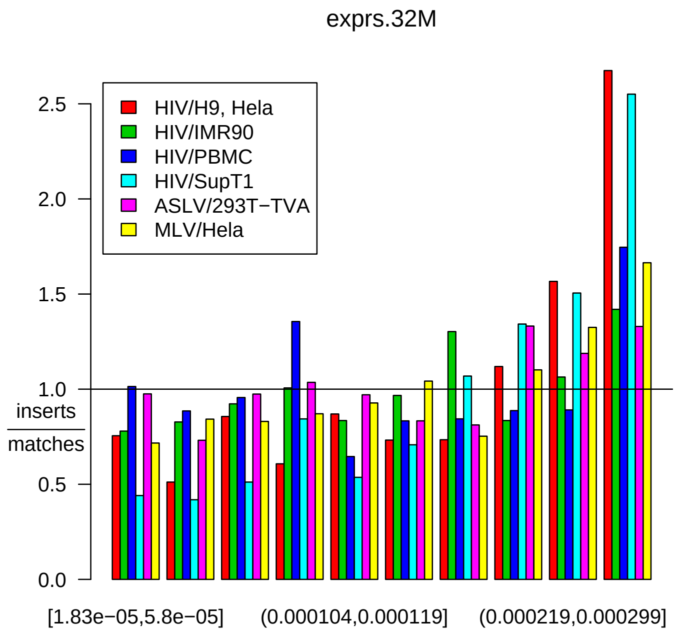


| | coef | se | z | p |
|---|---|---|---|---|
| HIV/H9, Hela | 0.5980 | 0.1070 | 5.570 | 2.58e-08 |
| HIV/IMR90 | 0.2490 | 0.0983 | 2.530 | 1.14e-02 |
| HIV/PBMC | 0.0592 | 0.0914 | 0.648 | 5.17e-01 |
| HIV/SupT1 | 0.9800 | 0.1120 | 8.720 | 2.71e-18 |
| ASLV/293T-TVA | 0.1440 | 0.0968 | 1.490 | 1.36e-01 |
| MLV/Hela | 0.3360 | 0.0740 | 4.530 | 5.78e-06 |

And here counting starts only after 200 ESTs per gene

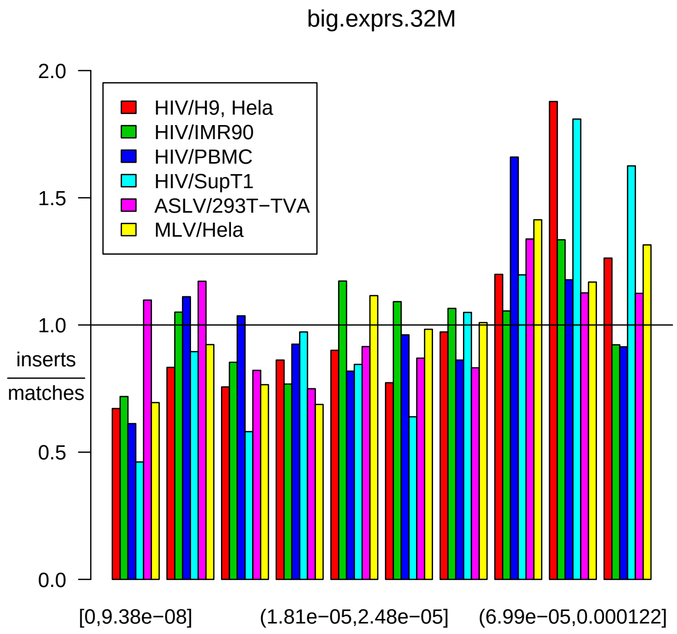


| | coef | se | z | p |
|---|---|---|---|---|
| HIV/H9, Hela | 0.4040 | 0.1060 | 3.830 | 1.28e-04 |
| HIV/IMR90 | 0.1810 | 0.0975 | 1.860 | 6.36e-02 |
| HIV/PBMC | 0.2090 | 0.0914 | 2.290 | 2.22e-02 |
| HIV/SupT1 | 0.5070 | 0.1040 | 4.870 | 1.12e-06 |
| ASLV/293T-TVA | 0.0926 | 0.0971 | 0.954 | 3.40e-01 |
| MLV/Hela | 0.3460 | 0.0745 | 4.640 | 3.43e-06 |

# 5 Juxtaposition with Gene Start and End Positions

## 5.1 Acembly Annotations

In this section we study the effect of juxtaposition in terms of gene start and end positions. The first barplot shows the effect of gene width for those insertions that are located within an Acembly gene. The table following the barplot shows the p-values for a test of the hypothesis that the proportions in each of the categories that define the bars are equal in the insertions and their matches. This p-value is obtained from the $5 \times 2 \times k$ table of counts defined by gene width category, insertion/match status, and stratum (consisting of an insertion and its matched sites) using a likelihood ratio test for the hypothesis of no

association between gene width category and insertion/match status. The test used compared the log-linear model [1] with all two-way configurations to that with no gene width category and insertion/match status configuration.

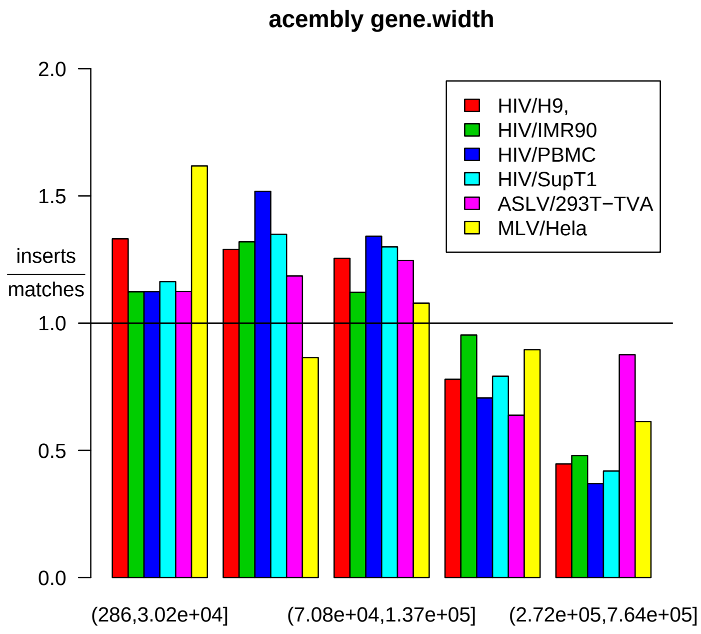


```
 HIV/H9,      HIV/IMR90       HIV/PBMC      HIV/SupT1 ASLV/293T-TVA
6.08e-09       7.40e-07       1.70e-18       1.46e-10      1.96e-04
MLV/Hela
1.12e-11
```

The next plot uses the width of a non-gene region for insertions that fall into such regions.

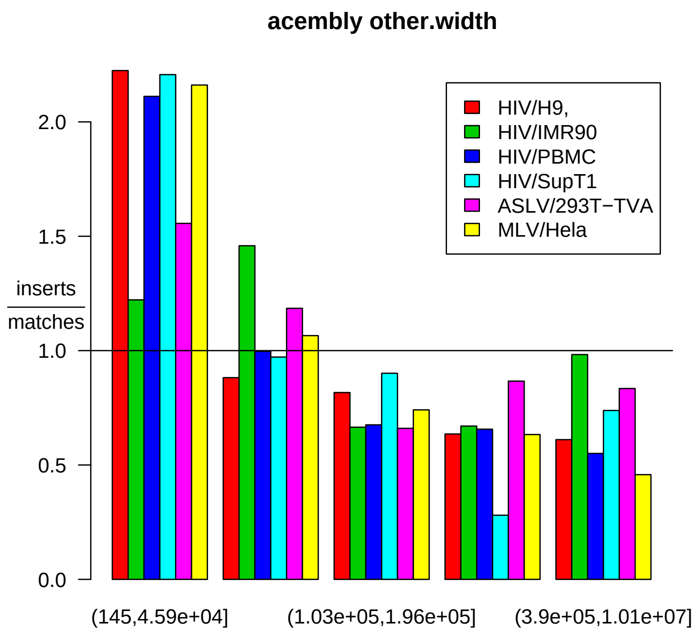


| HIV/H9, | HIV/IMR90 | HIV/PBMC | HIV/SupT1 | ASLV/293T-TVA |
|---|---|---|---|---|
| 3.02e-06 | 3.98e-02 | 6.65e-04 | 1.40e-06 | 8.95e-04 |
| MLV/Hela | | | | |
| 3.03e-22 | | | | |

The next plot studies the distance to the nearest boundary between a gene and a non-gene region. The distance is expressed as a fraction of the length of the region. Thus, '0.25' refers to one quarter of the distance from the site to nearest boundary divided by the total width of the region.

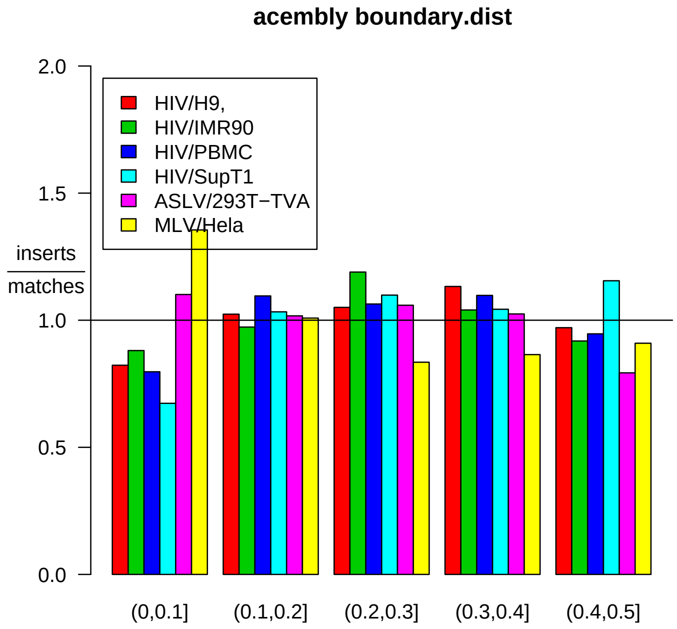


| HIV/H9, | HIV/IMR90 | HIV/PBMC | HIV/SupT1 | ASLV/293T-TVA |
|---|---|---|---|---|
| 3.52e-01 | 2.64e-01 | 1.26e-01 | 8.40e-03 | 2.27e-01 |
| MLV/Hela | | | | |
| 9.57e-06 | | | | |

This plot studies the effect of nearness to the beginning of a transcript. For sites in genes, it is the distance to the start of the gene divided by the width of the gene. For other sites it is the distance from the site to the nearer gene if that gene boundary is also a transcription starting point. Locations near '0' are relatively near the beginning of transcription, while those near '1' are near the termination of the transcript.

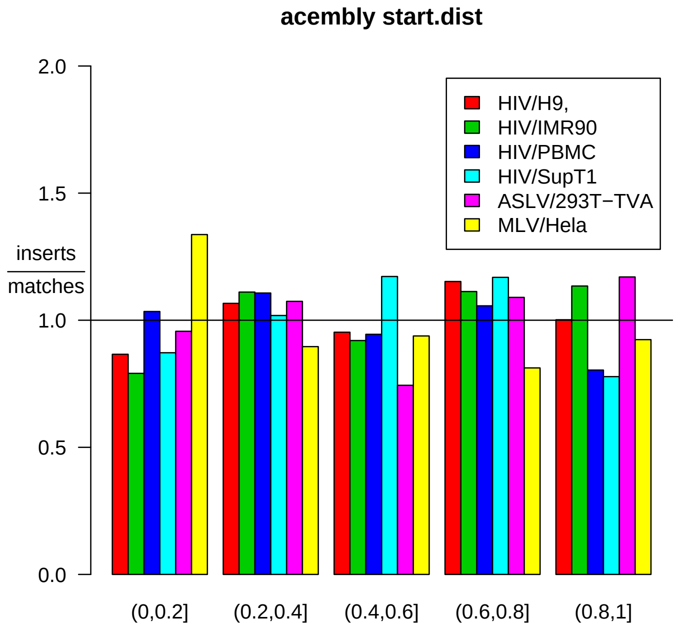


| HIV/H9, | HIV/IMR90 | HIV/PBMC | HIV/SupT1 | ASLV/293T-TVA |
|---|---|---|---|---|
| 4.67e-01 | 8.12e-02 | 2.11e-01 | 6.56e-02 | 4.62e-02 |
| MLV/Hela | | | | |
| 3.91e-05 | | | | |

## 5.2 RefSeq Annotations

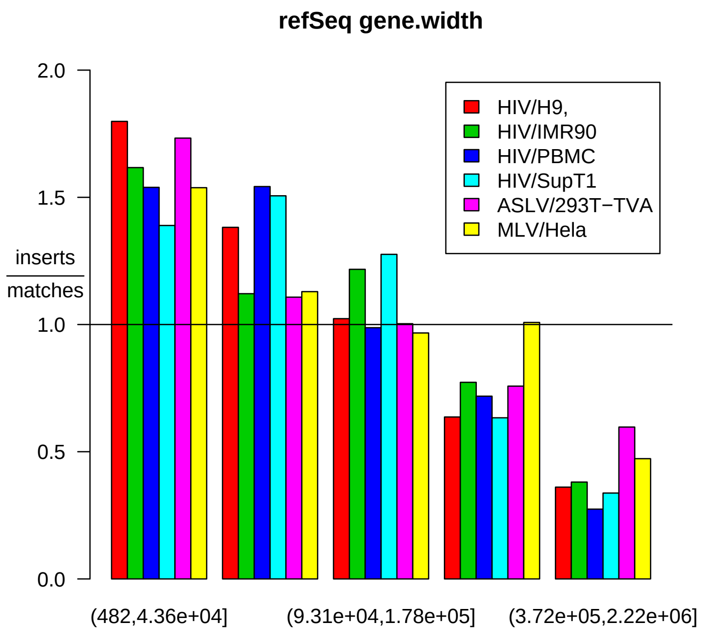


```
 HIV/H9,     HIV/IMR90      HIV/PBMC     HIV/SupT1 ASLV/293T-TVA
4.25e-12      6.00e-10      1.45e-18      7.99e-14      5.00e-04
MLV/Hela
9.57e-06
```

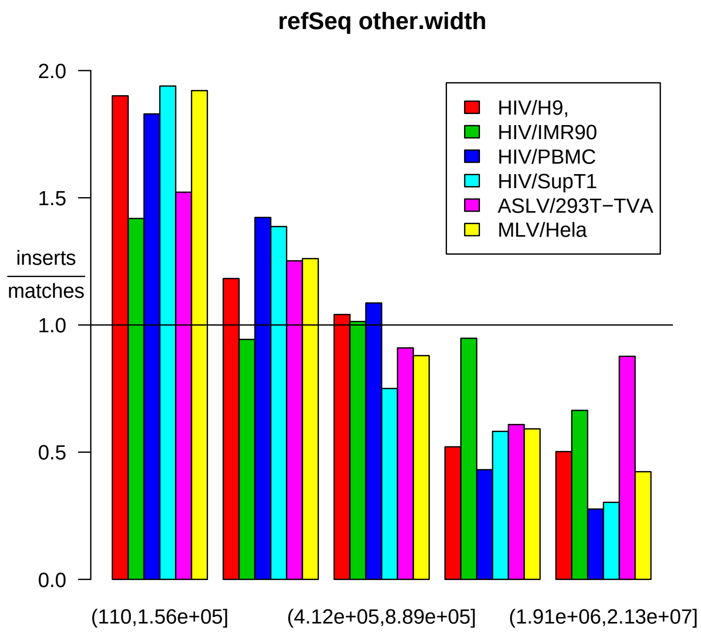


```
 HIV/H9,     HIV/IMR90      HIV/PBMC     HIV/SupT1 ASLV/293T-TVA
5.82e-10      6.57e-02      4.07e-15      6.01e-14      5.13e-05
MLV/Hela
3.10e-30
```

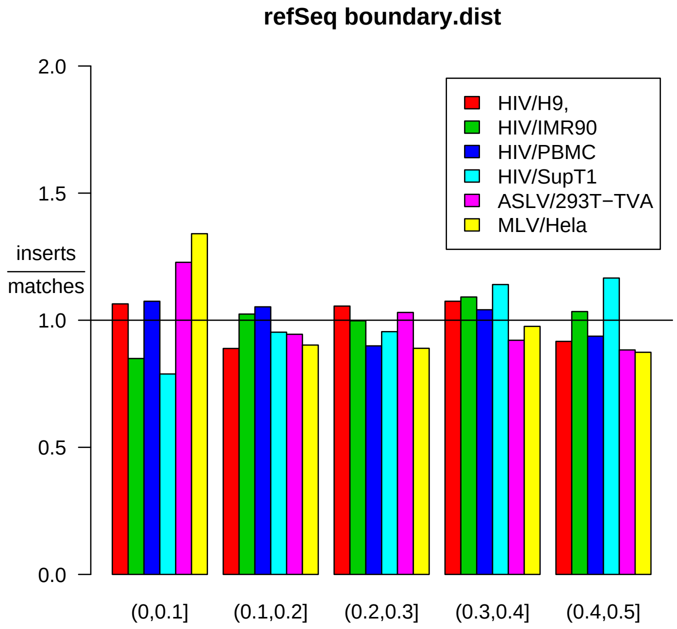


| HIV/H9, | HIV/IMR90 | HIV/PBMC | HIV/SupT1 | ASLV/293T-TVA |
|---|---|---|---|---|
| 6.10e-01 | 5.14e-01 | 6.46e-01 | 8.80e-02 | 1.51e-01 |
| MLV/Hela | | | | |
| 5.83e-05 | | | | |

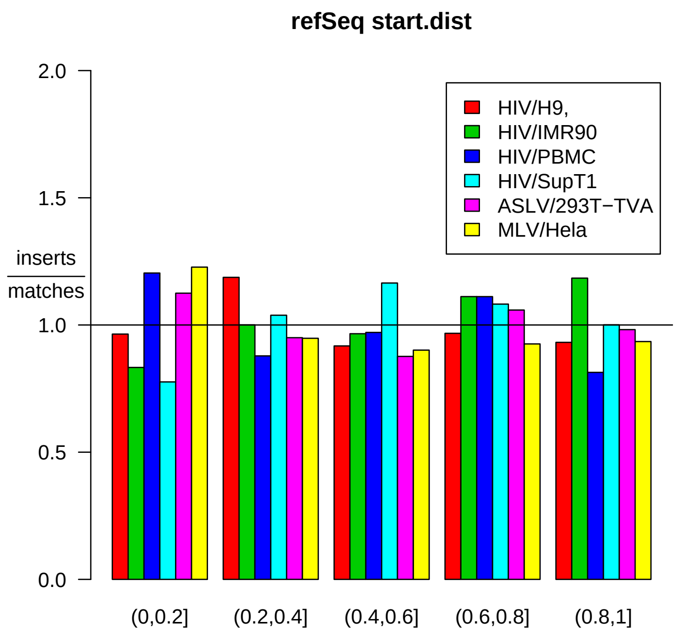


| HIV/H9, | HIV/IMR90 | HIV/PBMC | HIV/SupT1 | ASLV/293T-TVA |
|---|---|---|---|---|
| 0.5280 | 0.3020 | 0.0324 | 0.1000 | 0.5210 |

| MLV/Hela |
|---|
| 0.0400 |

## 5.3 genScan Annotations

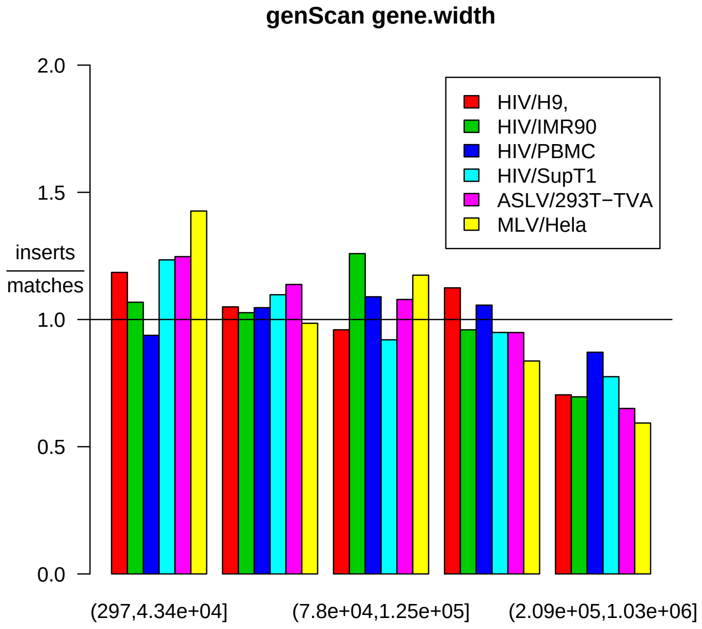


```
 HIV/H9,     HIV/IMR90      HIV/PBMC     HIV/SupT1 ASLV/293T-TVA
4.90e-02      2.75e-02      4.42e-01      7.39e-02      3.23e-03
MLV/Hela
1.95e-08
```

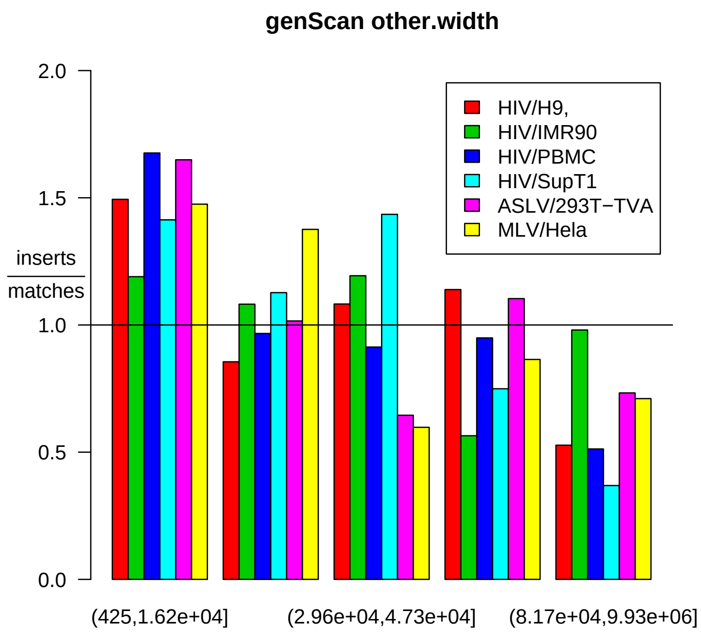


| HIV/H9, | HIV/IMR90 | HIV/PBMC | HIV/SupT1 | ASLV/293T-TVA |
|---|---|---|---|---|
| 9.43e-02 | 1.21e-01 | 5.59e-02 | 3.70e-04 | 4.23e-03 |
| MLV/Hela | | | | |
| 2.88e-09 | | | | |

### genScan boundary.dist

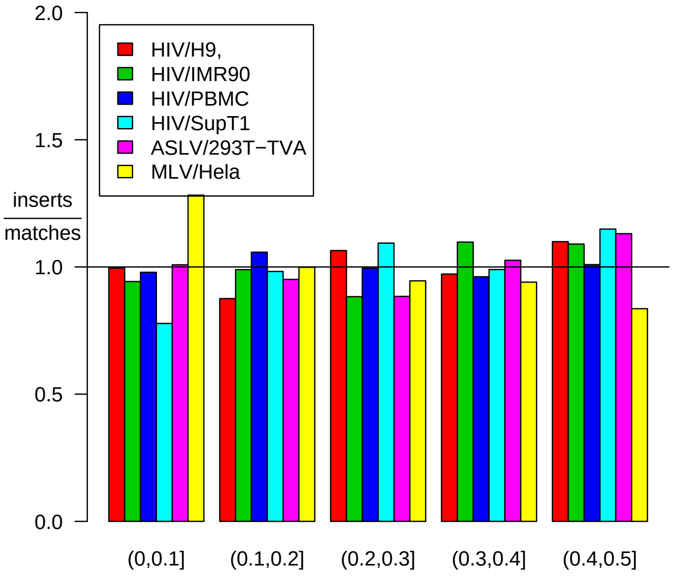


| HIV/H9, | HIV/IMR90 | HIV/PBMC | HIV/SupT1 | ASLV/293T-TVA |
|---|---|---|---|---|
| 0.64100 | 0.51500 | 0.96900 | 0.13100 | 0.54000 |
| MLV/Hela | | | | |
| 0.00135 | | | | |

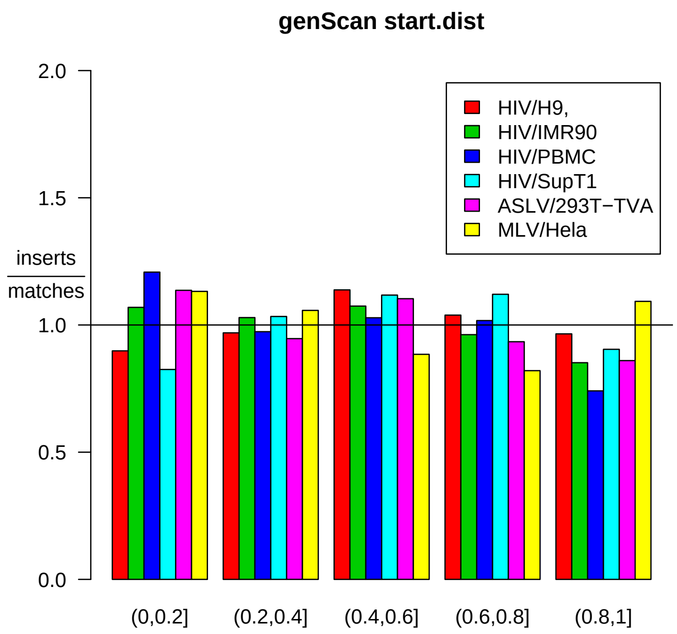


| HIV/H9, | HIV/IMR90 | HIV/PBMC | HIV/SupT1 | ASLV/293T-TVA |
|---|---|---|---|---|
| 0.7720 | 0.5790 | 0.0311 | 0.2780 | 0.3340 |

| MLV/Hela |
|---|
| 0.0221 |

## 5.4 uniGene Annotations

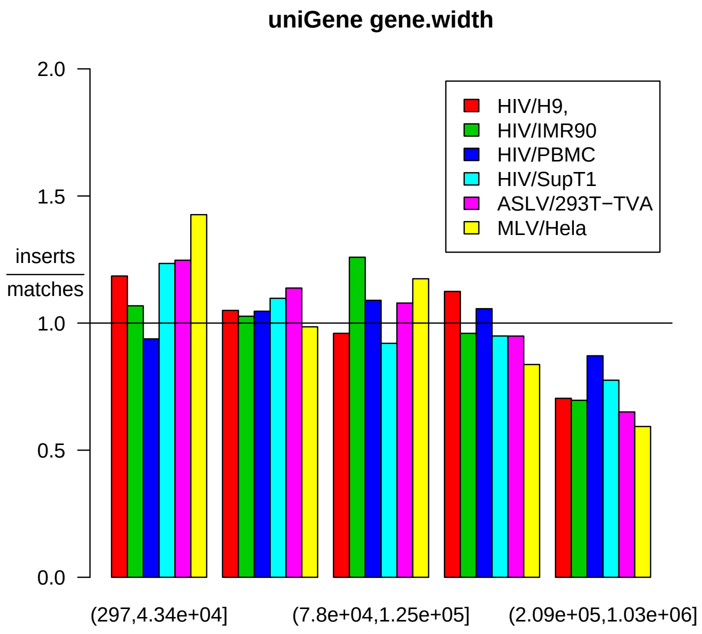


```
 HIV/H9,     HIV/IMR90      HIV/PBMC      HIV/SupT1 ASLV/293T-TVA
4.90e-02      2.75e-02      4.42e-01       7.39e-02      3.23e-03
MLV/Hela
1.95e-08
```

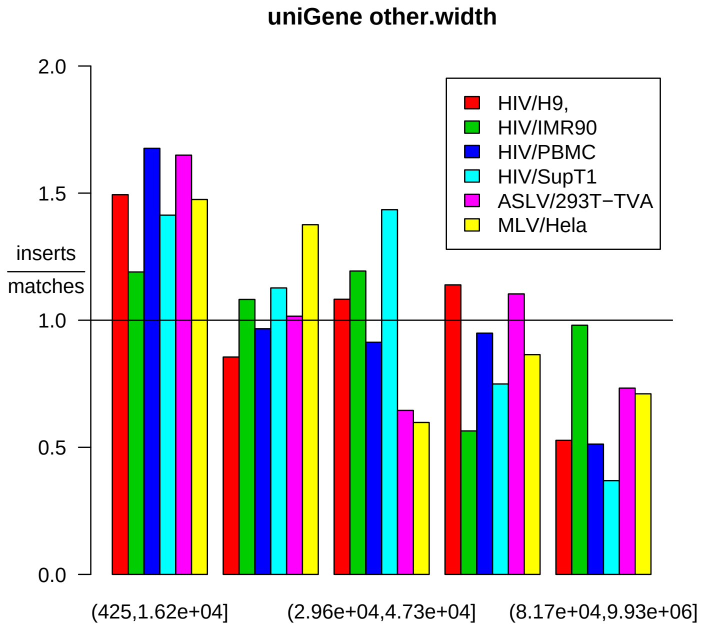


| HIV/H9, | HIV/IMR90 | HIV/PBMC | HIV/SupT1 | ASLV/293T-TVA |
|---|---|---|---|---|
| 9.43e-02 | 1.21e-01 | 5.59e-02 | 3.70e-04 | 4.23e-03 |
| MLV/Hela | | | | |
| 2.88e-09 | | | | |

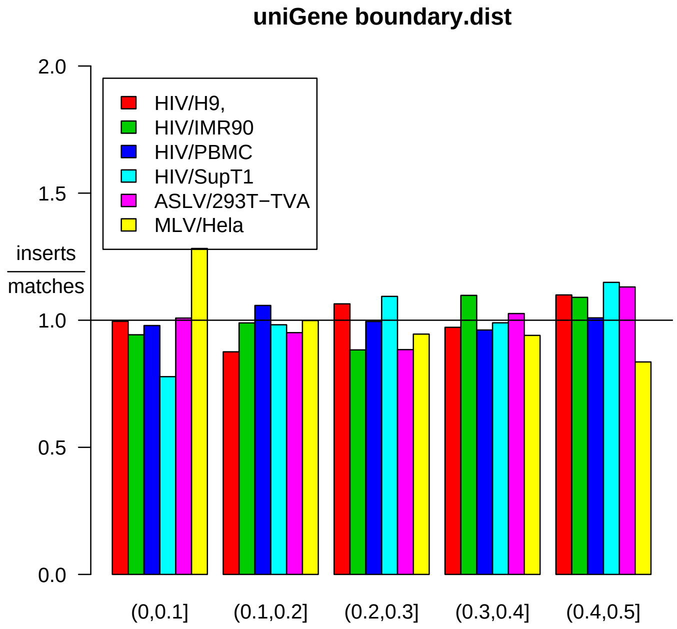


| HIV/H9, | HIV/IMR90 | HIV/PBMC | HIV/SupT1 | ASLV/293T-TVA |
|---|---|---|---|---|
| 0.64100 | 0.51500 | 0.96900 | 0.13100 | 0.54000 |

| MLV/Hela |
|---|
| 0.00135 |

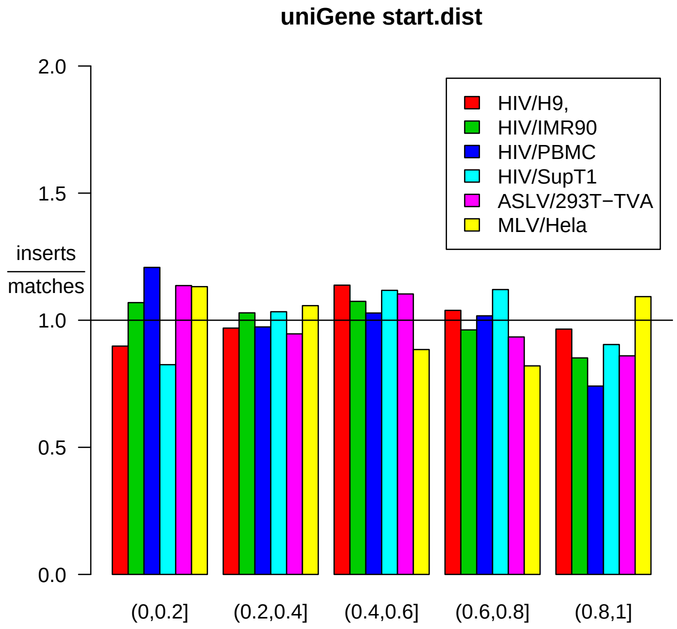


```
     HIV/H9,       HIV/IMR90        HIV/PBMC       HIV/SupT1 ASLV/293T-TVA
      0.7720          0.5790          0.0311          0.2780        0.3340
    MLV/Hela
      0.0221
```

# 6 GC content

Here we study the effect of GC content on insertion. The GC content is taken from the Human Genome Draft at GoldenPath from the table `http://genome.ucsc.edu/goldenPath/14nov2002/database/gcPercent.txt.gz`.

Following the plot is a table of fitted coefficients based on splitting the GC percent data at the median.

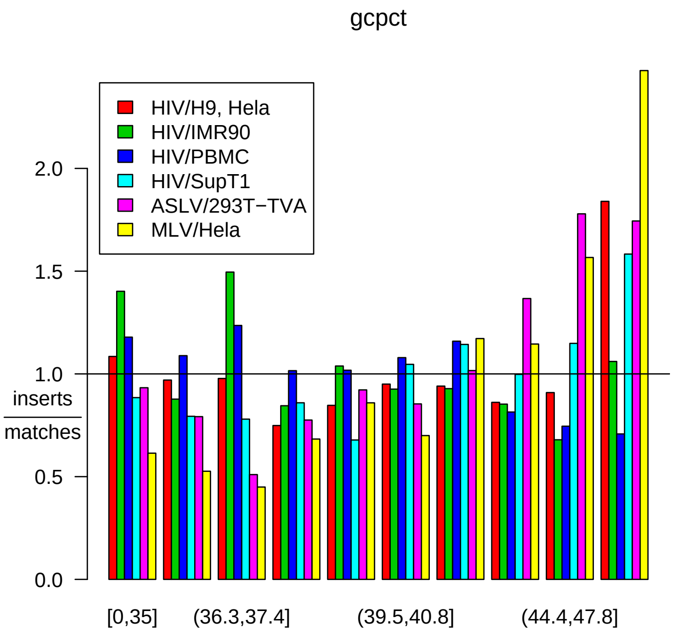


| | coef | se | z | p |
|---|---|---|---|---|
| HIV/H9, Hela | 0.147 | 0.1050 | 1.40 | 1.61e-01 |
| HIV/IMR90 | -0.223 | 0.0977 | -2.28 | 2.24e-02 |
| HIV/PBMC | -0.207 | 0.0913 | -2.26 | 2.35e-02 |
| HIV/SupT1 | 0.406 | 0.1030 | 3.95 | 7.84e-05 |
| ASLV/293T-TVA | 0.493 | 0.0976 | 5.05 | 4.52e-07 |
| MLV/Hela | 0.819 | 0.0791 | 10.40 | 4.01e-25 |

# 7 Cytobands

Here we study the association of cytoband with insertion intensity. The data are obtained from
`http://genome.ucsc.edu/goldenPath/14nov2002/database/cytoBand.txt.gz`.

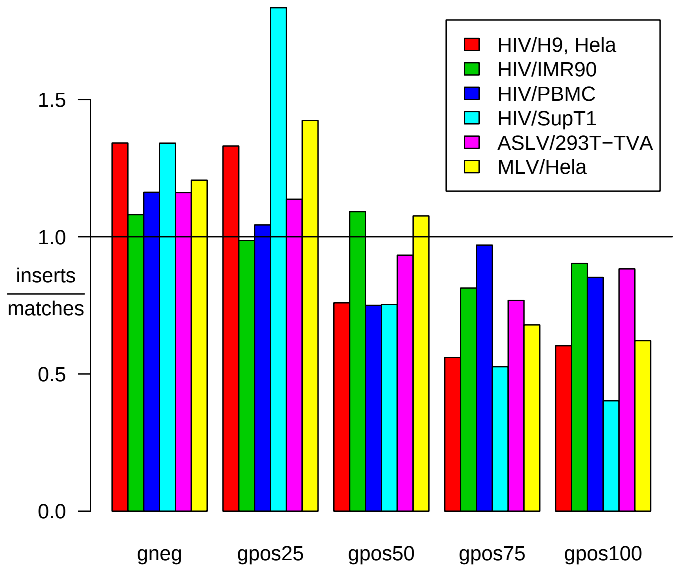


A formal test of significance attains a p-value of $< 2.22e - 16$. Here is the table of coefficients of the log ratio of intensities for true insertion sites versus control insertion sites (comparing each category of Giemsa staining to 'gneg') along with their standard errors, z statistics, and p-values:

```
                    coef     se     z        p
cyto.typegpos100 -0.5370 0.0582 -9.22 2.94e-20
cyto.typegpos25   0.0704 0.0675  1.04 2.97e-01
cyto.typegpos50  -0.2820 0.0579 -4.87 1.13e-06
cyto.typegpos75  -0.5140 0.0620 -8.29 1.14e-16
```

# References

[1] Yvonne M. M. Bishop, Stephen E. Fienberg, and Paul W. Holland. *Discrete multivariate analyses: Theory and practice.* MIT Press, 1975.

[2] P. McCullagh and John A. Nelder. *Generalized linear models.* Chapman & Hall Ltd, 1999.

[3] Rogier. Versteeg, Barbera. D. C. van Schaik., Marinus. F. van Batenburg., Marco. Roos, Ramin. Monajemi, Huib. Caron, Harmen. J. Bussemaker, and Antoine. H. C. van Kampen. The human transcriptome map reveals extremes in gene density, intron length, GC content, and repeat pattern for

domains of highly and weakly expressed genes. *Genome Res*, 13(9):1998–2004, Sep 2003.

[4] Xiaolin. Wu, Yuan. Li, Bruce. Crise, and Shawn. M. Burgess. Transcription start regions in the human genome are favored targets for MLV integration. *Science*, 300(5626):1749–1751, Jun 2003.
