# Supplementary material for: Retroviral DNA Integration: ASLV, HIV, and MLV Show Distinct Target Site Preferences
Source: PLoS Biol. 2004 Aug 17;2(8):e234. doi: 10.1371/journal.pbio.0020234 (PMC509299; doi:10.1371/journal.pbio.0020234)
Supplement: Protocol S2 — (128 KB PDF). [file pbio.0020234.sd002.pdf]

Association of Genomic Features with Integration - Part 2

Charles C. Berry

April 20, 2004

Contents

1 Loci in Genes and Exons **1**

2 Positioning in or near genes **3**

3 Gene Density **6**

4 Cytobands **9**

5 CpG Islands **9**

6 GC content **12**

7 Combined Effects **13**

Here we look into the associations in more detail - particularly we try to compare the differing insertion types. We do this using a conditional logit model in which features of each integration site are compared to those of a set of sites that have been sampled from those sites on the genome that are the same distance from the nearest restriction site as the integration site (in the direction in which the sequence is read).

1 Loci in Genes and Exons

The following analysis of deviance table [1] shows the goodness of fit and sequential significance tests of several nested models.

```
Analysis of Deviance Table

Model 1 : NULL
Model 2 : In Gene
Model 3 : Data Set : In Gene
Model 4 : Data Set : In Gene + In Exon
```

```
Model 5 : Data Set : In Gene + Data Set : In Exon
  Resid. Df Resid. Dev    Df Deviance  P(>|Chi|)
1     34397    14996.4
2     34396    14289.5     1    706.9 9.280e-156
3     34391    14143.6     5    145.9  1.009e-29
4     34390    14112.7     1     30.9  2.669e-08
5     34385    14104.0     5      8.6        0.1
```

The first model is a baseline with no terms in it. The second model has only a single term indicating whether an insertion or a matching site is in an Acembly gene. As is evident by the substantial decrement in the deviance (and the associated small p-value from the likelihood ratio test) due to this one term, being in a gene has a marked effect on integration. The intensity for integration is 2.84 times as great at a locus in a gene as at a locus that is not in a gene. The third model allows for differences among the effects of being in a gene in the six data sets and it is apparent that there are differences. To get some further detail on the differences among the data sets with respect to the effect of being in a gene, pairwise comparisons among the data sets are performed (using Wald tests). These are summarized in the following table:

```
                                 stat df    p.value log.ratio
ASLV/293T-TVA vs HIV/H9, Hela  39.53  1 3.2279e-10     -1.00
ASLV/293T-TVA vs HIV/IMR90     25.39  1 4.6945e-07     -0.75
ASLV/293T-TVA vs HIV/PBMC      73.33  1 < 2.22e-16     -1.35
ASLV/293T-TVA vs HIV/SupT1     43.07  1 5.2817e-11     -1.04
ASLV/293T-TVA vs MLV/Hela       0.81  1 0.36869868     -0.11
HIV/H9, Hela  vs HIV/IMR90      2.19  1 0.13928517      0.25
HIV/H9, Hela  vs HIV/PBMC       4.05  1 0.04422166     -0.35
HIV/H9, Hela  vs HIV/SupT1      0.05  1 0.82042347     -0.04
HIV/H9, Hela  vs MLV/Hela      37.12  1 1.1083e-09      0.89
HIV/IMR90     vs HIV/PBMC      13.00  1 0.00031217     -0.60
HIV/IMR90     vs HIV/SupT1      2.97  1 0.08501995     -0.29
HIV/IMR90     vs MLV/Hela      22.49  1 2.1146e-06      0.64
HIV/PBMC      vs HIV/SupT1      3.20  1 0.07347430      0.31
HIV/PBMC      vs MLV/Hela      73.61  1 < 2.22e-16      1.24
HIV/SupT1     vs MLV/Hela      40.89  1 1.6068e-10      0.93
```

The 'log.ratio' column gives the logarithm of the ratio of integration intensity in the first data set divided by that in the second listed data set. As is evident, the loci in genes in the 'ASLV' data are not as relatively attractive as integration sites as are loci in genes in the other data sets.

Model 4 adds a term for whether an insertion or a matching site is in an Acembly exon, which results in a statistically significant decrement in the deviance. The intensity for integration is 1.486 times as great at a locus in a gene as at a locus that is not in an exon.

Finally, Model 5 allows for differences among the effects of being in an exon in the six data sets, but no differences are apparent.

2 Positioning in or near genes

In this section we examine whether the position of a locus relative to a start of the coding region of a gene influences integration. We begin with a model that uses 'feature width' — the distance from the last boundary of a gene and the next one. This quantity is studied since it forms the denominator of the 'distance to start' measure, which gives the fraction of distance from the coding start site to the insertion site for insertions that are in genes or for insertions that are not in genes the distance to the nearest gene if that gene is transcribed in the direction leading away from the insertion. Here is the analysis of deviance table comparing the null model that allows for regions in genes to differ according to the data set from which they came to a model that adds log(feature distance) to another model that allows the log(feature distance) terms to vary according to data set:

```
Analysis of Deviance Table

Model 1 : Data Set : In Gene
Model 2 : Data Set : In Gene + log( feature width )
Model 3 : Data Set : In Gene + Data Set : log( feature width )
  Resid. Df Resid. Dev    Df Deviance P(>|Chi|)
1     34391    14143.6
2     34390    13882.4     1    261.2 9.452e-59
3     34385    13867.0     5     15.4 8.646e-03
```

As is evident, most of the improvement in model fit is achieved in passing from model 1 to model 2. A similar picture is obtained by using a somewhat richer model for feature width, viz. that which uses B-splines for log(feature distance) with two interior knots. Here is the analogous analysis of deviance table:

```
Analysis of Deviance Table

Model 1 : Data Set : In Gene
Model 2 : Data Set : In Gene + bs( log( feature width ), df=5)
Model 3 : Data Set : In Gene + Data Set : bs( log( feature width ), df=5)
  Resid. Df Resid. Dev    Df Deviance P(>|Chi|)
1     34391    14143.6
2     34386    13819.6     5    324.1 6.644e-68
3     34361    13747.0    25     72.6 1.593e-06
```

The next table considers the distance from/to the start of transcription. This distance is the fraction of distance from the coding start site to the insertion site divided by the length of the gene for insertions that are in genes. For insertions that are not in genes it is the distance to the nearest gene if that gene is transcribed in the direction leading away from the insertion divided by the distance between genes. Otherwise the distance to the nearest gene divided by the distance between genes is used.

```
Analysis of Deviance Table

Model 1 : Data Set : In Gene
Model 2 : Data Set : In Gene + start distance
Model 3 : Data Set : In Gene + Data Set : start distance
  Resid. Df Resid. Dev    Df Deviance P(>|Chi|)
1     34391    14143.6
2     34390    14130.1     1     13.5 2.341e-04
3     34385    14086.5     5     43.6 2.752e-08
```

As is evident, there are statistically significant reductions in the deviance in each step. The following table uses B-splines with two interior knots for start distance:

```
Analysis of Deviance Table

Model 1 : Data Set : In Gene
Model 2 : Data Set : In Gene + bs( start distance , df=5)
Model 3 : Data Set : In Gene + Data Set : bs( start distance, df=5)
  Resid. Df Resid. Dev    Df Deviance P(>|Chi|)
1     34391    14143.6
2     34386    14125.3     5     18.4 2.529e-03
3     34361    14029.4    25     95.8 3.112e-10
```

Again it is evident that there are statistically significant reductions in the deviance in each step. Thus, the distance from (or to) the start site affects the integration intensity and does so differently in the different data sets. Here are the pairwise comparisons between the data sets for the start distance.

```
                                 stat df    p.value
ASLV/293T-TVA vs HIV/H9, Hela    7.43  5 0.19056884
ASLV/293T-TVA vs HIV/IMR90       8.04  5 0.15427234
ASLV/293T-TVA vs HIV/PBMC        7.02  5 0.21936817
ASLV/293T-TVA vs HIV/SupT1      14.46  5 0.01296165
ASLV/293T-TVA vs MLV/Hela       15.36  5 0.00894216
HIV/H9, Hela  vs HIV/IMR90      10.80  5 0.05543477
HIV/H9, Hela  vs HIV/PBMC        1.58  5 0.90417134
HIV/H9, Hela  vs HIV/SupT1       4.39  5 0.49442193
HIV/H9, Hela  vs MLV/Hela       21.95  5 0.00053573
HIV/IMR90     vs HIV/PBMC        9.90  5 0.07802187
HIV/IMR90     vs HIV/SupT1       8.31  5 0.14017238
HIV/IMR90     vs MLV/Hela       39.26  5 2.1094e-07
HIV/PBMC      vs HIV/SupT1       4.22  5 0.51871337
HIV/PBMC      vs MLV/Hela       26.39  5 7.4869e-05
HIV/SupT1     vs MLV/Hela       33.72  5 2.7076e-06
```

Note that all of the comparisons with MLV are statistically significant, three of the ASLV comparisons are statistically significant, and none of the other pair-

wise comparisons are statistically significant. However, it is worth noting that the actual reduction in deviance is generally small; in part this is a consequence of there being little influence on integration in most of the data sets.

Since it is of interest to determine whether ASLV shares the preference of MLV for integrating into the 5' end of genes, we examine the empirical distribution of integration sites by forming a barplot for each data set in which the relative intensity of integration is plotted for 10 intervals of 'start distance':

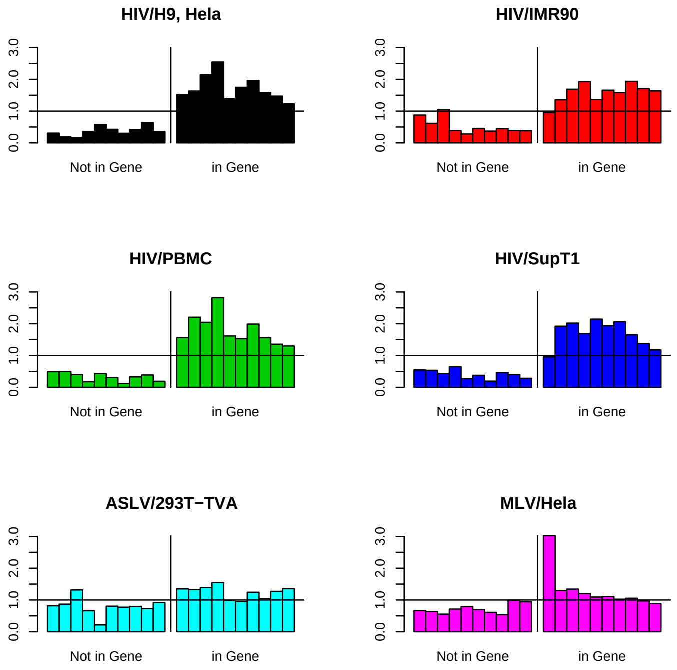

It appears that the integration intensity in the 5' end of a gene is somewhat elevated in the ASLV data, but not by nearly so much as in the MLV data. We test this directly by fitting a model that includes an indicator variable for whether a site is in a gene and with a 'start distance' of less than 0.1 for each data set. Here is a table of results for this model. The 'se' column gives the standard error of the logarithm of the relative intensity for integration.

	relative intensity	se	z	p.value
HIV/H9, Hela	1.564	0.201	2.231	2.566917e-02
HIV/IMR90	0.948	0.234	-0.229	8.187599e-01
HIV/PBMC	1.608	0.178	2.667	7.653736e-03
HIV/SupT1	0.955	0.240	-0.193	8.470914e-01
ASLV/293T-TVA	1.373	0.204	1.551	1.209196e-01
MLV/Hela	3.323	0.113	10.651	1.732832e-26

The effect for ASLV is not less than the conventional 0.05 significance level, and the preference for integration near the 5' end of a gene appears markedly lower than that for MLV. Here are the pairwise comparisons:

	stat	df	p.value	log.ratio
ASLV/293T-TVA vs HIV/H9, Hela	0.21	1	0.64850205	-0.13
ASLV/293T-TVA vs HIV/IMR90	1.42	1	0.23280706	0.37
ASLV/293T-TVA vs HIV/PBMC	0.34	1	0.56023887	-0.16
ASLV/293T-TVA vs HIV/SupT1	1.33	1	0.24904433	0.36
ASLV/293T-TVA vs MLV/Hela	14.35	1	0.00015203	-0.88
HIV/H9, Hela vs HIV/IMR90	2.65	1	0.10383989	0.50
HIV/H9, Hela vs HIV/PBMC	0.01	1	0.91875848	-0.03
HIV/H9, Hela vs HIV/SupT1	2.49	1	0.11428508	0.49
HIV/H9, Hela vs MLV/Hela	10.73	1	0.00105581	-0.75
HIV/IMR90 vs HIV/PBMC	3.23	1	0.07218298	-0.53
HIV/IMR90 vs HIV/SupT1	0.00	1	0.98253573	-0.01
HIV/IMR90 vs MLV/Hela	23.36	1	1.3451e-06	-1.25
HIV/PBMC vs HIV/SupT1	3.04	1	0.08106811	0.52
HIV/PBMC vs MLV/Hela	11.87	1	0.00056911	-0.73
HIV/SupT1 vs MLV/Hela	22.15	1	2.5208e-06	-1.25

3 Gene Density

In this section the effects of the local density of genes and of expressed genes is studied. The Ensemble genes and ESTs collection described in Vesteeg et al [2] are used. For every insertion site, a region around that site is searched for genes and for expressed genes (i.e. those having EST counts greater than zero). The local gene density is given by

$$d_{genes} = \frac{\text{Count of genes}}{\text{Width}}$$

Similarly, the expressed gene density is given as

$$d_{countexpressed} = \frac{\text{Count of expressed genes}}{\text{Width}}$$

, a score of expression density is also computed as

$$d_{score} = \frac{1}{width} \sum_{i:abs(position_{site}-position_i)<width/2} (min(200, EST_i))$$

i.e. the EST count is trimmed at 200 and the sum of all truncated counts in the region is divided its width. In addition a score for high EST counts is given by:

$$d_{high} = \frac{1}{width} \sum_{i:abs(position_{site}-position_i)<width/2} (max(0, EST_i - 200))$$

Note that $d_{score} + d_{high}$ is just the EST count divided by the region width. The motivation for decomposing it into several pieces is that the distribution of EST counts has a very long upper tail, and there was a suspicion that the impact of a single EST with a very high count would be much less than that of a number of ESTs whose total was equally high.

As it turns out, the resulting scores will often be zero and also have rather long upper tails. Preliminary analysis suggested forming a zero-one indicator variable to flag those site in which the score is zero and a quantitative variable that is either the logarithm of the score for non-zero scores. When the original score is zero, the variable has the median of those log scores in its place.

The following analysis of deviance table shows the effects of gene density in a 500 kilobase region surrounding each site. The null model has effects for the genes that differ according to data source, the next model has an indicator for zero score and the quantitative (log-score) variable included, and the final model allows the effects of the indicator and quantitative score to vary according to data source.

```
Analysis of Deviance Table

Model 1 : Data Set : In Gene
Model 2 : Data Set : In Gene + genes per 500k
Model 3 : Data Set : In Gene + Data Set : genes per 500k
  Resid. Df Resid. Dev    Df Deviance  P(>|Chi|)
1     34391    14143.6
2     34389    13659.3     2    484.3 6.866e-106
3     34379    13581.8    10     77.5  1.551e-12
```

As is evident, the bulk of the decrease in deviance is in the first step. Still the second step does attain statistical significance, indicating differences among the data sources with respect to the effect of gene density. Here is the analogous analysis of deviance table using B-splines with two interior knots for the quantitative scores.

```
Analysis of Deviance Table

Model 1 : Data Set : In Gene
Model 2 : Data Set : In Gene + bs( genes per 500k, df = 5 )
Model 3 : Data Set : In Gene + Data Set : bs( genes per 500k, df = 5 )
  Resid. Df Resid. Dev    Df Deviance  P(>|Chi|)
1     34391    14143.6
2     34385    13640.1     6    503.6 1.430e-105
3     34355    13530.2    30    109.9  4.793e-11
```

Again the bulk of the decrease in deviance is in the first step, although the second step is also statistically significant. Perhaps it is also worth noting that the use of the B-splines results in a modest improvement in the deviance over using the original score.

The following table gives the analysis of deviance for $d_{countexpressed}$:

```
Analysis of Deviance Table

Model 1 : Data Set : In Gene
Model 2 : Data Set : In Gene + expressed per 500k
Model 3 : Data Set : In Gene + Data Set : expressed per 500k
  Resid. Df Resid. Dev    Df Deviance  P(>|Chi|)
1     34391    14143.6
2     34389    13511.9     2    631.7 6.712e-138
3     34379    13425.7    10     86.2  2.983e-14
```

And here is the table for the B-spline with two interior knots for $d_{countexpressed}$

```
Analysis of Deviance Table

Model 1 : Data Set : In Gene
Model 2 : Data Set : In Gene + bs( expressed per 500k, df = 5 )
Model 3 : Data Set : In Gene + Data Set : bs( expressed per 500k, df = 5 )
  Resid. Df Resid. Dev    Df Deviance  P(>|Chi|)
1     34391    14143.6
2     34385    13491.5     6    652.1 1.328e-137
3     34355    13361.7    30    129.8  2.244e-14
```

Notice that the total decrement in deviance is substantially greater than that seen for gene density per se.

Here is the table for the expression score, d_{score}:

```
Analysis of Deviance Table

Model 1 : Data Set : In Gene
Model 2 : Data Set : In Gene + express score per 500k
Model 3 : Data Set : In Gene + Data Set : express score per 500k
  Resid. Df Resid. Dev    Df Deviance  P(>|Chi|)
1     34391    14143.6
2     34389    13488.1     2    655.5 4.528e-143
3     34379    13399.6    10     88.5  1.045e-14
```

And here is the analogous table using the Bspline with 2 interior knots:

```
Analysis of Deviance Table

Model 1 : Data Set : In Gene
Model 2 : Data Set : In Gene + bs( express score  per 500k, df = 5 )
Model 3 : Data Set : In Gene + Data Set : bs( express score per 500k, df = 5 )
  Resid. Df Resid. Dev    Df Deviance  P(>|Chi|)
1     34391    14143.6
2     34385    13461.4     6    682.3 4.133e-144
3     34355    13359.8    30    101.5  1.057e-09
```

4 Cytobands

Here the effect of being in a Gband is studied. The cytoband data is coded as 'Gscore', which assigns the values 0, 0.25, 0.5, 0.75, and 1.00 to the codes 'gneg', 'gpos25', 'gpos50', 'gpos75', and 'gpos100'. The analysis of deviance table shows that the incremental effect of accounting for 'Gscore' after taking account of whether an insertion is in a gene or an 'expression dense' region is statistically significant and that the Gscore effect varies in the different data sets. However, the magnitude of the decrease in deviance is rather small, which suggests that the effects are modest.

The point of departure is a model that includes the effect of expression 'density' and data set specific effects of being in a gene:

```
Analysis of Deviance Table

Model 1 : Data Set : In Gene + express score per 500k
Model 2 : Data Set : In Gene + express score per 500k + Gscore
Model 3 : Data Set : In Gene + Data Set : express score per 500k + Data Set : Gscore
  Resid. Df Resid. Dev    Df Deviance P(>|Chi|)
1     18646     4655.3
2     18645     4649.2     1      6.1 0.0135255
3     18640     4627.0     5     22.2 0.0004771
```

5 CpG Islands

Wu et al [3] noted an eight-fold difference in insertion in regions within ±1kb of CpG Islands. Using the annotated locations of the CpG Islands from `http://genome.ucsc.edu/goldenPath/14nov2002/database/cpgIsland.txt.gz` we determined whether the insertion site was within ±1kb, within ±5kb, or within ±10kb.

Here is the analysis of deviance table for regions within 1 kilobase of a CpG island (or in the island). The point of departure is a model that includes the effect of expression 'density' and data set specific effects of being in a gene:

```
Analysis of Deviance Table

Model 1 : Data Set : In Gene + express score per 500k
Model 2 : Data Set : In Gene + express score per 500k + CpG.1k
Model 3 : Data Set : In Gene + Data Set : express score per 500k + Data Set : CpG.1k
  Resid. Df Resid. Dev    Df Deviance P(>|Chi|)
1     34389    13488.1
2     34388    13450.2     1     37.9 7.363e-10
3     34383    13222.2     5    228.0 2.832e-47
```

Here are the regression coefficients for the CpG island terms of Model 3:

```
                 coef     se       z      p
HIV/H9, Hela  -0.3624 0.3783 -0.9579 0.3381
HIV/IMR90     -1.2348 0.4155 -2.9719 0.0030
HIV/PBMC      -2.3987 0.7136 -3.3615 0.0008
HIV/SupT1     -1.5124 0.5077 -2.9788 0.0029
ASLV/293T-TVA  0.5540 0.2668  2.0761 0.0379
MLV/Hela       1.7714 0.1210 14.6361 0.0000
```

Here is the analysis of deviance table for regions within 5 kilobases of a CpG island (or in the island):

```
Analysis of Deviance Table

Model 1 : Data Set : In Gene + express score per 500k
Model 2 : Data Set : In Gene + express score per 500k + CpG.5k
Model 3 : Data Set : In Gene + Data Set : express score per 500k + Data Set : CpG.5k
  Resid. Df Resid. Dev    Df Deviance P(>|Chi|)
1     34389    13488.1
2     34388    13459.4     1     28.7 8.293e-08
3     34383    13328.1     5    131.2 1.303e-26
```

Here are the regression coefficients for the CpG island terms of Model 3:

```
                 coef     se       z      p
HIV/H9, Hela   0.0662 0.1186  0.5585 0.5765
HIV/IMR90     -0.3088 0.1286 -2.4019 0.0163
HIV/PBMC      -0.3587 0.1301 -2.7576 0.0058
HIV/SupT1     -0.0869 0.1196 -0.7269 0.4673
ASLV/293T-TVA  0.3603 0.1066  3.3793 0.0007
MLV/Hela       0.8339 0.0724 11.5252 0.0000
```

Here is the analysis of deviance table for regions within 10 kilobases of a CpG island (or in the island):

```
Analysis of Deviance Table

Model 1 : Data Set : In Gene + express score per 500k
Model 2 : Data Set : In Gene + express score per 500k + CpG.10k
Model 3 : Data Set : In Gene + Data Set : express score per 500k + Data Set : CpG.10k
  Resid. Df Resid. Dev    Df Deviance P(>|Chi|)
1     34389    13488.1
2     34388    13448.9     1     39.2 3.898e-10
3     34383    13370.0     5     78.9 1.410e-15
```

Here are the regression coefficients for the CpG island terms of Model 3:

```
                 coef     se       z      p
HIV/H9, Hela   0.1963 0.0708  2.7709 0.0056
```

HIV/IMR90	-0.1750	0.0802	-2.1817	0.0291
HIV/PBMC	-0.0098	0.0709	-0.1376	0.8906
HIV/SupT1	0.0275	0.0707	0.3881	0.6980
ASLV/293T-TVA	0.2784	0.0724	3.8439	0.0001
MLV/Hela	0.5044	0.0514	9.8117	0.0000

Here is the analysis of deviance table for regions within 25 kilobases of a CpG island (or in the island):

```
Analysis of Deviance Table

Model 1 : Data Set : In Gene + express score per 500k
Model 2 : Data Set : In Gene + express score per 500k + CpG.25k
Model 3 : Data Set : In Gene + Data Set : express score per 500k + Data Set : CpG.25k
  Resid. Df Resid. Dev    Df Deviance P(>|Chi|)
1     34389    13488.1
2     34388    13450.9     1     37.2 1.087e-09
3     34383    13417.3     5     33.7 2.786e-06
```

Here are the regression coefficients for the CpG island terms of Model 3:

	coef	se	z	p
HIV/H9, Hela	0.1374	0.0379	3.6214	0.0003
HIV/IMR90	-0.0591	0.0405	-1.4588	0.1446
HIV/PBMC	0.0327	0.0358	0.9117	0.3619
HIV/SupT1	0.0778	0.0320	2.4316	0.0150
ASLV/293T-TVA	0.1387	0.0410	3.3794	0.0007
MLV/Hela	0.1833	0.0271	6.7529	0.0000

Here is the analysis of deviance table for regions within 50 kilobases of a CpG island (or in the island):

```
Analysis of Deviance Table

Model 1 : Data Set : In Gene + express score per 500k
Model 2 : Data Set : In Gene + express score per 500k + CpG.50k
Model 3 : Data Set : In Gene + Data Set : express score per 500k + Data Set : CpG.50k
  Resid. Df Resid. Dev    Df Deviance P(>|Chi|)
1     34389    13488.1
2     34388    13444.5     1     43.6 3.941e-11
3     34383    13412.1     5     32.4 4.934e-06
```

Here are the regression coefficients for the CpG island terms of Model 3:

	coef	se	z	p
HIV/H9, Hela	0.1048	0.0215	4.8651	0.0000
HIV/IMR90	-0.0277	0.0236	-1.1743	0.2403
HIV/PBMC	0.0116	0.0214	0.5432	0.5870

```
HIV/SupT1       0.0677 0.0185  3.6487 0.0003
ASLV/293T-TVA   0.0791 0.0248  3.1841 0.0015
MLV/Hela        0.0959 0.0156  6.1322 0.0000
```

Here is a plot of the relative intensity of integration (after accounting for the effects of being in a gene and the expression density) based on the regression coefficients above. The 'error bar' drawn with each colored bar indicates the range of the 95 percent confidence interval. Error bars that do not cross the horizontal line for relative intensity = 1.0 indicate preference for (or avoidance of) sites near CpG islands.

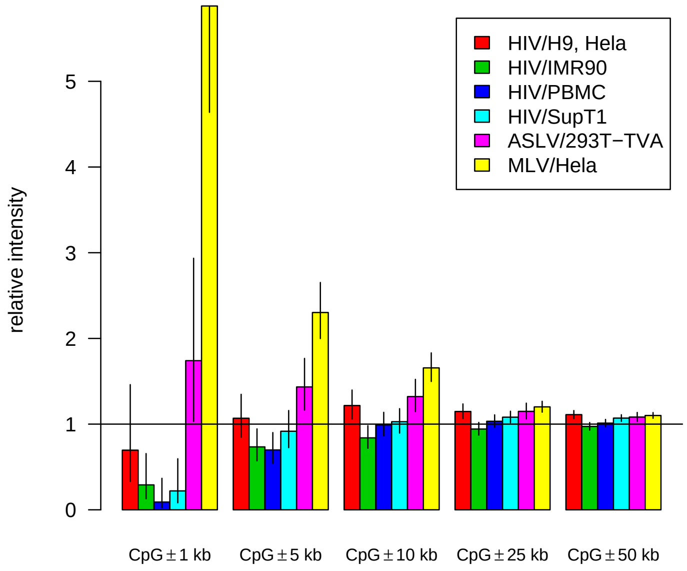

Evidently the effects are generally strongest near the CpG islands and tend to be in the direction of suppressing integration for HIV cells while increasing it for ASLV and MLV.

6 GC content

Using the annotations of GC content from
`http://genome.ucsc.edu/goldenPath/14nov2002/database/gcPercent.txt.gz`
we determined whether the GC content of the region surrounding the insertion site. Here is the analysis of deviance table taking a model that includes the effect of expression 'density' and data set specific effects of being in a gene as the point of departure:

```
Analysis of Deviance Table

Model 1 : Data Set : In Gene + express score per 500k
Model 2 : Data Set : In Gene + express score per 500k + GC content
Model 3 : Data Set : In Gene + Data Set : express score per 500k + Data Set : GC content
  Resid. Df Resid. Dev    Df Deviance P(>|Chi|)
1     34389    13488.1
2     34388    13458.6     1     29.5 5.558e-08
3     34383    13251.7     5    206.9 9.593e-43
```

Here are the regression coefficients for the GC content in terms of Model 3:

	coef	se	z	p
HIV/H9, Hela	-0.0340	0.0108	-3.1361	0.0017
HIV/IMR90	-0.0750	0.0098	-7.6234	0.0000
HIV/PBMC	-0.1006	0.0106	-9.4813	0.0000
HIV/SupT1	-0.0258	0.0094	-2.7314	0.0063
ASLV/293T-TVA	-0.0005	0.0103	-0.0506	0.9596
MLV/Hela	0.0482	0.0071	6.8215	0.0000

As with regions near CpG islands, there is a tendency of regions rich in GC nucleotides to attract MLV integration events and repel HIV integrations. There seems to be little or no effect on ASLV.

7 Combined Effects

Here the combined effects of gene density, expression density, intra-gene location, proximity to a CpG island (±1kb), GC content, and being in the first tenth of a gene (from the transcription start site) are studied. (Cytobands have negligible effects after these other variables are accounted for.) The following table shows the effect of dropping each term from a model that includes all of the others. Each data set is fitted separately to allow differential effects according to data set.

	Df	Deviance	P(>|Chi|)
drop.all	48	2188.76	0.00
drop.cpg	6	160.63	4.359e-32
drop.dens	12	53.78	2.992e-07
drop.expr	12	238.63	3.202e-44
drop.gcpct	6	272.48	6.390e-56
drop.gene	6	356.70	5.609e-74
drop.start	6	23.39	6.750e-04

The p value of '0.0' is not literally correct, but the number is too small to be computed using ordinary double precision arithmetic. It is worth pointing out that the sum of the deviances for each of the models obtained by dropping one

of the variables at a time is only about half of the value obtained for dropping all of them. This is due to correlation amongst regressor variables — particularly gene density and expression density, whose joints effects acount for roughly one third of the deviance explained by all variables.

The following table gives a somewhat more detailed view of these results. The proportion of deviance accounted for by the model that includes all terms in each of the cell lines is given by the 'fit.all' column, while each of the 'drop' columns gives the proportion of deviance accounted for by all terms but the one that is listed.

	drop.cpg	drop.dens	drop.expr	drop.gcpct	drop.gene	drop.start	fit.all
HIV/H9, Hela	0.178	0.170	0.159	0.164	0.151	0.178	0.178
HIV/IMR90	0.092	0.092	0.084	0.080	0.054	0.092	0.094
HIV/PBMC	0.222	0.222	0.203	0.177	0.174	0.227	0.227
HIV/SupT1	0.219	0.215	0.191	0.206	0.190	0.222	0.224
ASLV/293T-TVA	0.035	0.036	0.029	0.035	0.034	0.037	0.037
MLV/Hela	0.095	0.128	0.121	0.117	0.128	0.125	0.129

Perhaps it is of some interest that the proportion of deviance accounted for in ASLV is much smaller than in any other cell line. Also, it is rare to find that omitting a single term has much effect on the deviance; exceptions to this are the effect of being in a gene for HIV lines, being within 1kb of a CpG island for MLV, and GC content for HIV/PBMC.

This table compares the proportions of deviance accounted for by the model with all the variables in the different cell lines and tests the significance of those differences (via the Wilcoxon rank sum test):

cell lines		diff of prop of deviance	p-value
HIV/IMR90	HIV/H9, Hela	-0.085	1.056609e-07
HIV/PBMC	HIV/H9, Hela	0.049	4.258347e-03
HIV/SupT1	HIV/H9, Hela	0.045	5.876371e-03
ASLV/293T-TVA	HIV/H9, Hela	-0.142	0.000000e+00
MLV/Hela	HIV/H9, Hela	-0.050	5.901745e-05
HIV/H9, Hela	HIV/IMR90	0.085	1.056609e-07
HIV/PBMC	HIV/IMR90	0.133	8.472295e-20
HIV/SupT1	HIV/IMR90	0.130	5.989913e-16
ASLV/293T-TVA	HIV/IMR90	-0.057	3.893367e-08
MLV/Hela	HIV/IMR90	0.035	7.966515e-01
HIV/H9, Hela	HIV/PBMC	-0.049	4.258347e-03
HIV/IMR90	HIV/PBMC	-0.133	0.000000e+00
HIV/SupT1	HIV/PBMC	-0.003	8.092727e-01
ASLV/293T-TVA	HIV/PBMC	-0.190	0.000000e+00
MLV/Hela	HIV/PBMC	-0.098	1.487699e-14
HIV/H9, Hela	HIV/SupT1	-0.045	5.876371e-03
HIV/IMR90	HIV/SupT1	-0.130	6.661338e-16
HIV/PBMC	HIV/SupT1	0.003	8.092727e-01

ASLV/293T-TVA	HIV/SupT1	-0.187	0.000000e+00
MLV/Hela	HIV/SupT1	-0.095	1.226610e-10
HIV/H9, Hela	ASLV/293T-TVA	0.142	3.431275e-19
HIV/IMR90	ASLV/293T-TVA	0.057	3.893367e-08
HIV/PBMC	ASLV/293T-TVA	0.190	4.969135e-40
HIV/SupT1	ASLV/293T-TVA	0.187	1.641630e-29
MLV/Hela	ASLV/293T-TVA	0.092	8.627285e-05
HIV/H9, Hela	MLV/Hela	0.050	5.901745e-05
HIV/IMR90	MLV/Hela	-0.035	7.966515e-01
HIV/PBMC	MLV/Hela	0.098	1.496529e-14
HIV/SupT1	MLV/Hela	0.095	1.226609e-10
ASLV/293T-TVA	MLV/Hela	-0.092	8.627285e-05

References

[1] P. McCullagh and J. A. Nelder. *Generalized Linear Models (Second Edition)*. Chapman & Hall, 1989.

[2] Rogier. Versteeg, Barbera. D. C. van Schaik., Marinus. F. van Batenburg., Marco. Roos, Ramin. Monajemi, Huib. Caron, Harmen. J. Bussemaker, and Antoine. H. C. van Kampen. The human transcriptome map reveals extremes in gene density, intron length, GC content, and repeat pattern for domains of highly and weakly expressed genes. *Genome Res*, 13(9):1998–2004, Sep 2003.

[3] Xiaolin. Wu, Yuan. Li, Bruce. Crise, and Shawn. M. Burgess. Transcription start regions in the human genome are favored targets for MLV integration. *Science*, 300(5626):1749–1751, Jun 2003.
